# New Dihydroisocoumarin Root Growth Inhibitors From the Sponge-Derived Fungus *Aspergillus* sp. NBUF87

**DOI:** 10.3389/fmicb.2019.02846

**Published:** 2019-12-10

**Authors:** Liming Huang, Lijian Ding, Xiaohui Li, Ning Wang, Wei Cui, Xiao Wang, C. Benjamin Naman, J. Enrico H. Lazaro, Xiaojun Yan, Shan He

**Affiliations:** ^1^Li Dak Sum Yip Yio Chin Kenneth Li Marine Biopharmaceutical Research Center, College of Food and Pharmaceutical Sciences, Ningbo University, Ningbo, China; ^2^Institute of Drug Discovery Technology, Ningbo University, Ningbo, China; ^3^Zhejiang Provincial Key Laboratory of Pathophysiology, School of Medicine, Ningbo University, Ningbo, China; ^4^Center for Marine Biotechnology and Biomedicine, Scripps Institution of Oceanography, University of California, San Diego, La Jolla, CA, United States; ^5^National Institute of Molecular Biology and Biotechnology, University of the Philippines Diliman, Quezon, Philippines

**Keywords:** dihydroisocoumarin, root growth inhibitor, sponge-derived fungus, *Aspergillus* sp., electronic circular dichroism

## Abstract

Six new dihydroisocoumarins, aspergimarins A−F (**1**−**6**), were discovered together with five known analogs (**7**−**11**) from a monoculture of the sponge-derived fungus *Aspergillus* sp. NBUF87. The structures of these compounds were elucidated through comprehensive spectroscopic methods, and absolute configurations were assigned after X-ray crystallography, use of the modified Mosher’s method, and comparison of electronic circular dichroism (ECD) data with literature values for previously reported analogs. Compounds **1**−**11** were evaluated in a variety of bioassays, and at 100 μM, both **1** and **5** showed significant inhibitory effects on the lateral root growth of *Arabidopsis thaliana* Columbia-0 (Col-0). Moreover, at 100 μM, **5** also possessed notable inhibition against the primary root growth of Col-0. Meanwhile, **1**−**11** were all found to be inactive *in vitro* against acetylcholinesterase (AChE) (IC_50_ > 100 μM), four different types of human-derived cancer cell lines (IC_50_ > 50 μM), as well as methicillin-resistant *Staphylococcus aureus* and *Escherichia coli* (MIC > 50 μg/mL), and *Plasmodium falciparum* W2 (EC_50_ > 100 μg/mL), in phenotypic tests.

## Introduction

Sponges are among the most primitive multicellular invertebrates and harbor vast microbial populations, owing largely to the unique filter-feeding physiology that is full of pores and channels (Hentschel et al., 2006; Webster and Taylor, 2012). As a result of the long-standing interaction and coevolution with sponges, marine symbiotic microorganisms have differentiated from those of terrestrial origins in terms of their biosynthetic pathways that lead to the production of structurally interesting and biologically active compounds (Thomas et al., 2010; Fan et al., 2012). Therefore, sponge-associated microbes have become an exciting area of drug discovery research (Thomas et al., 2010; Pita et al., 2016). Endophytic fungi that are associated with sponges, especially members of the genus *Aspergillus*, have been recognized as a source of structurally diverse natural products with biological activities that provide value for drug discovery (Blunt et al., 2018; Zhang et al., 2018). In the past decade, the secondary metabolites from sponge-derived *Aspergillus* fungi have been reported from many classes, including polyketides (Wang et al., 2014; Kong et al., 2015), terpenoids (Liu et al., 2009; Li D. et al., 2012), alkaloids (Zhou et al., 2013, 2014), diketopiperazines (Ahmed et al., 2017), and peptides (Lee et al., 2011). Many of these metabolites have been shown to exhibit strong antitumor, antibacterial, antiviral, and other bioactivities.

Isocoumarins and 3,4-dihydroisocoumarins, subclasses of polyketide compounds, are also lactone-containing natural products that are abundantly produced among fungi, bacteria, liverworts, lichens, as well as some higher plants (Elsebai and Ghabbour, 2016; Saeed, 2016; Hussain and Green, 2017; Chen M. et al., 2019). Moreover, these compounds have been isolated from marine sponges, insect pheromones, and venoms (Saeed, 2016). Almost 400 isocoumarins and dihydroisocoumarins have been reported to date, and these compounds have been found to be of broad interest across many pharmacological applications (Saeed, 2016; Chen M. et al., 2019). For example, isocoumarin derivatives from some marine-derived fungi are found to possess a wide range of biological properties including enzyme inhibitory (Kim et al., 2015; Chen S. et al., 2016; Wiese et al., 2016; Cai et al., 2018), cytotoxic (Wang et al., 2019; Wu et al., 2019), antibacterial (Li S. et al., 2012; Lei et al., 2017; Chen Y. et al., 2018; Wang et al., 2019), antiproliferative (Tsukada et al., 2011), anti-food allergic (Niu et al., 2018), as well as anti-inflammatory (Kim et al., 2015; Chen Y. et al., 2018; Liu et al., 2018) activities.

**FIGURE 1:**
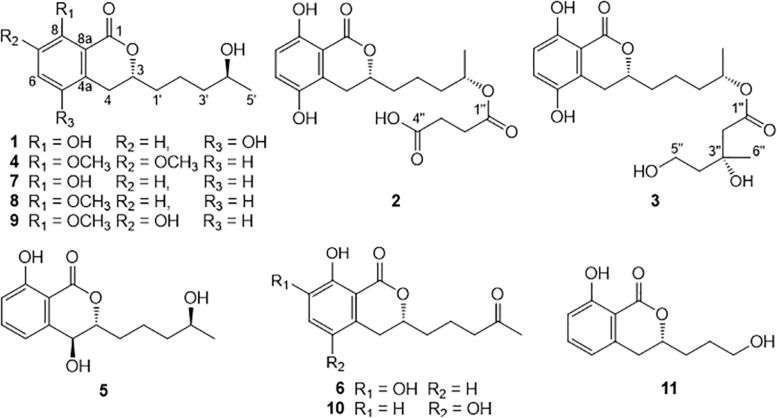
Structures of isolated dihydroisocoumarin derivatives (**1**–**11**).

As part of a continuing research program investigating the biologically active secondary metabolites from sponge-derived fungi (Ding et al., 2018; Huang et al., 2019; Li W. et al., 2019), a detailed chemical investigation was initiated on the culture of fungus *Aspergillus* sp. NBUF87. The fungus was isolated from a South China Sea marine sponge of the genus *Hymeniacidon*. The EtOAc extract of the culture of fungus *Aspergillus* sp. NBUF87 exhibited inhibitory effects on the root growth of *Arabidopsis thaliana* Columbia-0 (Col-0), a typical model organism for studying plant growth and development. The separation and purification of the bioactive extract led to the discovery of six new dihydroisocoumarin compounds (**1**−**6**) and five known analogs (**7**−**11**) (Figure 1). Herein, the detailed isolation and structure elucidation of these dihydroisocoumarin derivatives, together with the evaluation of their inhibitory effects against the root growth of Col-0 and a preliminary broader biological activity screening are described.

## Materials and Methods

### General Experimental Procedures

Optical rotation measurements were conducted with a JASCO P-2000 digital polarimeter. IR and UV spectra were obtained with a Thermo Scientific Nicolet iS5 FT-IR spectrometer and a Thermo Scientific Evolution 201 spectrophotometer, respectively. Electronic circular dichroism (ECD) spectra were collected on a JASCO J-1500 spectrophotometer. 1D and 2D NMR spectra were recorded in DMSO-*d*_6_ (or CDCl_3_) with a Palo Alto Varian 600 MHz spectrometer, using standard pulse sequences. HRESIMS data were collected on an Agilent Technologies 6224 TOF MS. X-ray single-crystal diffraction data were acquired using an Agilent Gemini Ultra diffractometer with Cu Kα radiation (λ = 1.54178 Å). Medium-pressure liquid chromatography (MPLC) was performed using a Bonna-Agela FLEXA purification instrument. Column chromatography (CC) was carried out with silica gel (200−300 mesh, Qingdao) and Amersham Biosciences Sephadex LH-20. Reversed-phase HPLC (RP-HPLC) was conducted using a Waters 1525 binary HPLC pump equipped with a Waters 2996 photodiode array detector and a YMC-Pack C18 column (YMC, 20 × 250 mm, 5 μm).

### Fungal Material

The fungus *Aspergillus* sp. NBUF87 was isolated from the sponge *Hymeniacidon* sp. obtained from the Paracel Islands in the South China Sea, and was determined as being *Aspergillus* sp. by its morphology and gene sequence (ITS rDNA region) analyses (GenBank accession no. MH595747.1). The strain specimen was deposited in PDB medium to the repository conserved at the College of Food and Pharmaceutical Sciences, Ningbo University, China.

### Fermentation, Extraction, and Isolation

Spores of *Aspergillus* sp. NBUF87 were initially inoculated into Erlenmeyer flasks (1 L) containing 400 mL of the seed medium (potato dextrose broth powder 26 g/L and sea salt 35 g/L dissolved in distilled water) and were grown on a shaker (150 r/min) for 100 h at 28°C. The subsequent amplified fermentation was conducted in 105 × 1 L Erlenmeyer flasks, each containing solid rice medium (rice 120 g, sea salt 6.3 g, purified H_2_O 180 mL), followed by inoculation with 30 mL of the seed culture. After fermentation at room temperature under static conditions for 6 weeks, the fermented substrate was repeatedly extracted with EtOAc. Removal of EtOAc under reduced pressure yielded 54 g of crude extract, which was subjected to vacuum liquid chromatography (VLC) on silica gel column eluting with a petroleum ether/EtOAc stepwise gradient system (from 1:0 to 0:1) to generate seven fractions (Fr. 1−7).

Fraction 4 (3.9 g) was subjected to Sephadex LH-20 gel filtration chromatography, eluted with isocratic CH_2_Cl_2_-CH_3_OH (1:1, v/v) to furnish five sub-fractions (Fr. 4A−4F). The further separation of Fr. 4E (1.8 g) was conducted by reversed-phase ODS MPLC with a gradient elution of CH_3_OH/H_2_O (from 25 to 100% CH_3_OH, flow rate 20 mL/min, 180 min, UV detection at 210 nm) to afford 60 test tube sub-fractions. Subsequently, aspergimarin A (**1**, 15.3 mg, *t*_R_ 33.4 min) was purified from Fr. 4E-20 by semi-preparative RP-HPLC [YMC-Pack C18 column (YMC, 20 × 250 mm, 5 μm), UV detection at 210 and 246 nm] with 28% CH_3_CN/H_2_O at 2 mL/min. Aspergimarin E (**5**, 3.6 mg, *t*_R_ 40.2 min), compounds **10** (4.3 mg, *t*_R_ 72.3 min) and **11** (4.7 mg, *t*_R_ 58.2 min) were purified from Fr. 4E-19 by semi-preparative RP-HPLC with 24% CH_3_CN/H_2_O at 2 mL/min. Aspergimarin F (**6**, 3.4 mg, *t*_R_ 57.3 min) was purified from Fr. 4E-18 by semi-preparative RP-HPLC with 24% CH_3_CN/H_2_O at 2 mL/min. Fr. 4B (1.0 g) was conducted by MPLC (30−90% CH_3_OH/H_2_O, flow rate 20 mL/min, 120 min, UV detection at 210 nm) to produce 10 sub-fractions (Fr. 4B-1−4B-10). Fr. 4B-4 was further purified by semi-preparative RP-HPLC with 42% CH_3_OH/H_2_O at 2 mL/min to yield compound **9** (43.1 mg, *t*_R_ 45.0 min). Fr. 4B-8 was further purified by semi-preparative RP-HPLC with 60% CH_3_OH/H_2_O at 2 mL/min to afford compound **7** (56.0 mg, *t*_R_ 26.7 min) (Supplementary Figures S1–S10, S41–S60).

Fraction 5 (4.8 g) was separated by Sephadex LH-20 gel filtration chromatography utilizing an isocratic elution gradient of CH_2_Cl_2_−CH_3_OH (1:1, v/v) to afford three sub-fractions (Fr. 5A−5C). The further separation of Fr. 5B (2.5 g) was conducted by RP-MPLC (ODS, 15−100% CH_3_OH/H_2_O, flow rate 20 mL/min, 140 min, UV detection at 210 nm) to give 35 tubes. Aspergimarin B (**2**, 2.5 mg, *t*_R_ 31.4 min) and aspergimarin C (**3**, 3.8 mg, *t*_R_ 22.9 min) were further purified from Fr.5B-23 by semi-preparative RP-HPLC with 30% CH_3_CN/H_2_O at 4 mL/min. Fr. 5A (0.9 g) was separated by MPLC (ODS, 25−100% CH_3_OH/H_2_O, flow rate 20 mL/min, 150 min, UV detection at 210 nm) to obtain 38 tubes. Fr.5A-15 was further purified by semi-preparative RP-HPLC with 25% CH_3_CN/H_2_O at 4 mL/min to afford aspergimarin D (**4**, 6.3 mg, *t*_R_ 31.0 min). Fr. 5A-14 was purified by semi-preparative RP-HPLC with 22% CH_3_CN/H_2_O at 4 mL/min to afford compound **8** (26.1 mg, *t*_R_ 31.6 min) (Supplementary Figures S11–S40).

*Aspergimarin A* (***1***): white, crystals; mp 177.9−178.8 °C; [α]20 D −34.0 (*c* 0.2, CHCl_3_); UV (MeOH) λ_max_ (log ε): 219 (4.14), 248 (3.77), 346 (3.58) nm; CD (*c* = 0.68 mM, MeOH) λ_max_ (Δε): 223 (+ 2.15), 237 (−1.93), 245 (−1.10), 259 (−6.32) nm; IR (KBr) ν_max_: 3205, 1647, 1588, 1483, 1354, 1281, 820, 795, 700 cm^–1^; ^1^H and ^13^C NMR data, see Table 1; HRESIMS *m/z* 265.1082 [M − H] ^–^ (calcd for C_14_H_17_O_5_, 265.1081).

**TABLE 1:** ^1^H (600 MHz) and ^13^C (150 MHz) NMR data of **1**−**3** collected in DMSO-*d*_6_.

**Position**	**1**		**2**		**3**	
	**δ_H_ (*J* in Hz)**	**δ_C_, type**	**δ_H_ (*J* in Hz)**	**δ_C_, type**	**δ_H_ (*J* in Hz)**	**δ_C_, type**
1		169.5, C		169.5, C		169.5, C
3	4.59, m	79.4, CH	4.59, m	79.2, CH	4.59, m	79.2, CH
4	3.06, dd (16.9, 3.4) 2.60, dd (16.9, 11.6)	26.3, CH_2_	3.05, dd (16.9, 3.3) 2.60, dd (16.9, 11.5)	26.4, CH_2_	3.05, dd (16.9, 3.4) 2.60, dd (16.9, 11.5)	26.3, CH_2_
4a		124.5, C		124.0, C		124.5, C
5		146.6, C		145.7, C		145.6, C
6	7.07, d (8.9)	123.9, CH	7.06, d (8.9)	124.0, CH	7.06, d (8.9)	123.9, CH
7	6.72, d (8.9)	115.1, CH	6.72, d (8.9)	115.2, CH	6.72, d (8.9)	115.2, CH
8		153.9, C		153.9, C		153.9, C
8a		108.2, C		108.3, C		108.2, C
1′	1.78, m 1.68, m	34.3, CH_2_	1.76, m 1.70, m	33.9, CH_2_	1.74, m	33.9, CH_2_
2′	1.46, m	20.8, CH_2_	1.44, m	20.3, CH_2_	1.46, m	20.3, CH_2_
3′	1.36, m	38.6, CH_2_	1.55, m	34.9, CH_2_	1.55, m	34.9, CH_2_
4′	3.60, m	65.6, CH	4.83, m	70.2, CH	4.83, m	69.8, CH
5′	1.05, d (6.2)	23.7, CH_3_	1.16, d (6.3)	19.8, CH_3_	1.17, d (6.2)	19.8, CH_3_
1′′				171.8, C		170.4, C
2′′			2.45, m 1.24, m	28.9, CH_2_	2.38, m	46.9, CH_2_
3′′			2.46, m	29.1, CH_2_		69.8, C
4′′				173.5, C	1.67, m	43.7, CH_2_
5′′					3.54, m	57.2, CH_2_
6′′					1.18, s	27.4, CH_3_
8-OH	10.38, s		10.37, s		10.37, s	
3′′-OH					4.53, s	
5′′-OH					4.36, s	

*s, singlet; d, doublet; dd, doublet of doublets; m, multiplet.*

*Aspergimarin B* (***2***): brown, oil; [α]24 D −10.6 (*c* 0.2, MeOH); UV (MeOH) λ_max_ (log ε): 211 (4.24), 345 (3.66) nm; CD (*c* = 0.33 mM, MeOH) λ_max_ (Δε): 225 (+ 0.88), 235 (−1.54), 243 (−0.81), 257 (−5.77) nm; IR (KBr) ν_max_: 3226, 2929, 1723, 1675, 1469, 1379, 1207, 829 cm^–1^; ^1^H and ^13^C NMR data, see Table 1; HRESIMS *m/z* 365.1232 [M − H] ^–^ (calcd for C_18_H_21_O_8_, 365.1242).

*Aspergimarin C* (***3***): brown, oil; [α]24 D −10.8 (*c* 0.3, MeOH); UV (MeOH) λ_max_ (log ε): 219 (4.27), 344 (3.63) nm; CD (*c* = 0.45 mM, MeOH) λ_max_ (Δε): 229 (+ 0.86), 238 (−1.44), 243 (−0.32), 257 (−5.37) nm; IR (KBr) ν_max_: 3368, 2921, 1671, 1469, 1378, 1287, 1206, 1123 cm^–1^; ^1^H and ^13^C NMR data, see Table 1; HRESIMS *m/z* 435.1416 [M + K]^+^ (calcd for C_20_H_28_KO_8_, 435.1415).

*Aspergimarin D* (***4***): yellow, oil; [α]24 D −82.2 (*c* 0.5, MeOH); UV (MeOH) λ_max_ (log ε): 214 (4.16), 312 (3.24) nm; CD (*c* = 0.68 mM, MeOH) λ_max_ (Δε): 221 (−6.98), 241 (−0.28), 256 (−2.47), 284 (−0.48), 311 (−1.61) nm; IR (KBr) ν_max_: 3402, 2935, 1717, 1489, 1455, 1418, 1259, 1131, 1052, 963 cm^–1^; ^1^H and ^13^C NMR data, see Table 2; HRESIMS *m/z* 295.1537 [M + H]^+^ (calcd for C_16_H_23_O_5_, 295.1540).

**TABLE 2:** ^1^H (600 MHz) and ^13^C (150 MHz) NMR data of **4**–**6**.

**Position**	**4 (DMSO-*d*_6_)**		**5 (CDCl_3_)**		**6 (DMSO-*d*_6_)**	
	**δ_H_ (*J* in Hz)**	**δ_C_, type**	**δ_H_ (*J* in Hz)**	**δ_C_, type**	**δ_H_ (*J* in Hz)**	**δ_C_, type**
1		161.5, C		168.7, C		169.8, C
3	4.37, m	78.1, CH	4.47, m	83.6, CH	4.61, m	79.9, CH
4	2.91, dd (16.1, 3.1) 2.76, dd (16.1, 11.2)	32.7, CH_2_	4.71, d (7.5)	67.5, CH	2.91, dd (16.2, 3.3) 2.80, dd (16.2, 11.4)	31.4, CH_2_
4a		132.3, C		141.7, C		129.3, C
5	7.06, d (8.3)	122.7, CH	7.03, d (7.4)	116.3, CH	6.63, d (8.0)	117.5, CH
6	7.28, d (8.3)	117.7, CH	7.54, t (8.4, 7.4)	137.0, CH	7.00, d (8.0)	121.6, CH
7		152.2, C	6.69, d (8.4)	117.9, CH		144.4, C
8		150.1, C		162.1, C		150.0, C
8a		119.0, C		106.8, C		108.4, C
1′	1.70, m 1.60, m	34.1, CH_2_	1.87, m 1.82, m	31.5, CH_2_	1.71, m 1.63, m	33.3, CH_2_
2′	1.44, m	20.9, CH_2_	1.71, m 1.63, m	21.0, CH_2_	1.65, m 1.57, m	18.7, CH_2_
3′	1.34, m	38.7, CH_2_	1.51, m	38.7, CH_2_	2.51, t (6.2)	42.1, CH_2_
4′	3.59, m	65.7, CH	3.85, m	67.9, CH		208.2, C
5′	1.04, d (6.1)	23.7, CH_3_	1.21, d (6.2)	23.9, CH_3_	2.08, s	29.8, CH_3_
7-OCH_3_	3.81, s	56.1, CH_3_				
8-OCH_3_	3.75, s	60.7, CH_3_				
8-OH			10.97, s			

*s, singlet; d, doublet; dd, doublet of doublets; t, triplet; m, multiplet.*

*Aspergimarin E* (***5***): colorless, oil; [α]24 D + 17.7 (*c* 0.3, MeOH); UV (MeOH) λ_max_ (log ε): 209 (4.17), 246 (3.52), 312 (3.42) nm; CD (*c* = 0.56 mM, MeOH) λ_max_ (Δε): 206 (−8.37), 218 (−0.47), 224 (−1.04), 243 (+ 3.53), 264 (−0.71), 280 (−0.01), 311 (−0.53) nm; IR (KBr) ν_max_: 3367, 2920, 1672, 1461, 1230, 1117, 823 cm^–1^; ^1^H and ^13^C NMR data, see Table 2; HRESIMS *m/z* 289.1034 [M + Na]^+^ (calcd for C_14_H_18_NaO_5_, 289.1046).

*Aspergimarin F* (***6***): yellow, amorphous powder; [α]24 D −7.5 (*c* 0.2, MeOH); UV (MeOH) λ_max_ (log ε): 221 (4.14), 256 (3.71), 332 (3.44) nm; CD (*c* = 0.57 mM, MeOH) λ_max_ (Δε): 208 (−8.75), 240 (+ 2.67), 266 (−1.47) nm; IR (KBr) ν_max_: 3360, 2920, 2849, 2361, 1707, 1664, 1452, 1272, 1135, 668 cm^–1^; ^1^H and ^13^C NMR data, see Table 2; HRESIMS *m/z* 287.0879 [M + Na]^+^ (calcd for C_14_H_16_NaO_5_, 287.0890).

### X-Ray Crystal Structure Analysis of **1**

A single crystal of **1** was obtained from 90% CH_3_OH/H_2_O. Crystal X-ray diffraction data was collected on an Agilent Gemini Ultra diffractometer with Cu Kα radiation (λ = 1.54178 Å). The structure was solved by direct methods (SHELXS−97) and refined with full-matrix least-squares difference Fourier techniques. All non-hydrogen atoms were refined anisotropically, and hydrogen atoms were placed in the idealized geometrical positions and refined isotropically with a riding model. Crystallographic data for **1** have been deposited in the Cambridge Crystallographic Data Center as supplementary publication no. CCDC 1919904. Copies of the data can be obtained, free of charge, on application to the Director, CCDC, 12 Union Road, Cambridge CB21EZ, United Kingdom (fax: + 44-(0)1223-336033, or e-mail: deposit@ccdc.cam.ac.uk).

*Crystal data of aspergimarin A* (***1***): C_14_H_18_O_5_, *M*_R_ = 266.28, monoclinic, *a* = 4.9195(3) Å, *b* = 24.7434(17) Å, *c* = 5.6478(4) Å, α = γ = 90°, β = 101.812(2)°, *V* = 672.92(8) Å^3^, space group *P*2_1_, *Z* = 2, *D*_c_ = 1.314 mg/m^3^, μ = 0.829 mm^–1^, and *F*(000) = 284. Crystal size: 0.200 × 0.170 × 0.130 mm^3^. Independent reflections: 2294 (*R*_int_ = 0.0377). Final *R* indices [*I* > 2 sigma (*I*)], *R*_1_ = 0.0717, *wR*_2_ = 0.2076. Goodness of fit on *F*^2^ was 1.109.

### Preparation of MTPA Esters of **3**−**5** for Modified Mosher’s Analysis

Under an atmosphere of nitrogen, pyridine-*d*_5_ (500 μL) and (*R*)-MTPA-Cl (8 μL) was sequentially added to an EP tube containing compounds **3**, **4**, or **5** (1.0 mg), separately. The mixture was shaken at 28 °C for 12 h and then purified by RP-HPLC to obtain the (*S*)-MTPA esters **3a**, **4a**, and **5a**. By the same procedure, the (*R*)-MTPA esters **3b**, **4b**, and **5b** were obtained using (*S*)-MTPA-Cl as a reagent. Key ^1^H NMR signals used for configurational assignments were determined by respective ^1^H−^1^H COSY correlations and the already completed full assignments of ^1^H NMR data for **3**, **4**, and **5** (see Supplementary Figures S61–S72).

The C-3′′ absolute configuration of **3** were established as *R* on the basis of the Δδ values (Δδ_H__–__2__^″_: −0.01; Δδ_H__–__4__^″_: + 0.03; Δδ_H__–__5__^″_: + 0.01) of the (*S*)- and (*R*)-MTPA esters (**3a** and **3b**). **3a**: H-2′′ (δ_H_ 2.37), H-4′′ (δ_H_ 1.89), H-5′′ (δ_H_ 4.45); **3b**: H-2′′ (δ_H_ 2.38), H-4′′ (δ_H_ 1.86), H-5′′ (δ_H_ 4.44).

The C-4′ absolute configuration of **4** was established as *S* on the basis of the Δδ values (Δδ_H__–__3__′_: + 0.05, + 0.05; Δδ_H__–__5_−0.10) of the (*S*)– and (*R*)–MTPA esters (**4a** and **4b**). **4a**: H–3′ (δ_H_ 1.64, 1.27), H-5′ (δ_H_ 1.21); **4b**: H-3′ (δ_H_ 1.59, 1.22), H-5′ (δ_H_ 1.31).

Both C-4 and C-4′ absolute configuration of **5** were established as *S* on the basis of the Δδ values (Δδ_H–3_: + 0.17; Δδ_H–5_: −0.19; Δδ_H–6_: −0.02; Δδ_H–1ΔδH–2ΔδH–3ΔδH–5_−0.07) of the (*S*)– and (*R*)–MTPA esters (**5a** and **5b**). **5a**: H–3 (δ_H_ 4.65), H-5 (δ_H_ 7.27), H-6 (δ_H_ 7.63), H-1′ (δ_H_ 1.66, 1.53), H-2′ (δ_H_ 1.49), H-3′ (δ_H_ 1.60, 1.32), H-5′ (δ_H_ 1.25); **5b**: H-3 (δ_H_ 4.48), H-5 (δ_H_ 7.46), H-6 (δ_H_ 7.65), H-1′ (δ_H_ 1.58, 1.40), H-2′ (δ_H_ 1.46), H-3′ (δ_H_ 1.53, 1.24), H-5′ (δ_H_ 1.32).

### Plant Growth Response Assays

*Arabidopsis thaliana* Col-0, a model organism for plant growth and development, was used to test each isolated compound according to a previously described protocol (Li X. et al., 2018; Huang et al., 2019). Plants grown in 2% (*v/v*) DMSO were used as the negative control. Seeds of Col-0 incubated in 1 μM of 6-benzylaminopurine (BAP) were selected as the positive control. Test samples were dissolved in 2% (*v/v*) DMSO at various test concentrations for the experiment.

### *In vitro* AChE Activity Assays

For compounds **1**−**11**, the *in vitro* acetylcholinesterase (AChE) activity was assessed by the colorimetric method in 96-well plates according to a previously reported method (Santos et al., 2012). Donepezil was selected as the positive control with IC_50_ value of 11.9 nM (Chen H. et al., 2018).

### *In vitro* Cancer Cell Cytotoxicity Assays

The *in vitro* cytotoxic activities of all isolated compounds against four human cancer cell lines (CCRF-CEM, MDA-MB-231, HCT-116, and AGS) were evaluated by the MTT method as previously described (Huang et al., 2019; Li W. et al., 2019). 7-Ethyl-10-hydroxycamptothecin (1.3, 10.8, 9.9, and 4.2 nM, respectively) was used as the positive control against four above-mentioned human cancer cell lines.

### Antibacterial Activity Assays

Compounds **1**−**11** were evaluated for their antibacterial activities against methicillin-resistant *Staphylococcus aureus* ATCC43300 and *Escherichia coli* ATCC25922 in 96-well plates according to the method described by Gu et al. (2018). Ciprofloxacin was selected as a positive control against the above-mentioned bacteria with MIC values of 0.5 μg/mL.

### *In vitro* Antimalarial Activity Assays

The *in vitro* antimalarial activity of the compounds was evaluated against the parasite (*Plasmodium falciparum* W2), which was cultured continuously according to a previously described method (Lazaro et al., 2006). Chloroquine, atovaquone, and artemisinin were used as positive controls against the above parasite with EC_50_ values of 112, 2.5, and 160 nM, respectively.

## Results and Discussion

### Structure Elucidation

**FIGURE 2:**
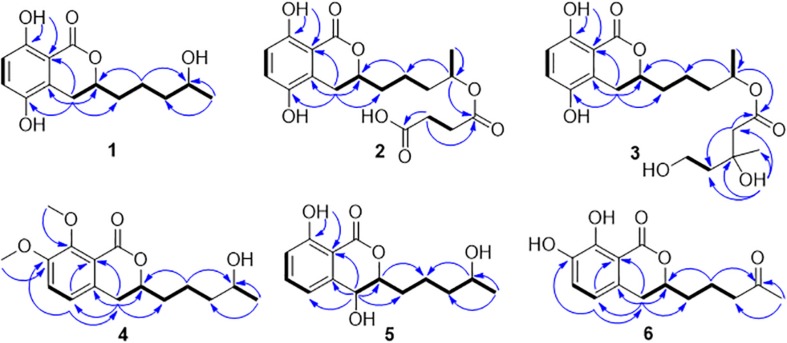
^1^H−^1^H COSY and key HMBC correlations observed for **1**−**6**.

**FIGURE 3:**
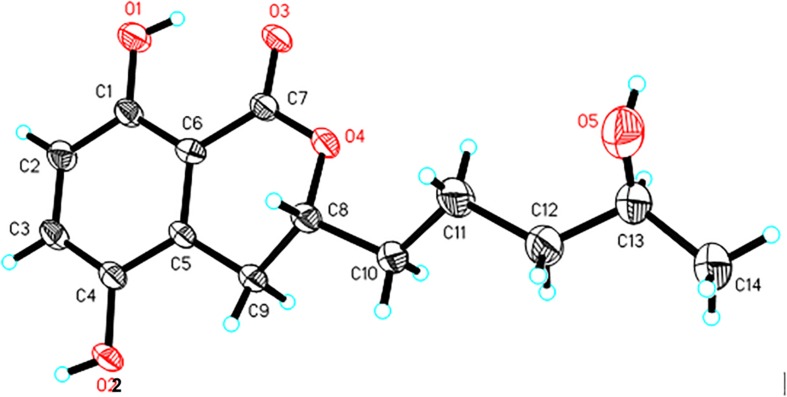
X-ray ORTEP drawing of **1.**

Compound **1** was isolated as white crystals. The molecular formula of **1** was determined to be C_14_H_18_O_5_ based on an ion peak observed in HRESIMS spectrum (*m/z* [M - H]^–^ 265.1082, calcd for C_14_H_17_O_5_, 265.1081), and this implied the compound has six degrees of unsaturation. The ^1^H NMR data of **1** (Table 1) demonstrated two aromatic signals [(δ_H_ 7.07, d, *J* = 8.9 Hz, H-6) and (δ_H_ 6.72, d, *J* = 8.9 Hz, H-7)] that were suggestive of two *ortho* protons. In addition, one phenolic hydroxy group at δ_H_ 10.38 (1H, s, 8-OH), two oxygenated methine groups at δ_H_ 4.59 (1H, m, H-3) and 3.60 (1H, m, H-4′), four methylene groups at δ_H_ 3.06 (1H, dd, H-4α), 2.60 (1H, dd, H-4β), 1.78 (1H, m, H-1′α), 1.68 (1H, m, H-1′β), 1.46 (2H, m, H-2′), and 1.36 (2H, m, H-3′), and one methyl group at δ_H_ 1.05 (3H, d, H-5′) were observed in the ^1^H NMR spectrum of **1**. The ^13^C NMR and DEPT spectra together for **1** indicated 14 carbon signals (Table 1), including a carbonyl at δ_C_ 169.5 (C-1), six aromatics at δ_C_ 153.9 (C-8), 146.6 (C-5), 124.5 (C-4a), 123.9 (C-6), 115.1 (C-7), and 108.2 (C-8a), and seven alkyl carbons that were two oxygenated methines at δ_C_ 79.4 (C-3) and 65.6 (C-4′), four hydrocarbon methylenes at δ_C_ 38.6 (C-3′), 34.3 (C-1′), 26.3 (C-4), and 20.8 (C-2′), and a methyl at δ_C_ 23.7 (C-5′). In total, the NMR data suggested the presence of a dihydroisocoumarin skeleton contained in **1**. The ^1^H−^1^H COSY correlations of H-5′/H-4′/H-3′/H-2′/H-1′ further indicated a continuous spin system in the molecule, identified as -CH_2_-CH_2_-CH_2_-CH(OH)-CH_3_ (Figure 2). Furthermore, according to the key HMBC correlations presented in Figure 2, including from H-2′ to C-3, from H-4 to C-1′, C-3, C-5, C-4a, and C-8a, as well as from 8-OH to C-7, C-8, and C-8a, the planar structure of **1** was established as shown. Finally, on the basis of X-ray single-crystal diffraction analysis (Figure 3), the absolute configuration of **1** was established as 3*R*,4′*S*, and this new molecule was given the trivial name aspergimarin A.

**FIGURE 4:**
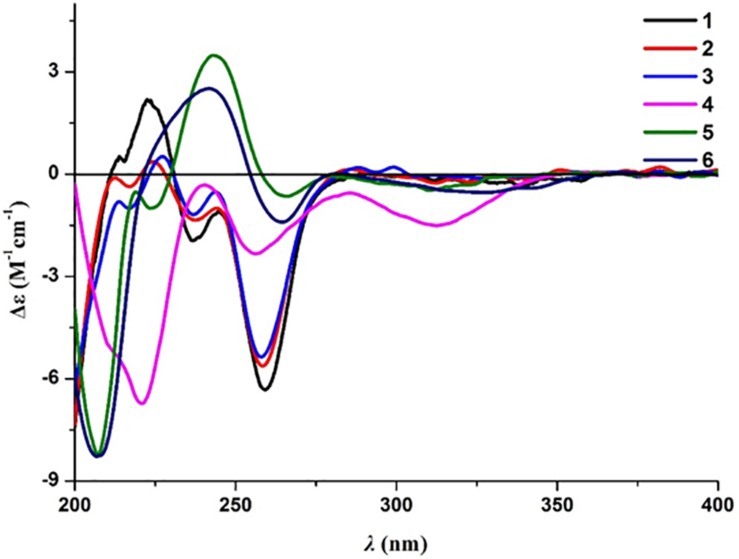
Experimental ECD spectra of **1**−**6**, collected in MeOH.

Compound **2** was obtained as a brown oil, and the molecular formula of C_18_H_22_O_8_ was assigned to this molecule by the anion HRESIMS peak at *m/z* 365.1232 [M − H]^–^ (calcd for C_18_H_21_O_8_, 365.1242). The ^1^H and ^13^C NMR spectroscopic data of **2** (Table 1) resembled those of **1**, and it was determined that the core structure of an oxygenated hydrocarbon-extended dihydroisocoumarin was shared between these molecules. The ^1^H−^1^H COSY correlation between H-2′′ and H-3′′, along with key HMBC correlations from H-3′′ (δ_H_ 2.46) to C-1′′ (δ_C_ 171.8) and C-4′′ (δ_C_ 173.5) (Figure 2), indicated the existence of the linear chain -OCO-CH_2_-CH_2_-CO_2_H in the structure of **2**. Furthermore, according to the same biosynthetic pathway with **1** based on and the key HMBC correlation observed from H-4′ (δ_H_ 4.83) to C-1′′, the planar structure of **2** was established. The absolute configuration for **2** was suggested as being 3*R*,4′*S* based on the biosynthetic logic that it would match that of **1**. The CD spectra of **1** and **2** (Figure 4) are able to be overlapped, with the same Cotton effects observed, which further support the configurational assignment. Accordingly, the resolved structure of compound **2** was afforded the trivial name aspergimarin B.

**FIGURE 5:**
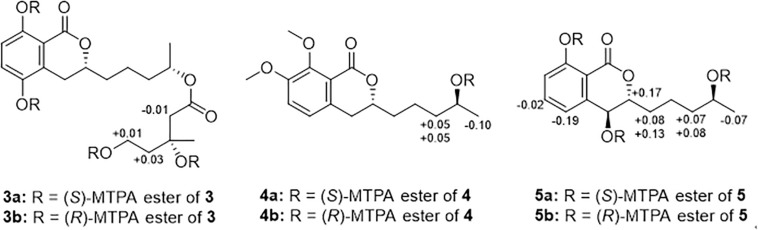
Modified Mosher’s analysis for **3**−**5**. Values shown denote Δδ (δ_S_ −δ_R_) (ppm) for the MTPA esters of **3**−**5**.

Compound **3** was also obtained as a brown oil, and its molecular formula of C_20_H_28_O_8_ was determined by the potassium cation adduct peak in the HRESIMS spectrum at *m/z* 435.1416 [M + K]^+^ (calcd for C_20_H_28_KO_8_, 435.1416). The CD spectrum of **3** (Figure 4) and the ^1^H and ^13^C NMR spectroscopic data (Table 1) were similar to those of both **1** and **2**, indicating that this molecule is another oxygenated hydrocarbon-extended dihydroisocoumarin analog with the same absolute configuration at C-3 and C-4′. It was determined from a ^1^H−^1^H COSY correlation of H-4′′/H-5′′ and the key HMBC correlations from H-2′′ (δ_H_ 2.38) to C-1′′ (δ_C_ 170.4) and C-4′′ (δ_C_ 43.7), as well as from 3′′-OH (δ_H_ 4.53) to C-2′′ (δ_C_ 46.9), C-3′′ (δ_C_ 69.8) and C-6′′ (δ_C_ 27.4) (Figure 2), that there is a different secondary carbon side chain [-OCO-CH_2_-C(CH_3_)(OH)-CH_2_-CH_2_OH] in the molecule of **3** as compared to **2**. Furthermore, the key HMBC correlation observed from H-4′ (δ_H_ 4.83) to C-1′′ allowed for the planar structure of **3** to be completed. Since the biosynthetic logic of **3** with relation to **1** and **2**, together with the matching CD data of these molecules allowed the partial absolute configuration to be assigned as 3*R*,4′*S*, only one stereocenter remained uncertain. Using the modified Mosher’s method (Figure 5) (Gu et al., 2018) the absolute configuration of C-3′′ of **3** was established as being *R*. Therefore, the absolute configuration of **3** was determined to be 3*R*,4′*S*,3′′*R*.

Compound **4** was obtained as a yellow oil, and the molecular formula of this molecule was determined to be C_16_H_22_O_5_ was based on a peak observed in the HRESIMS spectrum at *m/z* 295.1537 [M + H]^+^ (calcd for C_16_H_23_O_5_, 295.1540). The data of **4** from spectroscopic analysis, including UV, IR, and NMR, were extremely similar to those reported for the known compound, penicimarin C (**9**) (Qi et al., 2013). The exceptions noted were determined to be due to the substitution of one methoxy group for a proton at C-7. The placement of the additional methoxy group at C-7 was determined from key HMBC correlations observed from H-5 (δ_H_ 7.06) to C-4 (δ_C_ 32.7), C-7 (δ_C_ 152.2), and C-8a (δ_C_ 119.0), and from 7-*O*-CH_3_ (δ_H_ 3.81) to C-7, together with the ^1^H−^1^H COSY correlation of H-5/H-6 (δ_H_ 7.28) (Figure 2). Accordingly, the planar structure of **4** was unambiguously established as shown. Compared to the CD data of **1** and some previously described values for dihydroisocoumarins (Choukchou-Braham et al., 1994), the observed CD spectrum of **4** (Figure 4) indicated the *R* configuration at C-3. The absolute configuration at the side chain was suggested as 4′*S* to match that of **1** according to biosynthetic logic, and this was confirmed by use of the modified Mosher’s method (Figure 5). Thus, the absolute configuration of **4** was determined to be 3*R*,4′*S*.

Compound **5** was isolated as a colorless oil, and its molecular formula was established as being C_14_H_18_O_5_ according to the associated sodiated molecular ion peak in the HRESIMS spectrum at *m/z* 289.1034 [M + Na]^+^ (calcd for C_14_H_18_NaO_5_, 289.1046). This formula for **5** corresponds to an additional OH with respect to **1**, and the ^1^H and ^13^C NMR data (Table 2) suggested shared carbon skeletons with the substitution of an additional hydroxy group. From the chemical shift differences calculated between **5** and **1**, the additional hydroxy group of **5** was suggested to be at C-4. This assignment was further supported by key HMBC correlations from H-4 (δ_H_ 4.71) to C-5 (δ_C_ 116.3) and from H-5 (δ_H_ 7.03) to C-4 (δ_C_ 67.5), together with the ^1^H−^1^H COSY correlation of H-4/H-3 (δ_H_ 4.47) (Figure 2). The absolute configuration at C-3 was determined to be *R* by the comparison of CD data with **1** and **4** (Figure 4). The absolute configuration of chiral centers at both C-4 and C-4′ in **5** was established as being *S* by use of the modified Mosher’s method (Figure 5). Therefore, the absolute configuration of **5** was determined to be 3*R*,4*S*,4′*S*.

Compound **6** was isolated as a yellow amorphous powder, and its molecular formula was determined to be C_14_H_16_O_5_ by an associated sodiated molecular ion peak in the HRESIMS spectrum at *m/z* 287.0879 [M + Na]^+^ (calcd for C_14_H_16_NaO_5_^+^, 287.0890). The spectroscopic data of **6**, including UV, IR, and NMR, resembled those previously reported for the known molecule penicilloxalone B (**10**) (Ren et al., 2019). From the ^1^H and ^13^C NMR data, it was obvious that the aromatic substitution patterns of **6** and **10** differed, with **6** bearing protons at C-5 and C-6 while **10** has protons at C-6 and C-7. Furthermore, the key HMBC correlations observed from H-5 (δ_H_ 6.63) to C-4 (δ_C_ 31.4), C-7 (δ_C_ 144.4), and C-8a (δ_C_ 108.4) together with the ^1^H−^1^H COSY correlation of H-5/H-6 (δ_H_ 7.00) (Figure 2) corroborated that the two phenolic hydroxy groups of **6** were situated at C-7 and C-8 (δ_C_ 150.0). The absolute configuration of **6** was determined to be 3*R*, the same as for **10**, by comparison of the observed CD spectrum for this molecule with reported data for **10** (Figure 4).

Five additional compounds isolated from *Aspergillus* sp. NBUF87 in the course of this study were determined, by comparison of the spectroscopic and spectrometric data of each with reported values, to be the known isocoumarin derivatives aspergillumarin B (**7**) (Li S. et al., 2012), penicimarin B (**8**) (Qi et al., 2013), penicimarin C (**9**) (Qi et al., 2013), penicilloxalone B (**10**) (Ren et al., 2019), and (*R*)-3-(3-hydroxypropyl)-8-hydroxy-3,4-dihydroisocoumarin (**11**) (Sun et al., 2017). The absolute configurations for **7**-**9** were established to be 3*R*, 4′*S* because the CD spectra (see Supplementary Figures S61, S73), and optical rotation data matched literature values (Li S. et al., 2012; Qi et al., 2013). The absolute configuration of **10** and **11** was also determined to be 3*R* because the optical rotation data matched literature reported values for these molecules (Sun et al., 2017; Ren et al., 2019).

### Effects of Compounds **1**−**11** on Plant Growth of *Arabidopsis thaliana* Columbia-0

**FIGURE 6:**
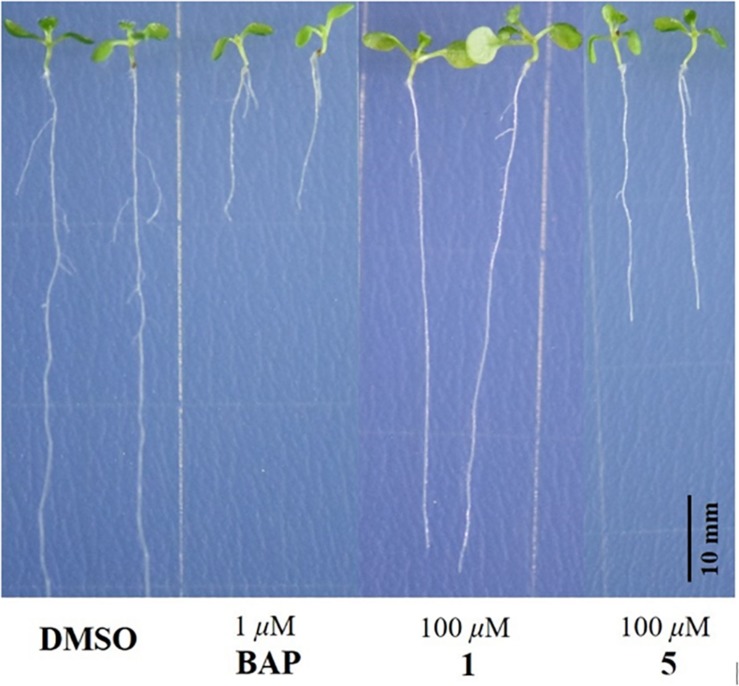
Responses of 100 μM addition of **1** and **5** on the root growth of *Arabidopsis thaliana* Columbia-0 at 10 days after seed germination. DMSO: negative control; BAP: positive control.

The isolated compounds **1**−**11** were subjected to bioassays for testing plant growth response using *A. thaliana* Col-0, a model plant growth organism. At 100 μM, for both **1** and **5,** root growth inhibitory activity was observed against Col-0. Interestingly, while **1** showed only significant inhibitory effect on the lateral root growth of Col-0, **5** caused notable inhibition of both lateral root and primary root growth, as shown in Figure 6. Compounds **2**-**4** and **6**-**11** did not show any obvious activity in the same plant growth response assay at 100 μM.

### Results of Compounds **1**−**11** Against Four Additional Bioassays

All compounds isolated in this study (**1**−**11**) were also tested for their *in vitro* inhibitory activity of AChE in a biochemical assay, and phenotypic tests for cytotoxicity against four human-derived cancer cell lines, namely, CCRF-CEM (acute lymphoblastic leukemia T lymphocyte), MDA-MB-231 (breast cancer), HCT-116 (colon cancer), and AGS (gastric adenocarcinoma), and antibacterial activity toward methicillin-resistant *S. aureus* ATCC43300 and *E. coli* ATCC25922, and antimalarial activity against *P. falciparum* W2. None of these compounds exhibited inhibition of AChE (IC_50_ > 100 μM), cytotoxic activities against any of the cell lines tested (IC_50_ > 50 μM), antibacterial activities toward the two bacteria (MIC > 50 μg/mL), or antimalarial activity against the parasite (EC_50_ > 100 μg/mL).

## Conclusion

In summary, six new dihydroisocoumarin derivatives, aspergimarins A−F (**1**−**6**) were obtained along with five known analogs (**7**−**11**) from the fermentation of a fungus *Aspergillus* sp. NBUF87, isolated from the sponge *Hymeniacidon* sp. collected from the Paracel Islands in the South China Sea. Structurally, compounds **2** and **3** are esters of the C-4′ hydroxy group in the C-3 side chain of **1**, representing relatively rare isocoumarin derivatives according to previous literature reports (Saeed, 2016). In a plant growth response assay using *A. thaliana* Col-0 as a model organism, only **1** and **5** showed inhibitory activity against root growth, while the others were inactive at up to 100 μM. This finding indicates that the substitution pattern of hydroxy groups in these dihydroisocoumarins may play an important role in root growth inhibitory activity, and further studies remain necessary to interrogate this phenomenon. Since none of the isolated compounds, including **1** and **5**, were found to be broadly AChE inhibitors, anticancer agents, antibacterial agents, or antimalarial agents, it is proposed that these root growth inhibitors act through more elaborate signaling pathways. Moreover, **1** and **5** were here as the root growth inhibitors of Col-0, suggesting that they have an important impact in agricultural production.

## Data Availability Statement

The datasets generated for this study can be found in the Cambridge Structural Database https://www.ccdc.cam.ac.uk/structures/accession1919904.

## Author Contributions

All authors conceived the research, analyzed the data, contributed to the study, and approved the final version of the manuscript. LH, LD, XL, NW, WC, XW, and JL performed the experiments. LH wrote the manuscript. LD, CN, JL, XY, and SH read and revised the manuscript.

## Conflict of Interest

The authors declare that the research was conducted in the absence of any commercial or financial relationships that could be construed as a potential conflict of interest.
