# Supplementary material for: New Dihydroisocoumarin Root Growth Inhibitors From the Sponge-Derived Fungus Aspergillus sp. NBUF87
Source: Front Microbiol. 2019 Dec 10;10:2846. doi: 10.3389/fmicb.2019.02846 (PMC6914834; doi:10.3389/fmicb.2019.02846)
Supplement: Supplementary file 1 [file Data_Sheet_1.ZIP › Supplementary material/Supporting Information.pdf]

# Supporting Information

## New Dihydroisocoumarin Root Growth Inhibitors from the Sponge-Derived Fungus *Aspergillus* sp. NBUF87

Liming Huang[1], Lijian Ding[1*], Xiaohui Li[1], Ning Wang[2], Wei Cui[3], Xiao Wang[3], C. Benjamin Naman[1,4], J. Enrico H. Lazaro[5], Xiaojun Yan[1] and Shan He[1*]

[1] Li Dak Sum Yip Yio Chin Kenneth Li Marine Biopharmaceutical Research Center, College of Food and Pharmaceutical Sciences, Ningbo University, Ningbo 315832, People's Republic of China

[2] Institute of Drug Discovery Technology, Ningbo University, Ningbo 315211, People's Republic of China

[3] Zhejiang Provincial Key Laboratory of Pathophysiology, School of Medicine, Ningbo University, Ningbo 315211, People's Republic of China

[4] Center for Marine Biotechnology and Biomedicine, Scripps Institution of Oceanography, University of California, San Diego, La Jolla, California 92093, United States

[5] National Institute of Molecular Biology and Biotechnology, University of the Philippines Diliman, Quezon City 1101, Philippines

*Corresponding authors:

E-Mails: dinglijian@nbu.edu.cn (L.D.); heshan@nbu.edu.cn (S.H.); Tel./Fax: +86-574-87604382.

# Contents


**Figure S1.** $^{1}H$ NMR Spectrum of Aspergimarin A (**1**) in DMSO-$d_6$.

**Figure S2.** $^{13}C$ NMR Spectrum of Aspergimarin A (**1**) in DMSO-$d_6$.

**Figure S3.** DEPT135 Spectrum of Aspergimarin A (**1**) in DMSO-$d_6$.

**Figure S4.** HSQC Spectrum of Aspergimarin A (**1**) in DMSO-$d_6$.

**Figure S5.** HMBC Spectrum of Aspergimarin A (**1**) in DMSO-$d_6$.

**Figure S6.** $^{1}H$-$^{1}H$ COSY Spectrum of Aspergimarin A (**1**) in DMSO-$d_6$.

**Figure S7.** NOESY Spectrum of Aspergimarin A (**1**) in DMSO-$d_6$.

**Figure S8.** HRESIMS of Aspergimarin A (**1**).

**Figure S9.** UV Spectrum of Aspergimarin A (**1**) in MeOH.

**Figure S10.** IR Spectrum of Aspergimarin A (**1**).

**Figure S11.** $^{1}H$ NMR Spectrum of Aspergimarin B (**2**) in DMSO-$d_6$.

**Figure S12.** $^{13}C$ NMR Spectrum of Aspergimarin B (**2**) in DMSO-$d_6$.

**Figure S13.** DEPT135 Spectrum of Aspergimarin B (**2**) in DMSO-$d_6$.

**Figure S14.** HSQC Spectrum of Aspergimarin B (**2**) in DMSO-$d_6$.

**Figure S15.** HMBC Spectrum of Aspergimarin B (**2**) in DMSO-$d_6$.

**Figure S16.** $^{1}H$-$^{1}H$ COSY Spectrum of Aspergimarin B (**2**) in DMSO-$d_6$.

**Figure S17.** NOESY Spectrum of Aspergimarin B (**2**) in DMSO-$d_6$.

**Figure S18.** HRESIMS of Aspergimarin B (**2**).

**Figure S19.** UV Spectrum of Aspergimarin B (**2**) in MeOH.

**Figure S20.** IR Spectrum of Aspergimarin B (**2**).

**Figure S21.** $^{1}H$ NMR Spectrum of Aspergimarin C (**3**) in DMSO-$d_6$.

**Figure S22.** $^{13}C$ NMR Spectrum of Aspergimarin C (**3**) in DMSO-$d_6$.

**Figure S23.** DEPT135 Spectrum of Aspergimarin C (**3**) in DMSO-$d_6$.

**Figure S24.** HSQC Spectrum of Aspergimarin C (**3**) in DMSO-$d_6$.

**Figure S25.** HMBC Spectrum of Aspergimarin C (**3**) in DMSO-$d_6$.

**Figure S26.** $^{1}H$-$^{1}H$ COSY Spectrum of Aspergimarin C (**3**) in DMSO-$d_6$.

**Figure S27.** NOESY Spectrum of Aspergimarin C (**3**) in DMSO-$d_6$.

**Figure S28.** HRESIMS of Aspergimarin C (**3**).

**Figure S29.** UV Spectrum of Aspergimarin C (**3**) in MeOH.

**Figure S30.** IR Spectrum of Aspergimarin C (**3**).

**Figure S31.** $^{1}H$ NMR Spectrum of Aspergimarin D (**4**) in DMSO-$d_6$.

**Figure S32.** $^{13}C$ NMR Spectrum of Aspergimarin D (**4**) in DMSO-$d_6$.

**Figure S33.** DEPT135 Spectrum of Aspergimarin D (**4**) in DMSO-$d_6$.

**Figure S34.** HSQC Spectrum of Aspergimarin D (**4**) in DMSO-$d_6$.

**Figure S35.** HMBC Spectrum of Aspergimarin D (**4**) in DMSO-$d_6$.

**Figure S36.** $^{1}H$-$^{1}H$ COSY Spectrum of Aspergimarin D (**4**) in DMSO-$d_6$.

**Figure S37.** NOESY Spectrum of Aspergimarin D (**4**) in DMSO-$d_6$.

**Figure S38.** HRESIMS of Aspergimarin D (**4**).

**Figure S39.** UV Spectrum of Aspergimarin D (**4**) in MeOH.

**Figure S40.** IR Spectrum of Aspergimarin D (**4**).

**Figure S41.** $^{1}H$ NMR Spectrum of Aspergimarin E (**5**) in $CDCl_3$.

**Figure S42.** $^{13}C$ NMR Spectrum of Aspergimarin E (**5**) in $CDCl_3$.

**Figure S43.** DEPT135 Spectrum of Aspergimarin E (**5**) in $CDCl_3$.

**Figure S44.** HSQC Spectrum of Aspergimarin E (**5**) in $CDCl_3$.

**Figure S45.** HMBC Spectrum of Aspergimarin E (**5**) in $CDCl_3$.

**Figure S46.** $^1H$-$^1H$ COSY Spectrum of Aspergimarin E (**5**) in $CDCl_3$.

**Figure S47.** NOESY Spectrum of Aspergimarin E (**5**) in $CDCl_3$.

**Figure S48.** HRESIMS of Aspergimarin E (**5**).

**Figure S49.** UV Spectrum of Aspergimarin E (**5**) in MeOH.

**Figure S50.** IR Spectrum of Aspergimarin E (**5**).

**Figure S51.** $^1H$ NMR Spectrum of Aspergimarin F (**6**) in DMSO-$d_6$.

**Figure S52.** $^{13}C$ NMR Spectrum of Aspergimarin F (**6**) in DMSO-$d_6$.

**Figure S53.** DEPT135 Spectrum of Aspergimarin F (**6**) in DMSO-$d_6$.

**Figure S54.** HSQC Spectrum of Aspergimarin F (**6**) in DMSO-$d_6$.

**Figure S55.** HMBC Spectrum of Aspergimarin F (**6**) in DMSO-$d_6$.

**Figure S56.** $^1H$-$^1H$ COSY Spectrum of Aspergimarin F (**6**) in DMSO-$d_6$.

**Figure S57.** NOESY Spectrum of Aspergimarin F (**6**) in DMSO-$d_6$.

**Figure S58.** HRESIMS of Aspergimarin F (**6**).

**Figure S59.** UV Spectrum of Aspergimarin F (**6**) in MeOH.

**Figure S60.** IR Spectrum of Aspergimarin F (**6**).

**Figure S61.** $^1H$ NMR Spectrum of **3a** in DMSO-$d_6$ (600 MHz).

**Figure S62.** $^1H$-$^1H$ COSY Spectrum of **3a** in DMSO-$d_6$ (600 MHz).

**Figure S63.** $^1H$ NMR Spectrum of **3b** in DMSO-$d_6$ (600 MHz).

**Figure S64.** $^1H$-$^1H$ COSY Spectrum of **3b** in DMSO-$d_6$ (600 MHz).

**Figure S65.** $^1H$ NMR Spectrum of **4a** in DMSO-$d_6$ (600 MHz).

**Figure S66.** $^1$H-$^1$H COSY Spectrum of **4a** in DMSO-$d_6$ (600 MHz).

**Figure S67.** $^1$H NMR Spectrum of **4b** in DMSO-$d_6$ (600 MHz).

**Figure S68.** $^1$H-$^1$H COSY Spectrum of **4b** in DMSO-$d_6$ (600 MHz).

**Figure S69.** $^1$H NMR Spectrum of **5a** in $CDCl_3$ (600 MHz).

**Figure S70.** $^1$H-$^1$H COSY Spectrum of **5a** in $CDCl_3$ (600 MHz).

**Figure S71.** $^1$H NMR Spectrum of **5b** in $CDCl_3$ (600 MHz).

**Figure S72.** $^1$H-$^1$H COSY Spectrum of **5b** in $CDCl_3$ (600 MHz).

**Figure S73.** Experimental ECD spectra of **7**−**9**, collected in MeOH.

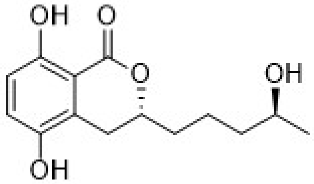


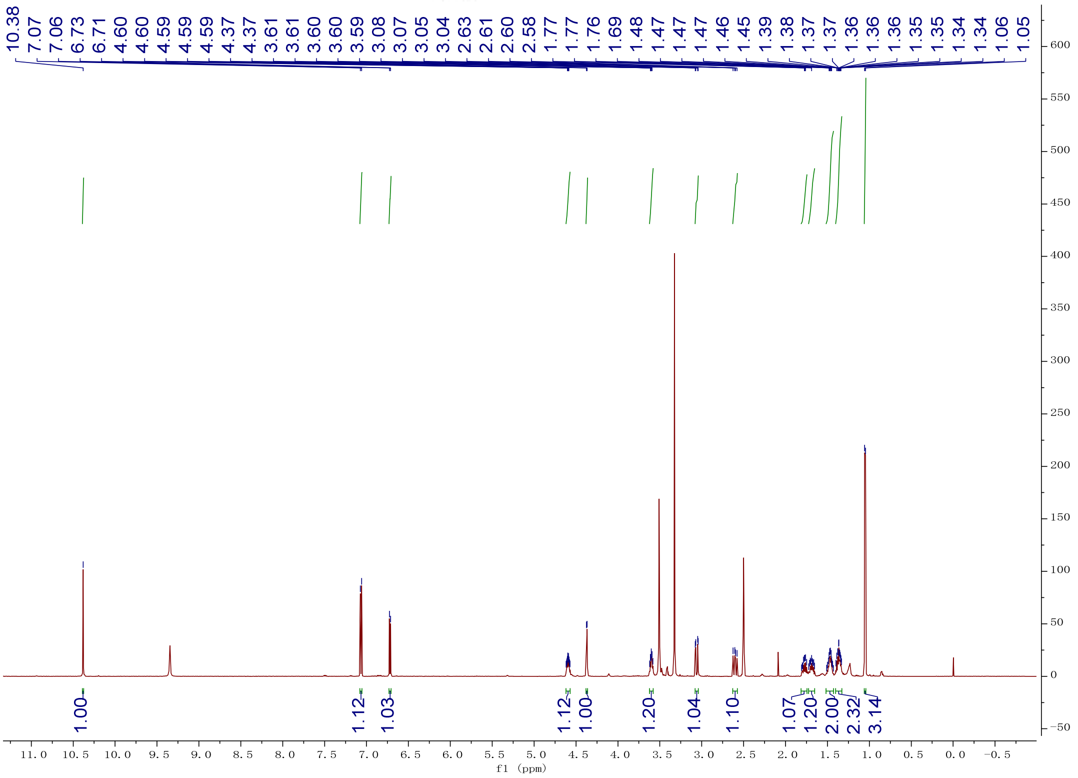


**Figure S1.** $^{1}$H NMR Spectrum of Aspergimarin A (**1**) in DMSO-*d*6.

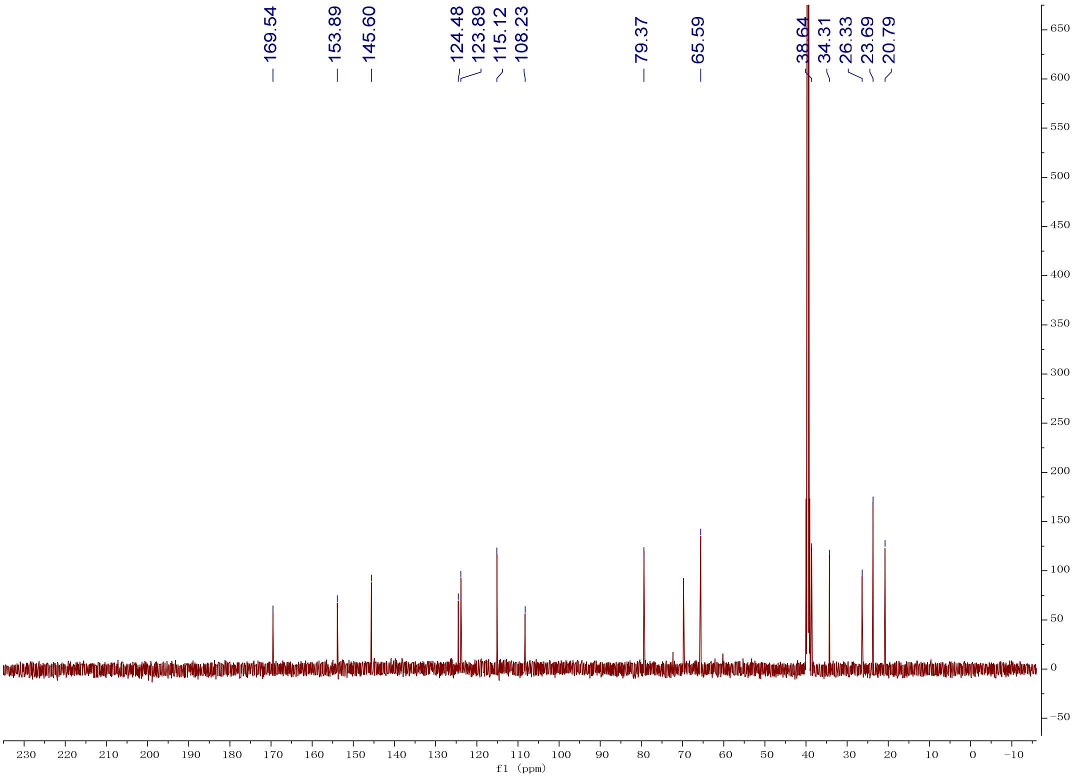


**Figure S2.** $^{13}$C NMR Spectrum of Aspergimarin A (**1**) in DMSO-*d*6.

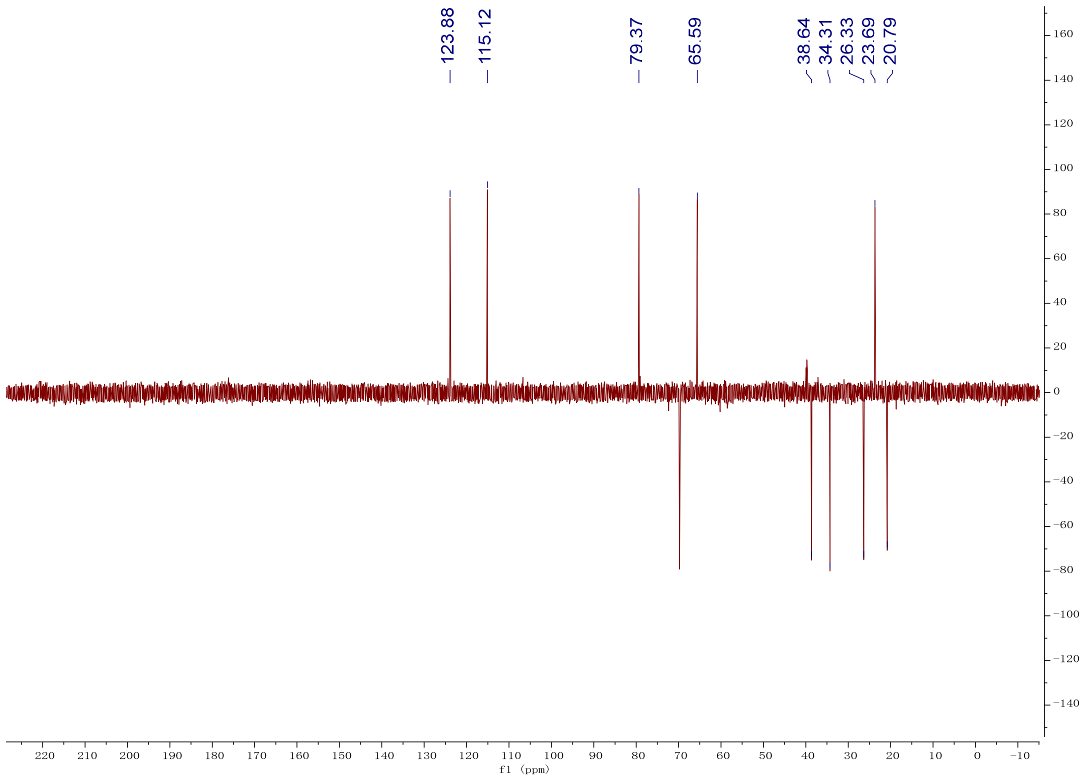


**Figure S3.** DEPT135 Spectrum of Aspergimarin A (**1**) in DMSO-$d_6$.

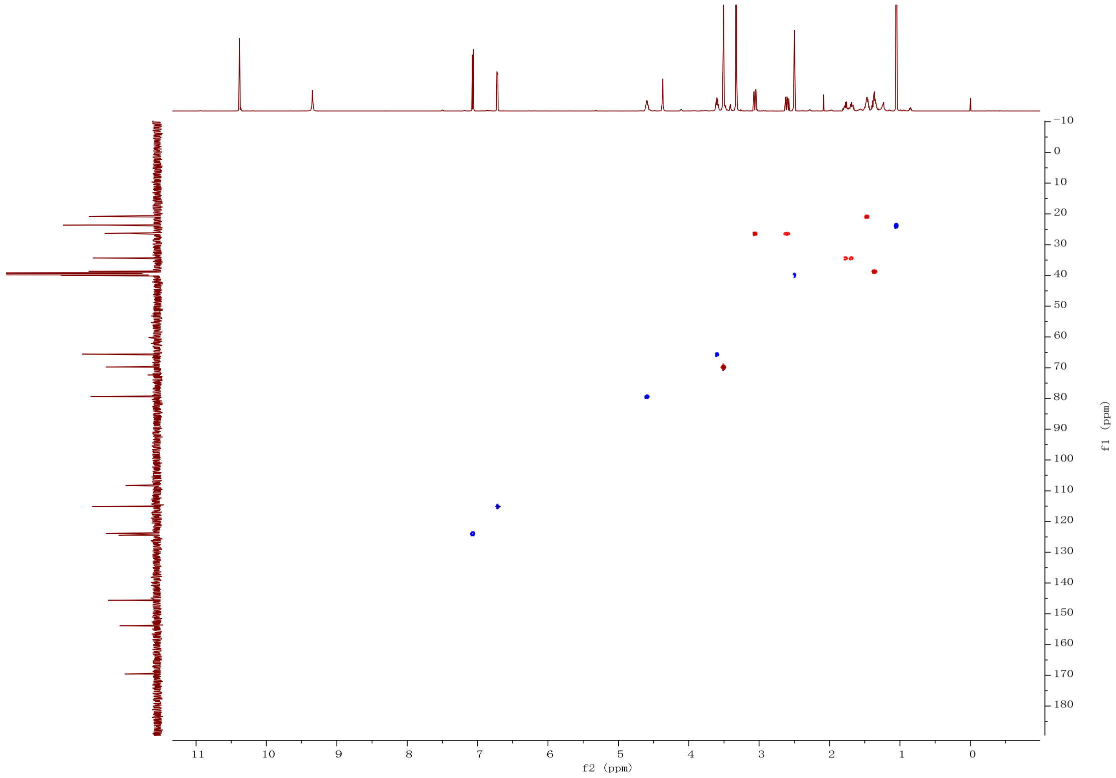


**Figure S4.** HSQC Spectrum of Aspergimarin A (**1**) in DMSO-$d_6$.

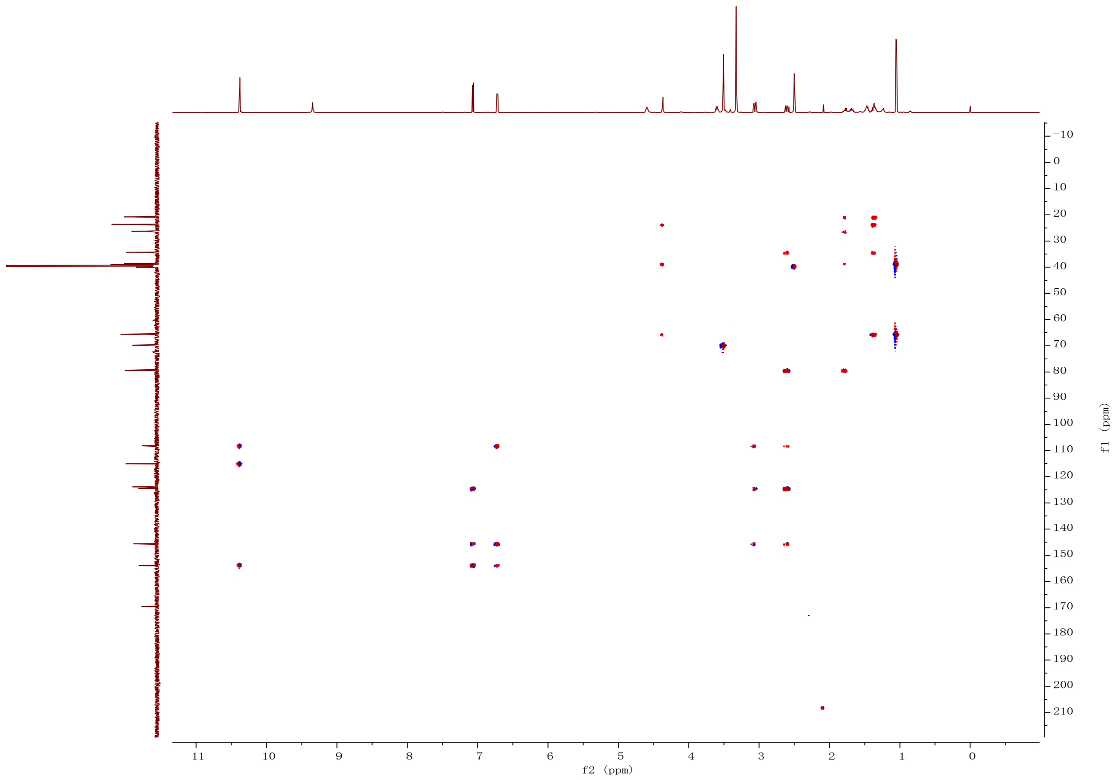


**Figure S5.** HMBC Spectrum of Aspergimarin A (**1**) in DMSO-*d*6.

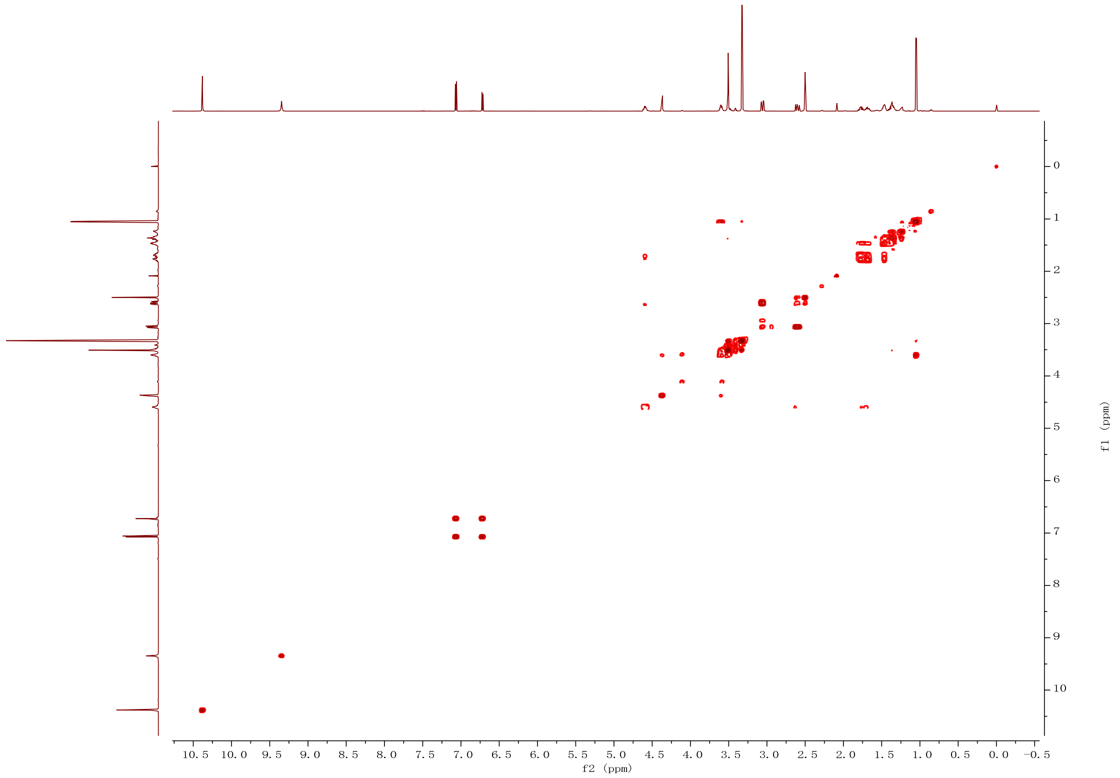


**Figure S6.** $^{1}$H-$^{1}$H COSY Spectrum of Aspergimarin A (**1**) in DMSO-*d*6.

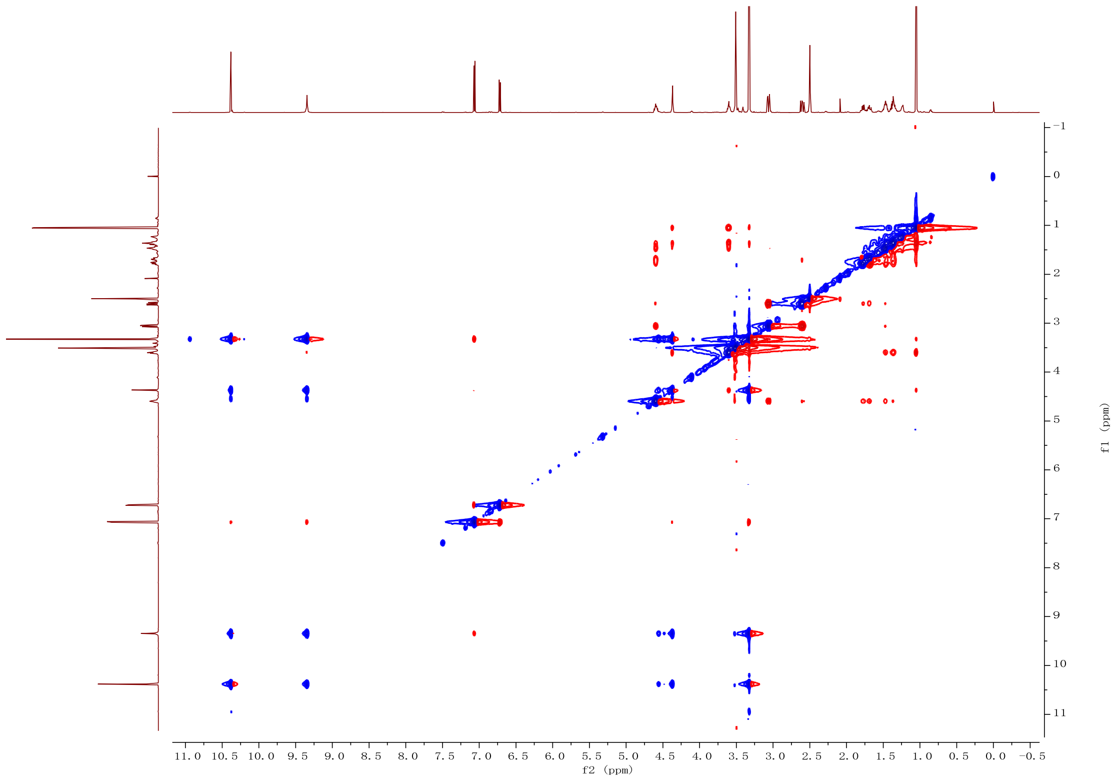


**Figure S7.** NOESY Spectrum of Aspergimarin A (**1**) in DMSO-$d_6$.

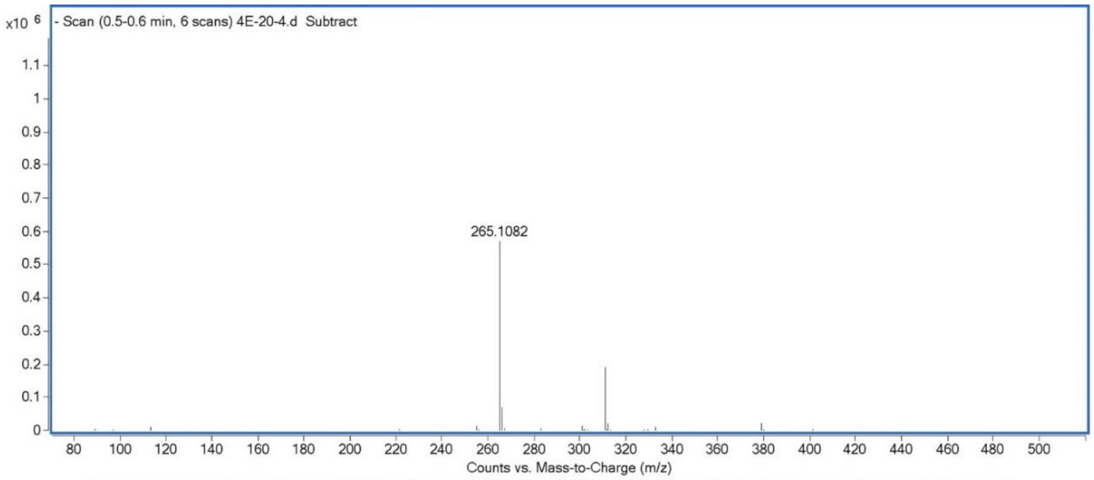


| Formula (M) | Ion Formula | m/z | Calc m/z | Diff (ppm) | DBE |
|---|---|---|---|---|---|
| C14 H18 O5 | C14 H17 O5 | 265.1082 | 265.1081 | -0.2 | 6 |

**Figure S8.** HRESIMS of Aspergimarin A (**1**).

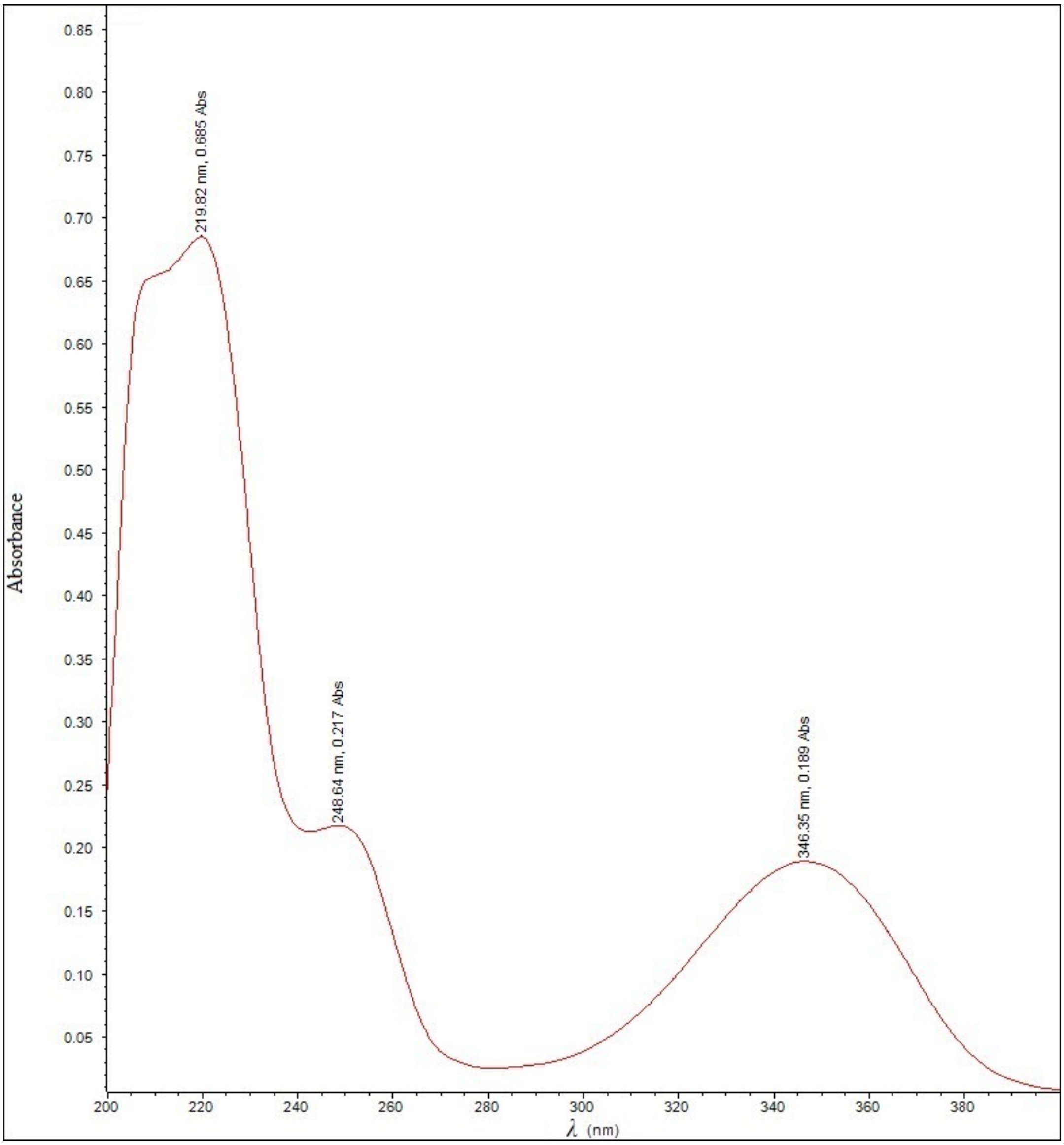


**Figure S9.** UV Spectrum of Aspergimarin A (**1**) in MeOH.

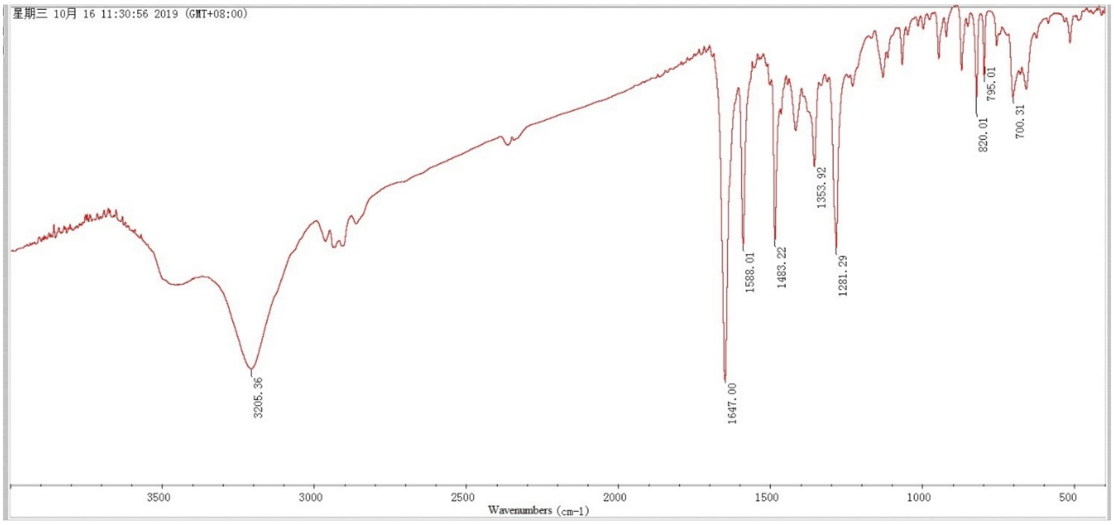


**Figure S10.** IR Spectrum of Aspergimarin A (**1**).

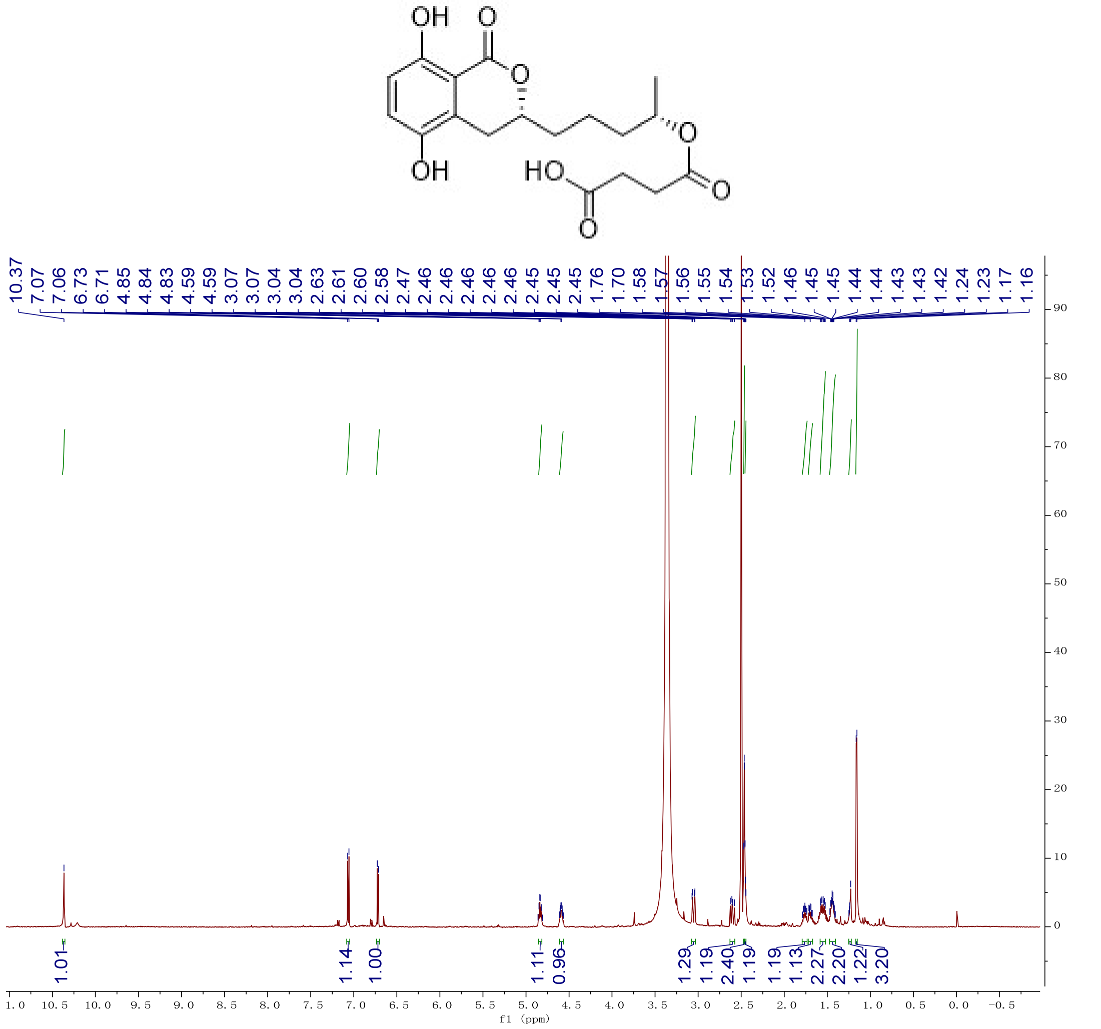


**Figure S11.** $^{1}H$ NMR Spectrum of Aspergimarin B (**2**) in DMSO-$d_6$.

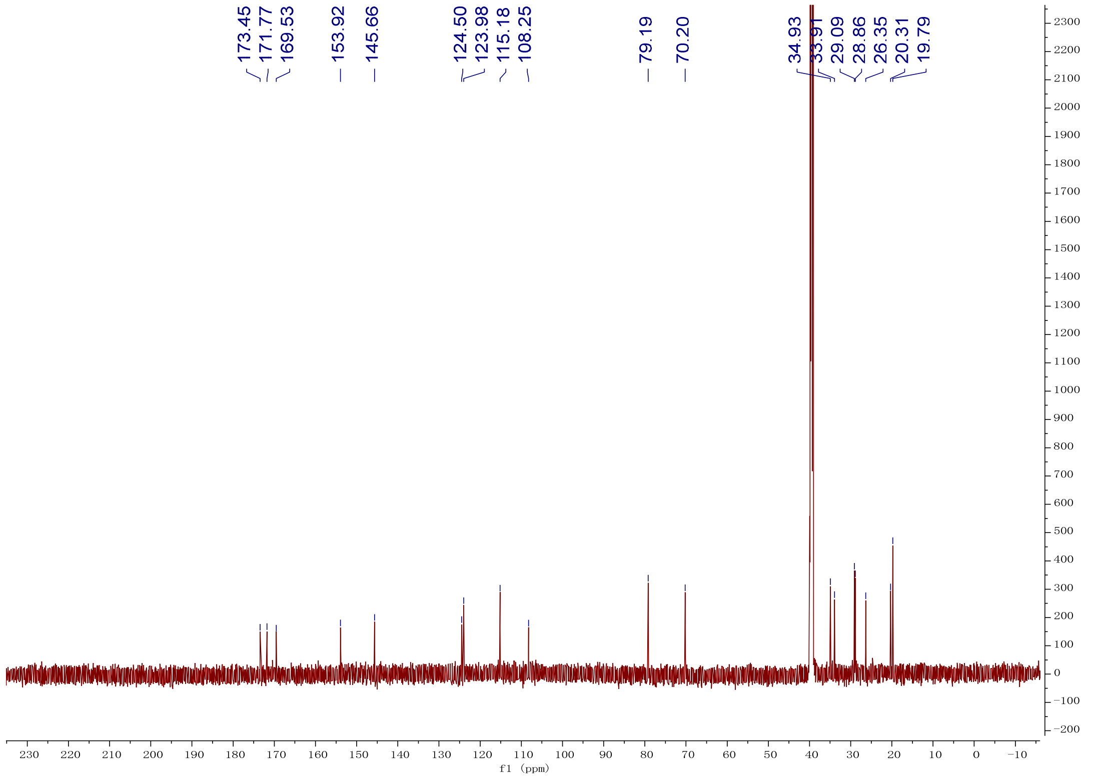


**Figure S12.** $^{13}C$ NMR Spectrum of Aspergimarin B (**2**) in DMSO-$d_6$.

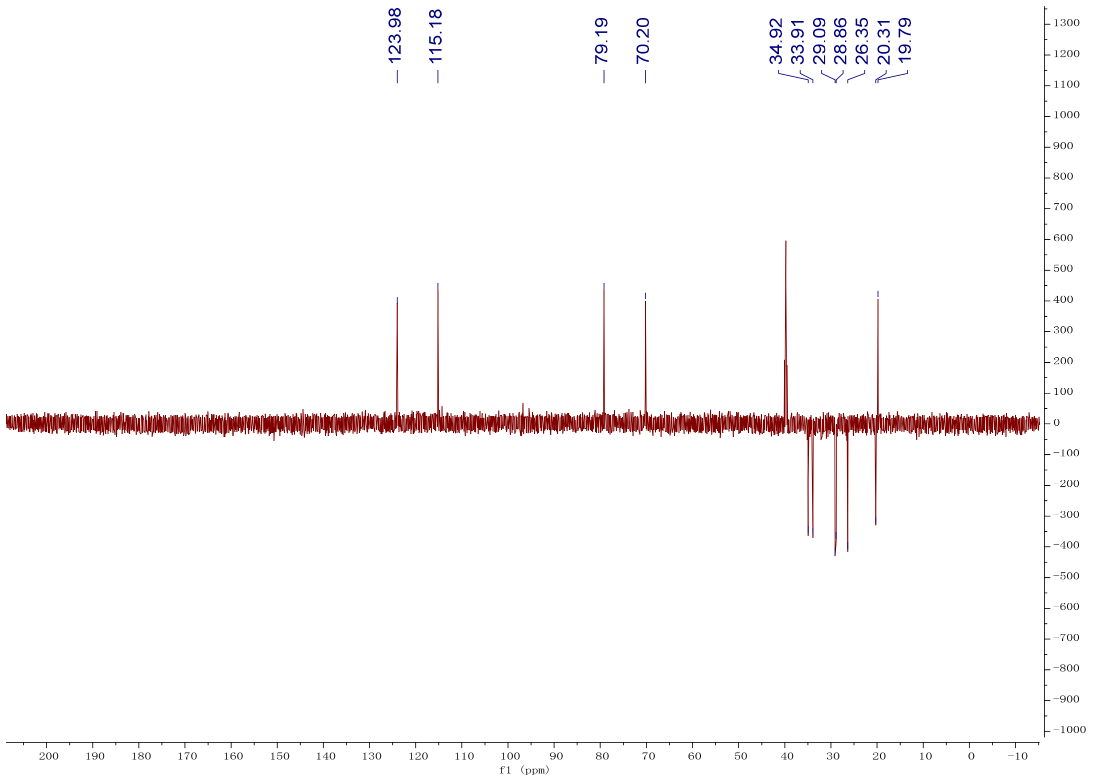


**Figure S13.** DEPT135 Spectrum of Aspergimarin B (**2**) in DMSO-$d_6$.

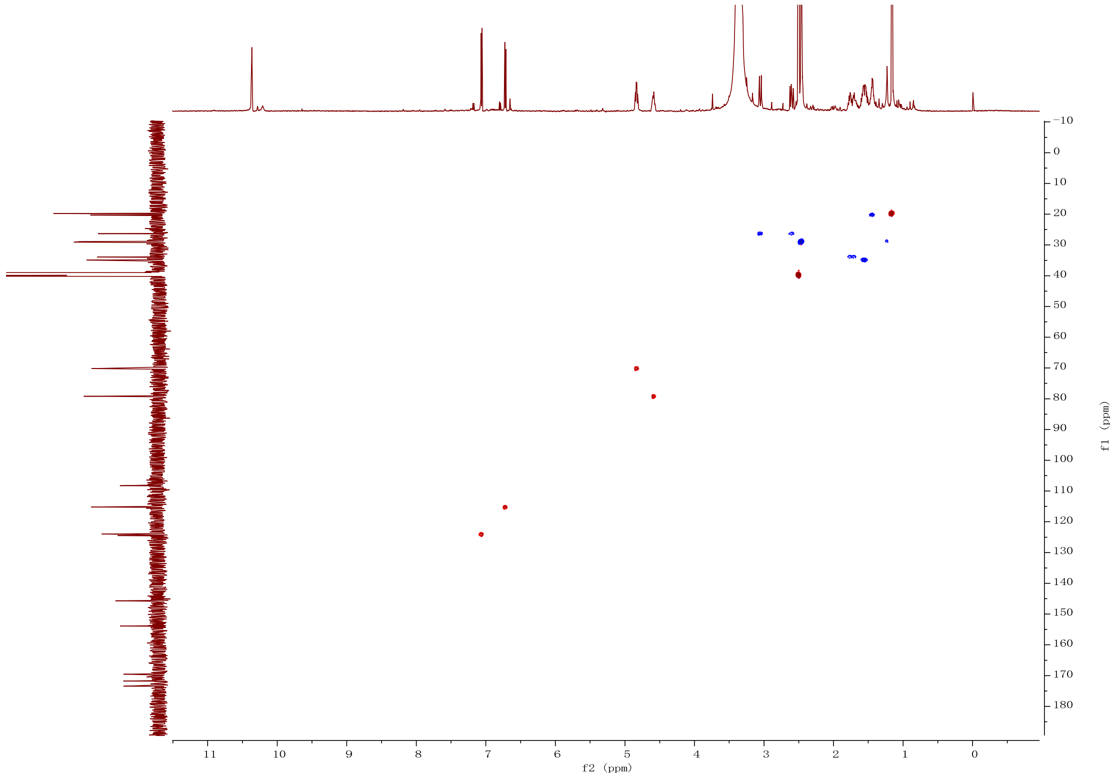


**Figure S14.** HSQC Spectrum of Aspergimarin B (**2**) in DMSO-$d_6$.

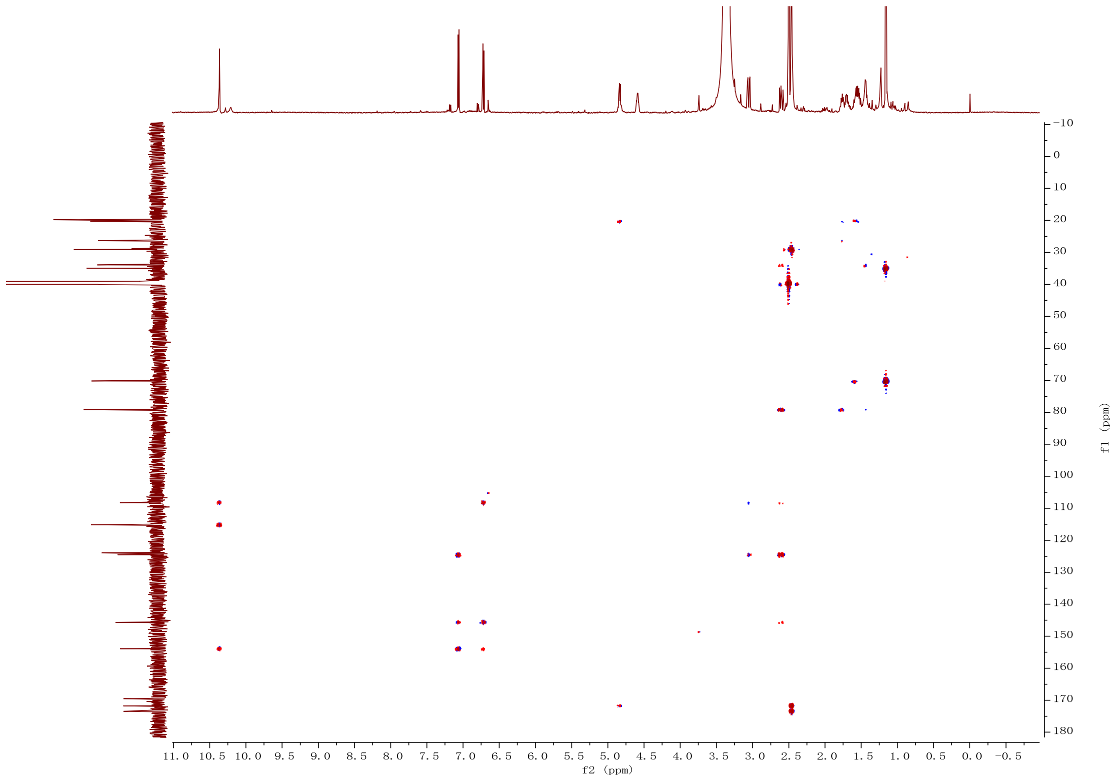


**Figure S15.** HMBC Spectrum of Aspergimarin B (**2**) in DMSO-$d_6$.

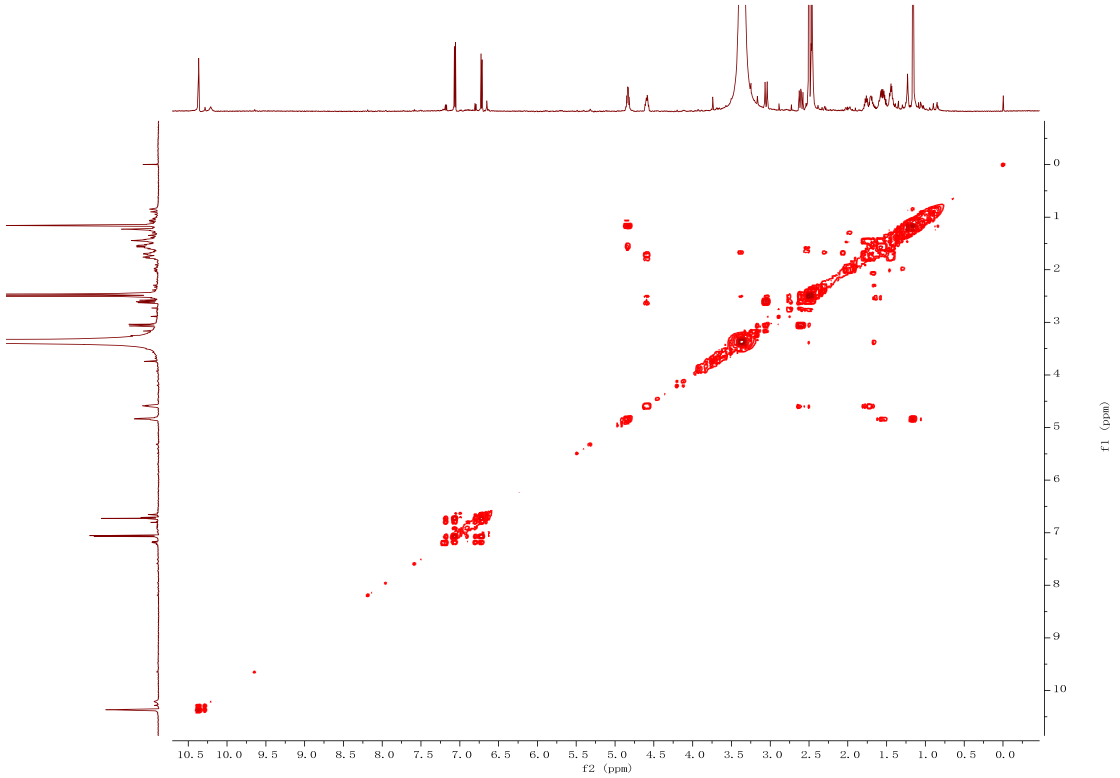


**Figure S16.** $^1$H-$^1$H COSY Spectrum of Aspergimarin B (**2**) in DMSO-$d_6$.

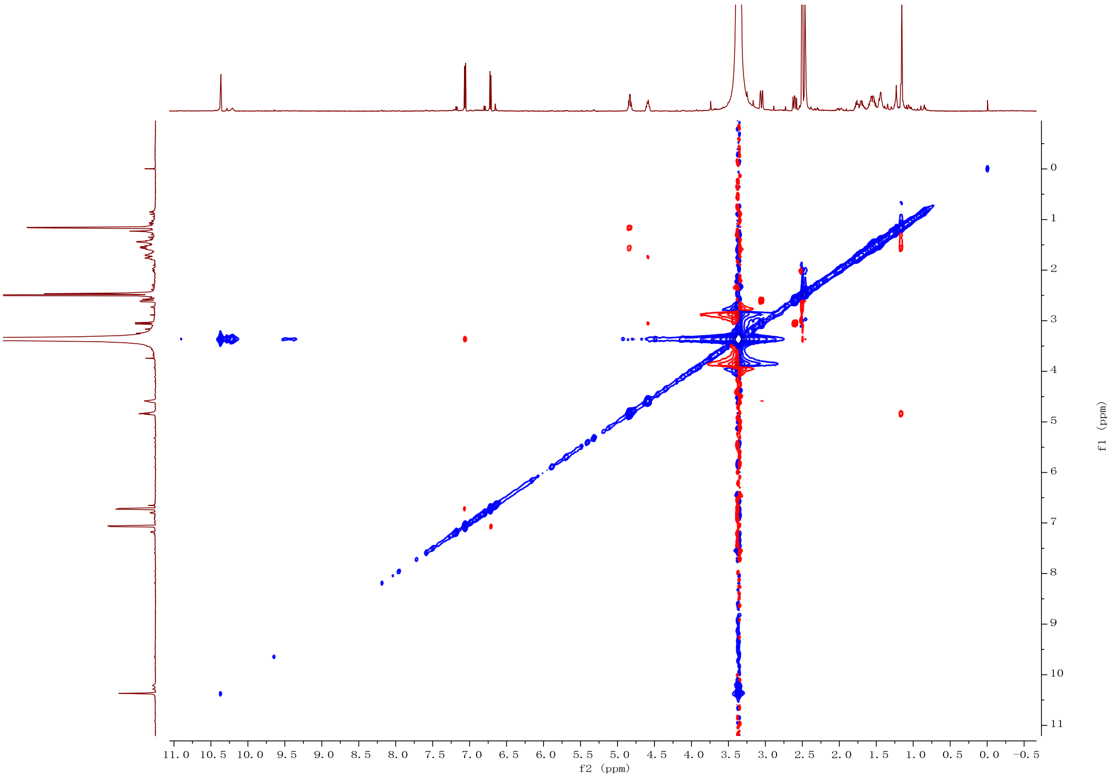


**Figure S17.** NOESY Spectrum of Aspergimarin B (**2**) in DMSO-$d_6$.

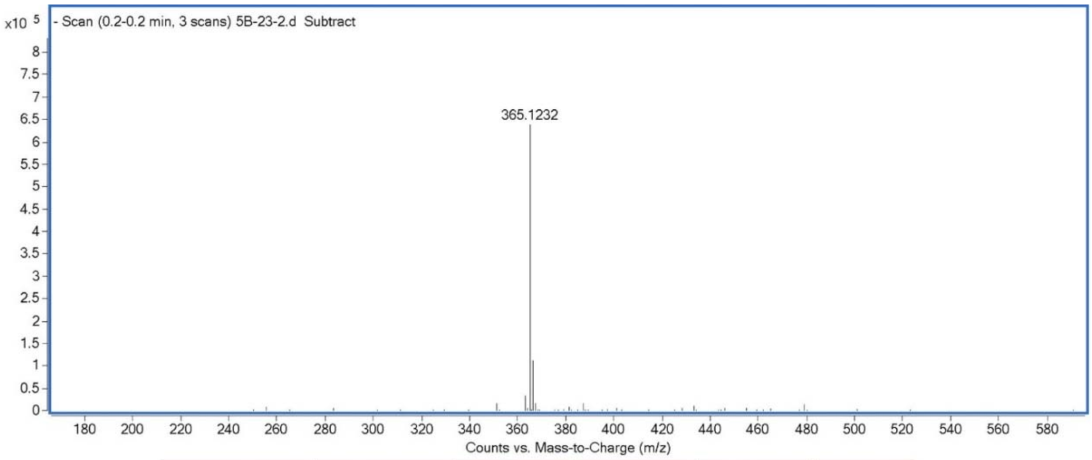


| Formula (M) | Ion Formula | m/z | Calc m/z | Diff (ppm) | DBE |
|---|---|---|---|---|---|
| C18 H22 O8 | C18 H21 O8 | 365.1232 | 365.1242 | 2.71 | 8 |

**Figure S18.** HRESIMS of Aspergimarin B (**2**).

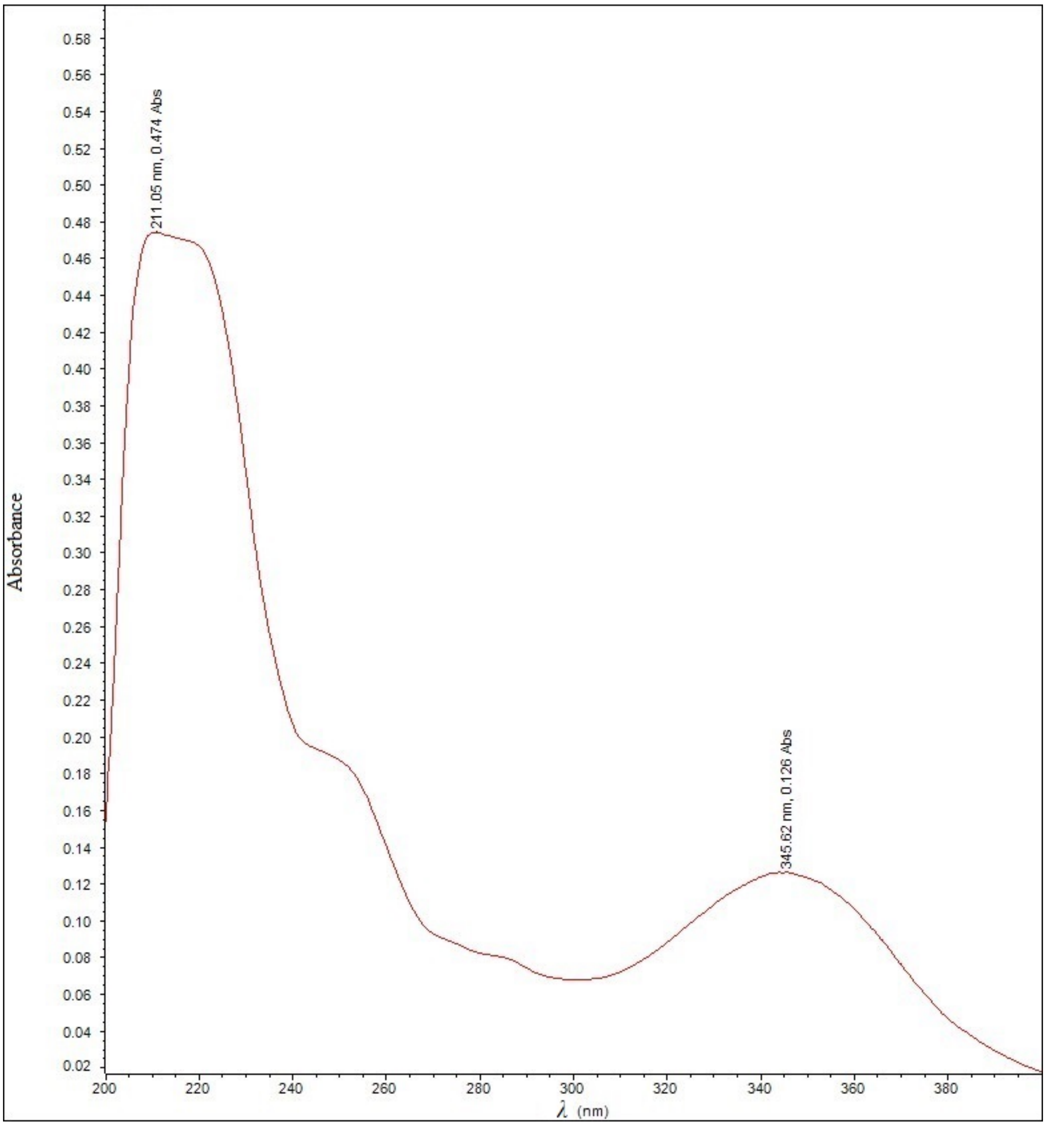


**Figure S19.** UV Spectrum of Aspergimarin B (**2**) in MeOH.

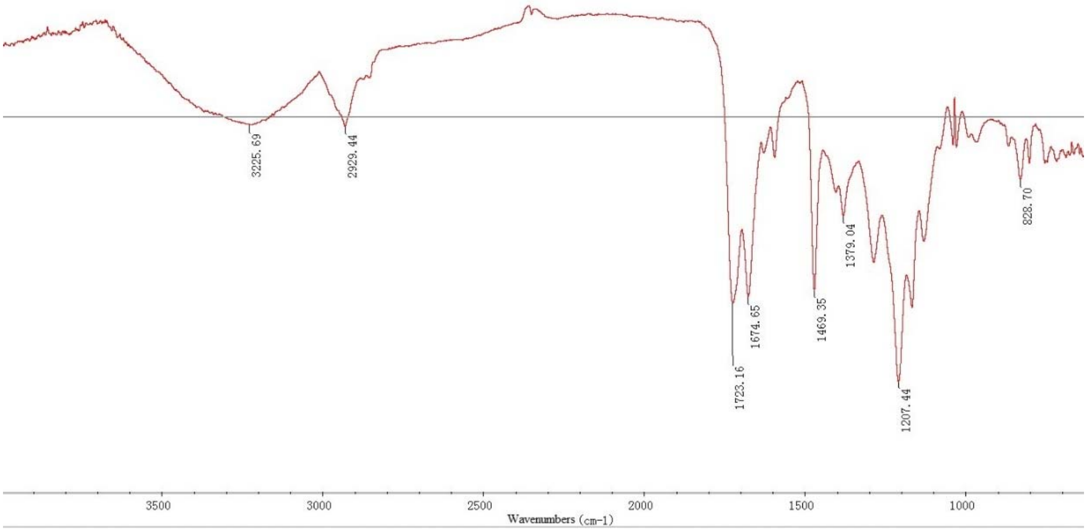


**Figure S20.** IR Spectrum of Aspergimarin B (**2**).

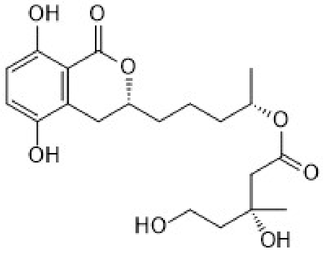

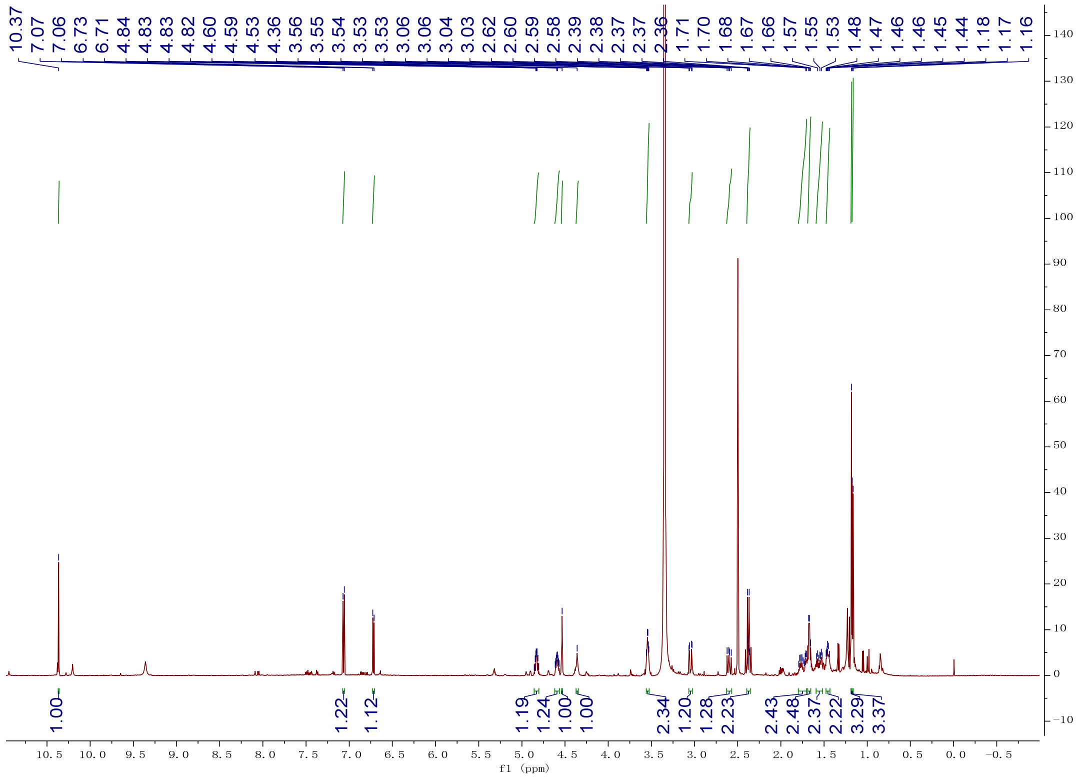

**Figure S21.** $^{1}$H NMR Spectrum of Aspergimarin C (**3**) in DMSO-$d_6$.

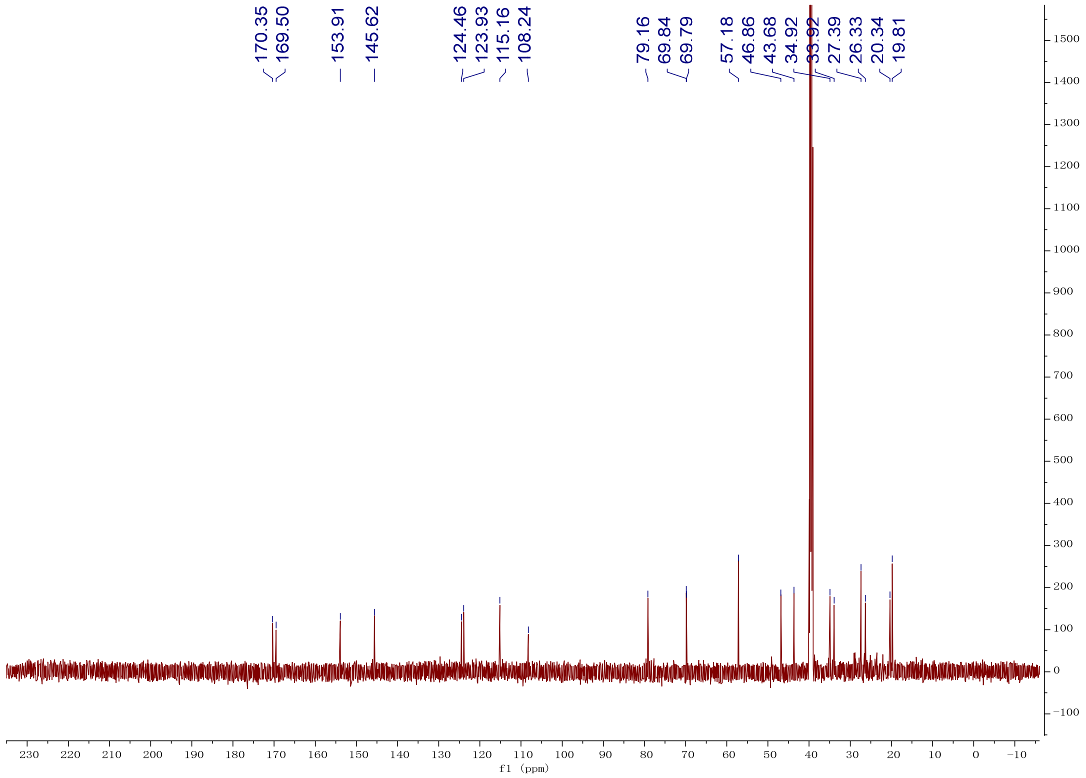

**Figure S22.** $^{13}$C NMR Spectrum of Aspergimarin C (**3**) in DMSO-$d_6$.

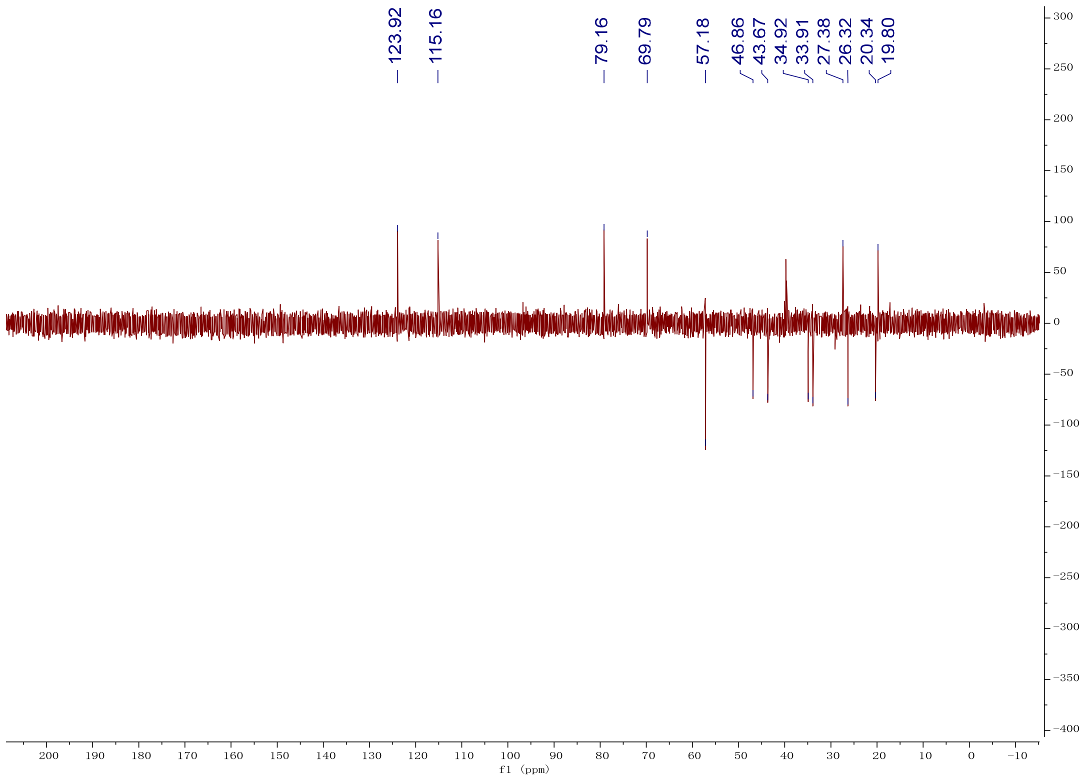


**Figure S23.** DEPT135 Spectrum of Aspergimarin C (**3**) in DMSO-$d_6$.

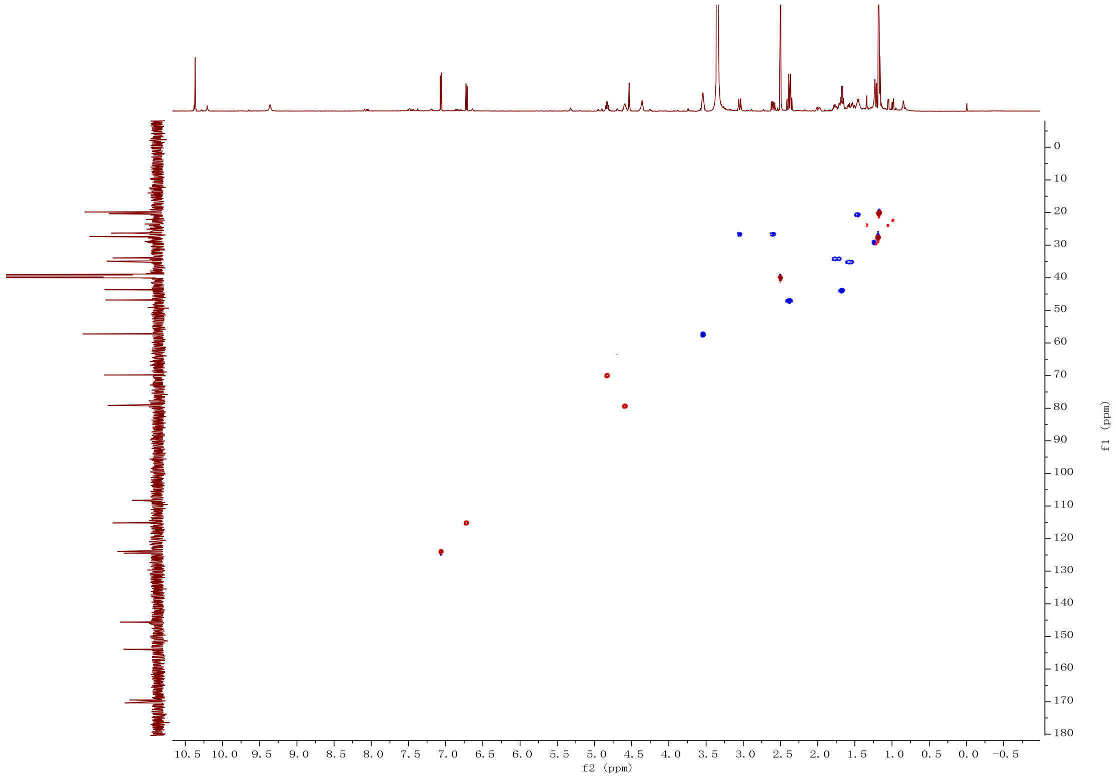


**Figure S24.** HSQC Spectrum of Aspergimarin C (**3**) in DMSO-$d_6$.

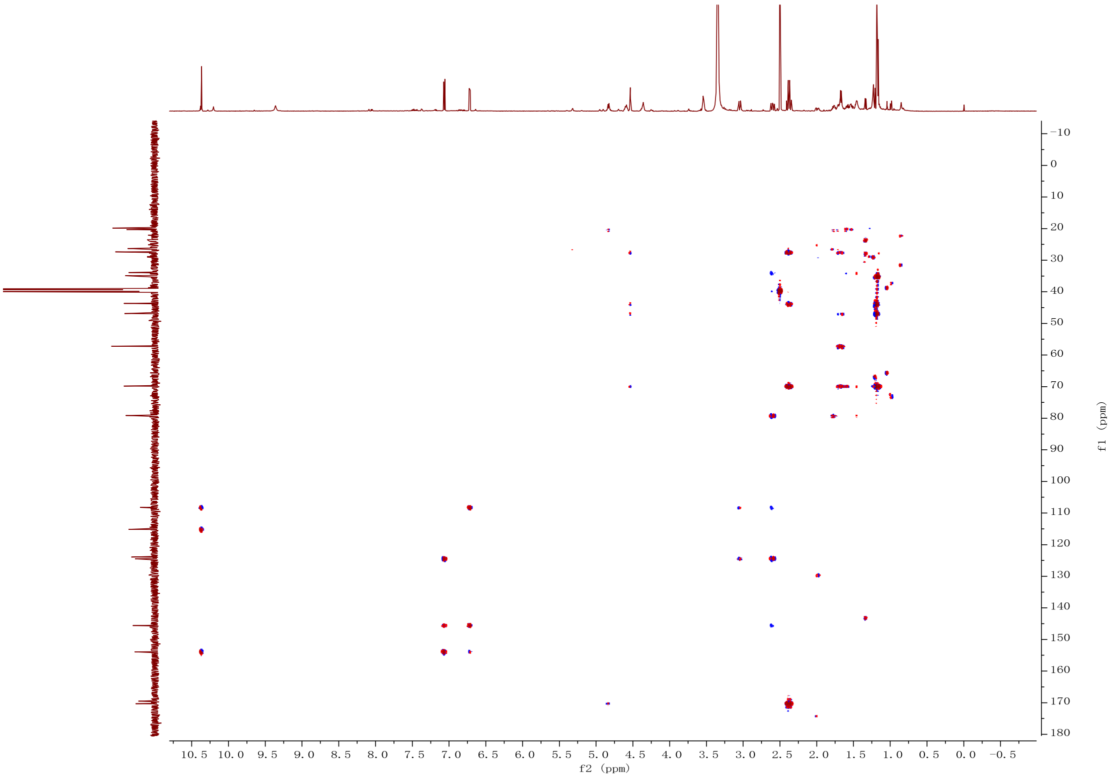


**Figure S25.** HMBC Spectrum of Aspergimarin C (**3**) in DMSO-$d_6$.

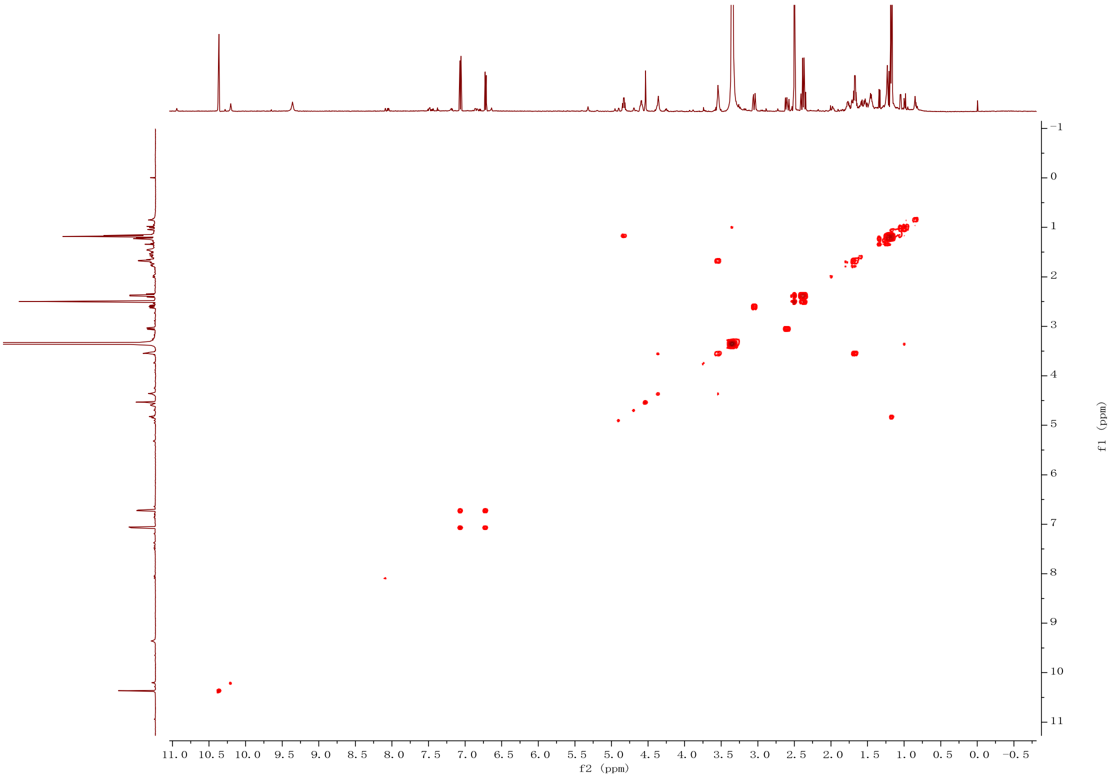


**Figure S26.** $^1$H-$^1$H COSY Spectrum of Aspergimarin C (**3**) in DMSO-$d_6$.

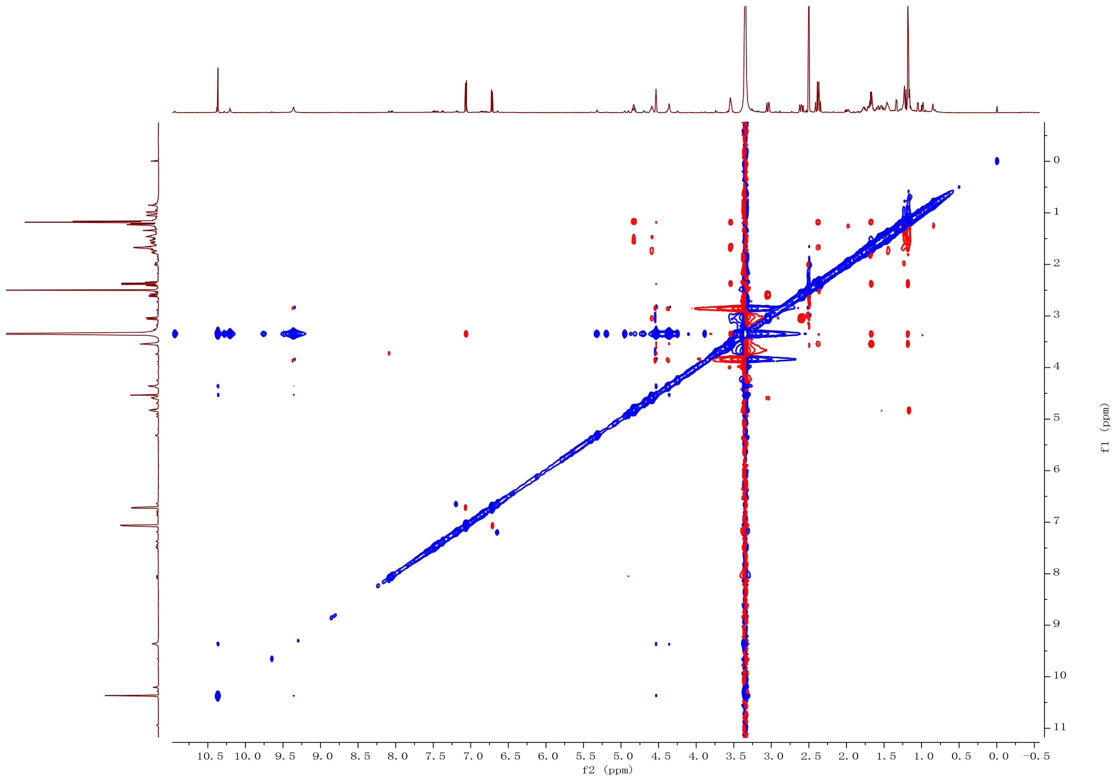


**Figure S27.** NOESY Spectrum of Aspergimarin C (**3**) in DMSO-$d_6$.

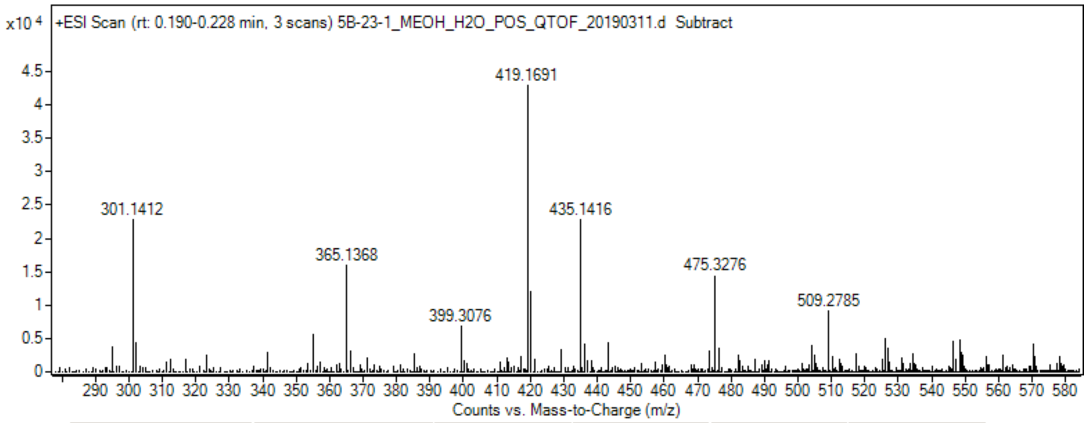


| Formula (M) | Species | m/z | Calc m/z | Diff (ppm) | Score (MFG) |
|---|---|---|---|---|---|
| C20 H28 O8 | (M+Na)+ | 419.1691 | 419.1676 | -3.21 | 89.48 |
| C20 H28 O8 | (M+K)+ | 435.1416 | 435.1415 | -0.69 | 94.09 |

**Figure S28.** HRESIMS of Aspergimarin C (**3**).

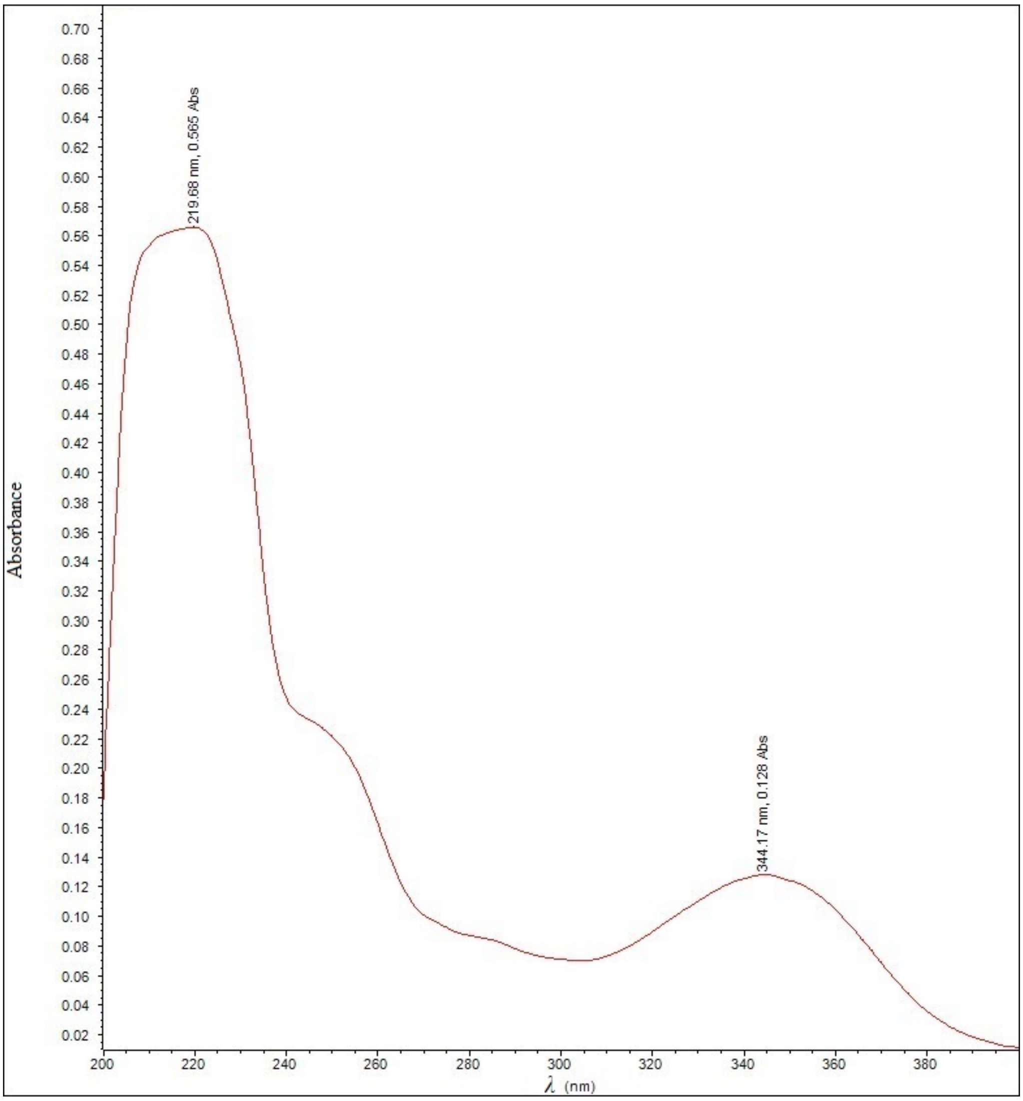


**Figure S29.** UV Spectrum of Aspergimarin C (**3**) in MeOH.

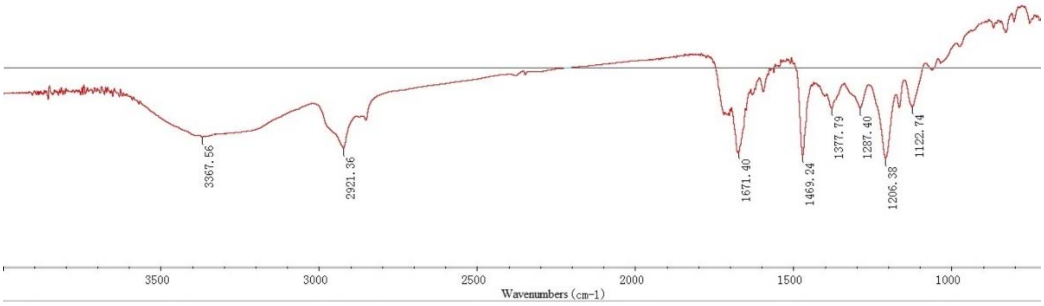


**Figure S30.** IR Spectrum of Aspergimarin C (**3**).

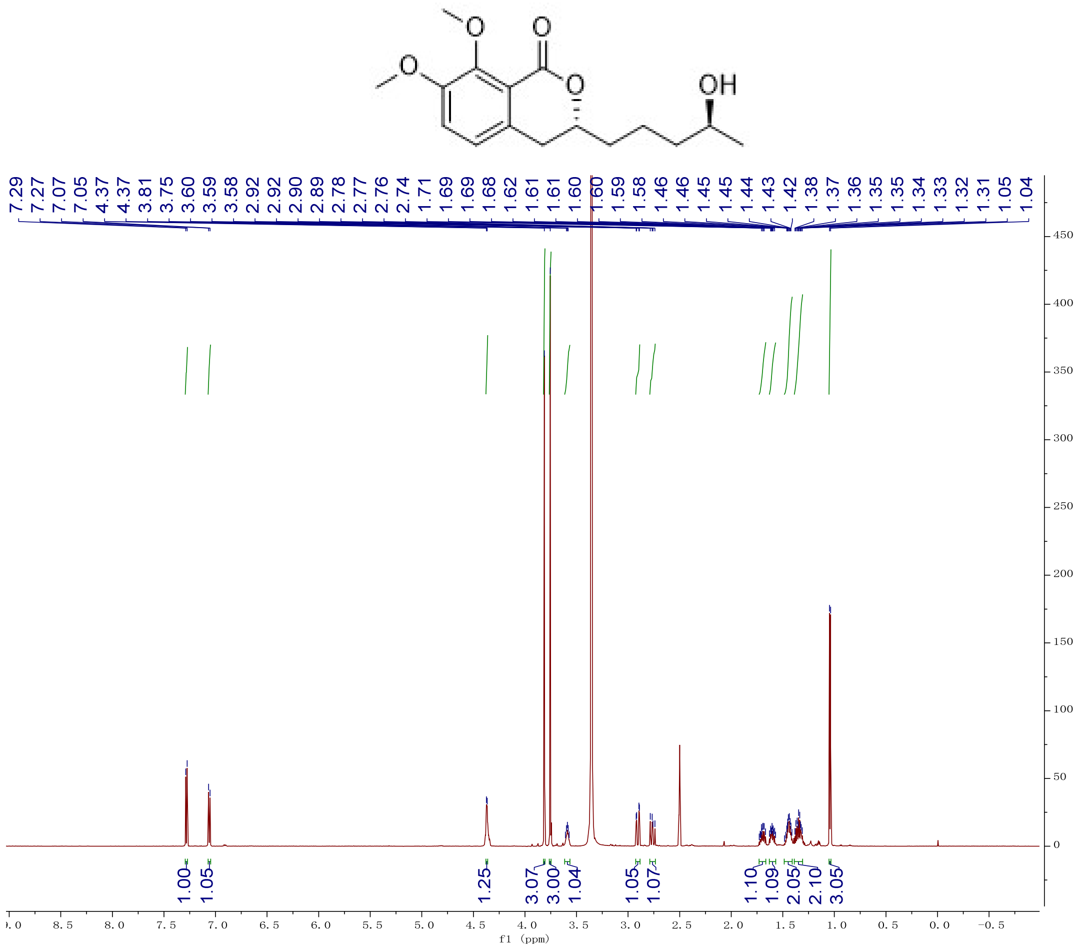


**Figure S31.** $^{1}$H NMR Spectrum of Aspergimarin D (**4**) in DMSO-$d_6$.

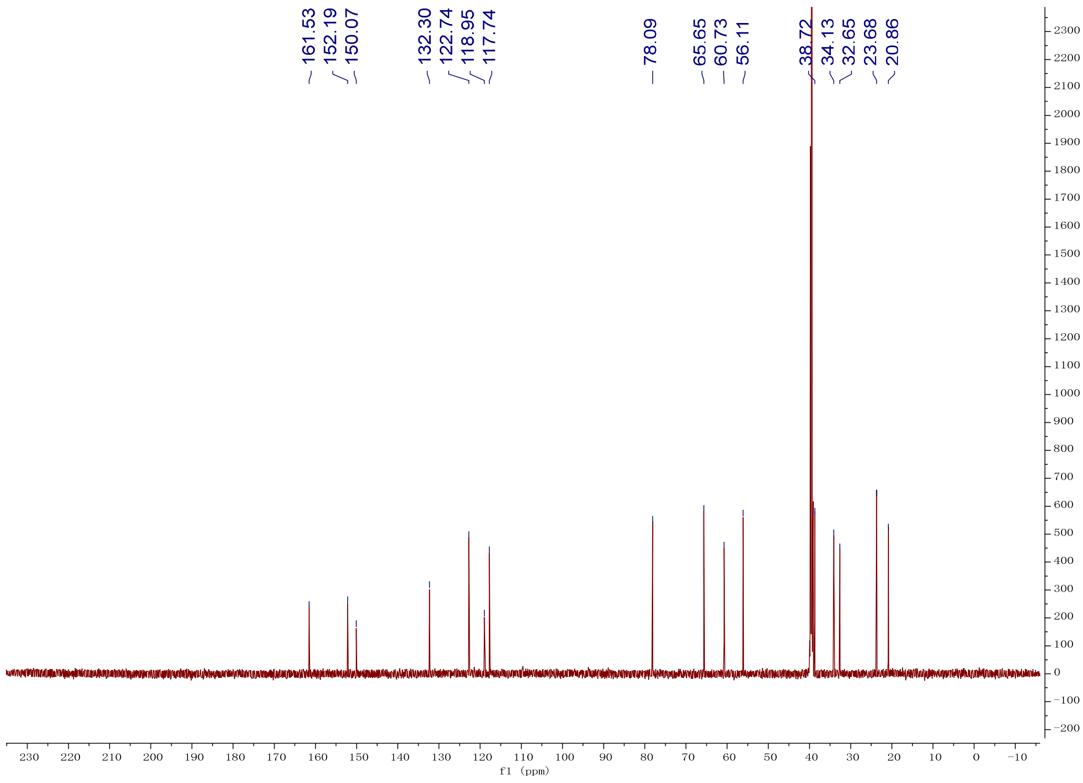


**Figure S32.** $^{13}$C NMR Spectrum of Aspergimarin D (**4**) in DMSO-$d_6$.

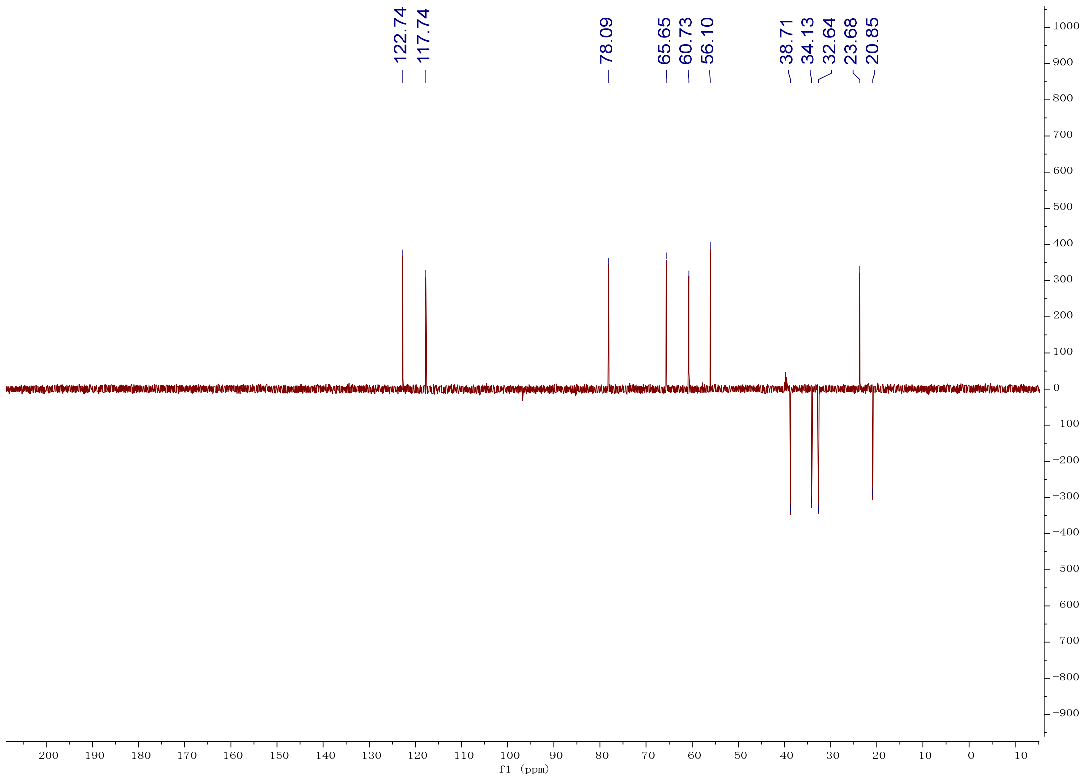


**Figure S33.** DEPT135 Spectrum of Aspergimarin D (**4**) in DMSO-$d_6$.

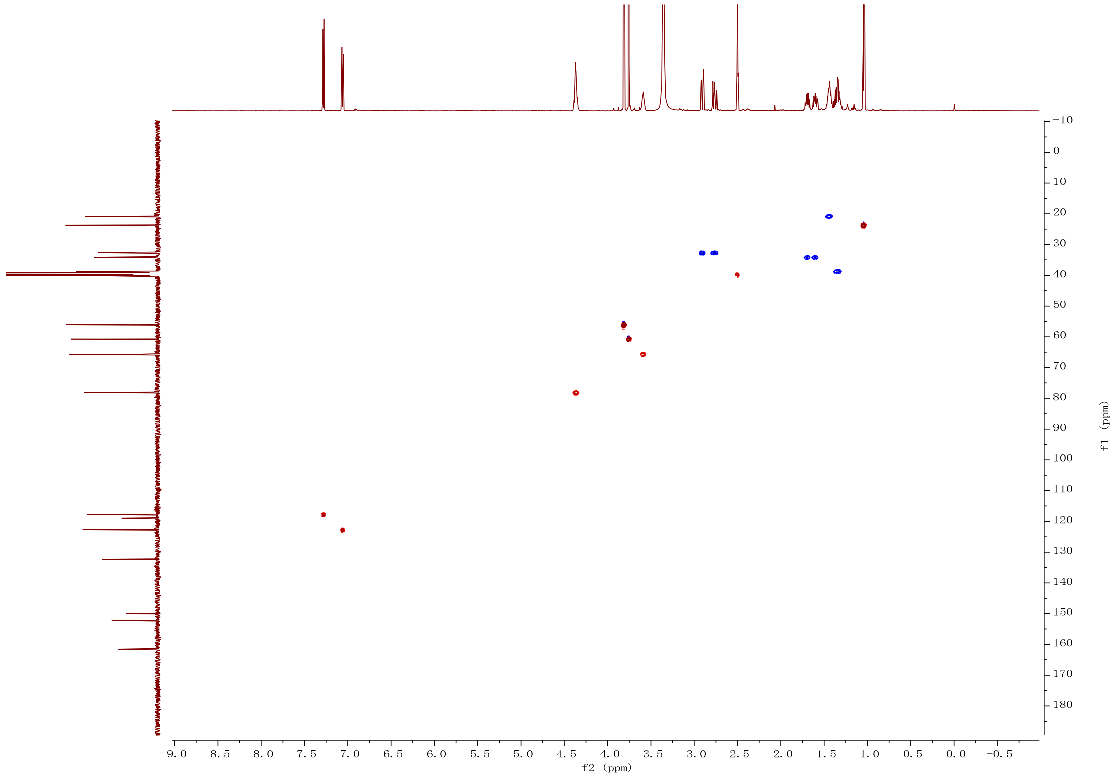


**Figure S34.** HSQC Spectrum of Aspergimarin D (**4**) in DMSO-$d_6$.

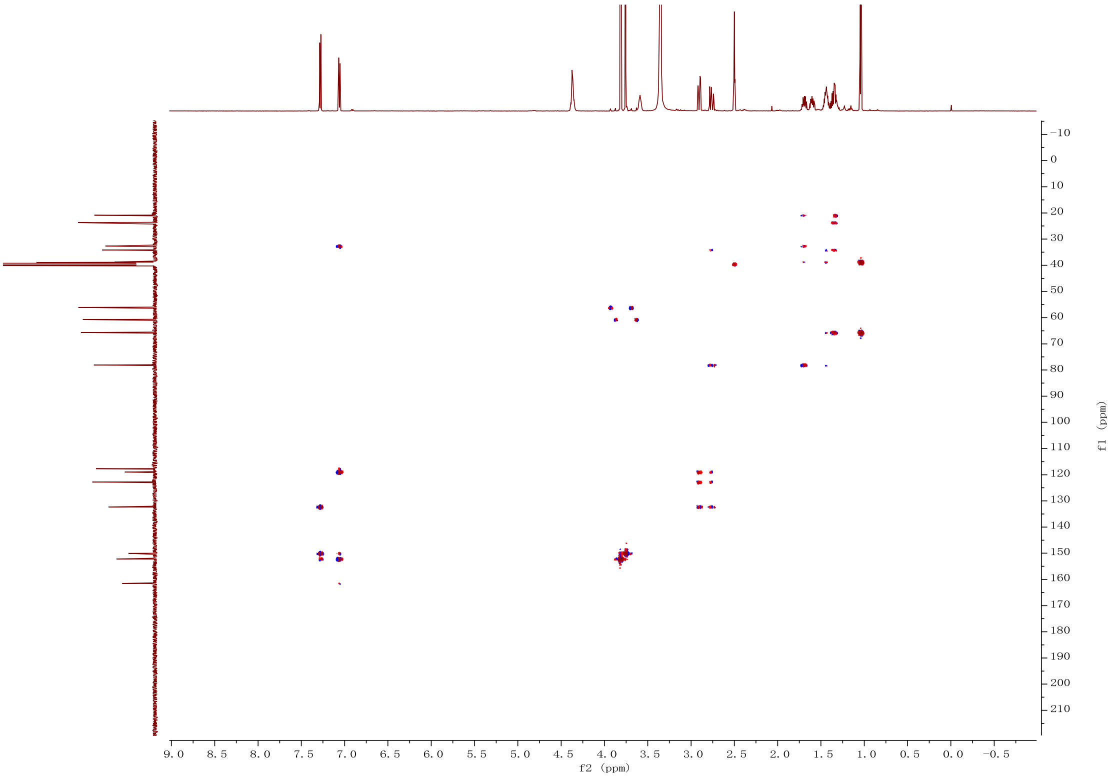


**Figure S35.** HMBC Spectrum of Aspergimarin D (**4**) in DMSO-$d_6$.

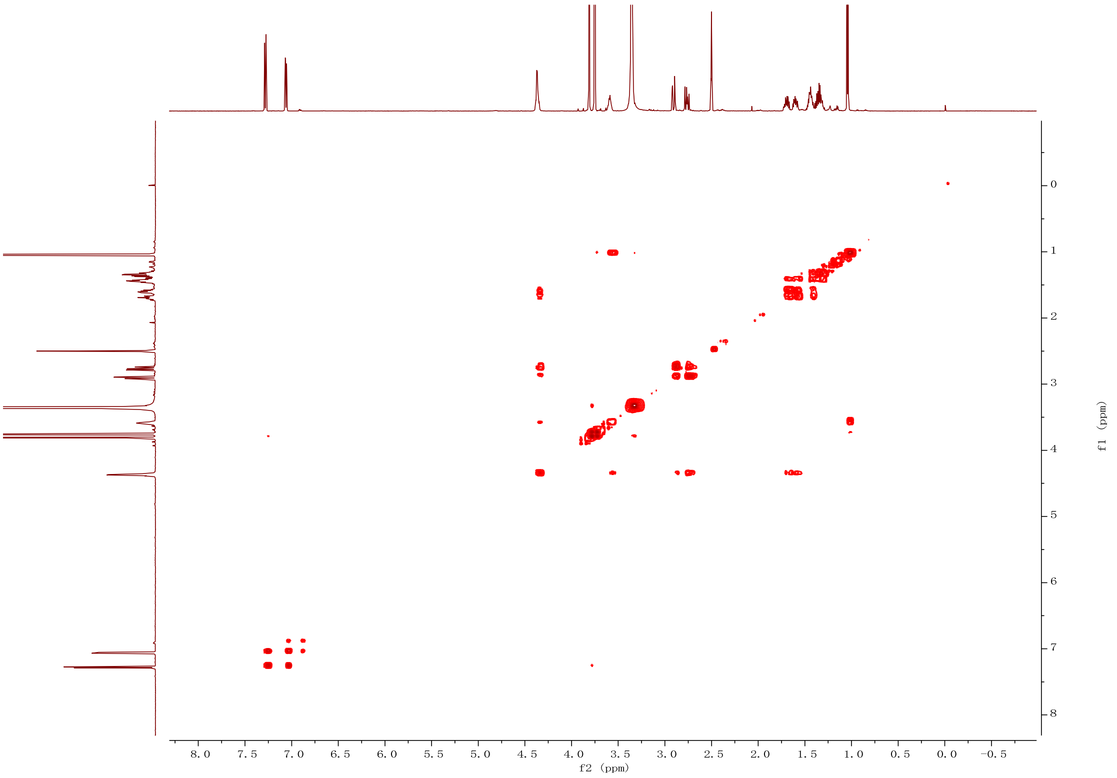


**Figure S36.** $^1$H-$^1$H COSY Spectrum of Aspergimarin D (**4**) in DMSO-$d_6$.

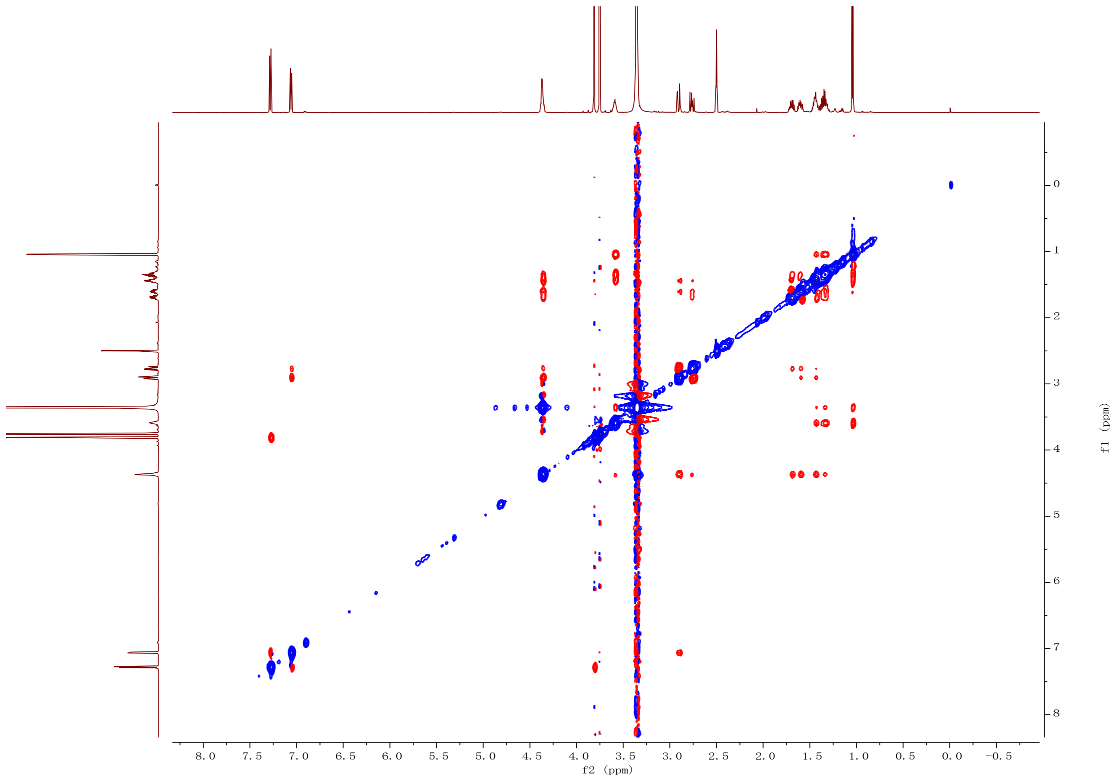


**Figure S37.** NOESY Spectrum of Aspergimarin D (**4**) in DMSO-$d_6$.

## State Key Laboratory of Organometallic Chemistry
## Shanghai Institute of Organic Chemistry
## Chinese Academy of Sciences
## ESI High Resolution MS Date Report

| | |
|---|---|
| **Data Filename** | 5A-15-1.d |
| **Sample Name** | 5A-15-1 |
| **User Name** | |
| **Acquired Time** | 10/17/2018 2:02:16 PM |
| **Instrument** | |

Agilent Technologies 6224 TOF LC/MS

**User Spectra**

| Fragmentor Voltage | Collision Energy | Ionization Mode |
|---|---|---|
| 100 | 0 | ESI |

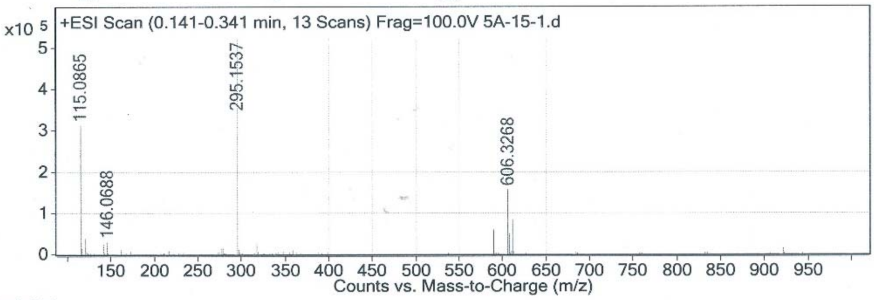


**Peak List**

| *m/z* | z | Abund | Formula | Ion |
|---|---|---|---|---|
| 115.0865 | | 312196.4 | | |
| 121.0509 | | 37839.7 | | |
| 146.0688 | | 29637.4 | | |
| 295.1537 | 1 | 521218.6 | C16 H23 O5 | (M+H)+ |
| 295.2072 | | 32514.3 | | |
| 296.1574 | 1 | 70395 | C16 H23 O5 | (M+H)+ |
| 589.3006 | | 59807.7 | | |
| 606.3268 | 1 | 156166.6 | | |
| 607.3307 | 1 | 48996.6 | | |
| 611.2824 | 1 | 83952.7 | | |

**Formula Calculator Results**

| IonFormula | Measured Mass | Tgt Mass | Diff (ppm) | Score |
|---|---|---|---|---|
| C16 H23 O5 | 295.1537 | 295.154 | 1.09 | 95.04 |
| C14 H21 N3 O4 | 295.1537 | 295.1527 | -3.48 | 93.05 |

--- End Of Report ---

Agilent Technologies Page 1 of 1 Printed at: 2:07 PM on: 10/17/2018

**Figure S38.** HRESIMS of Aspergimarin D (**4**).

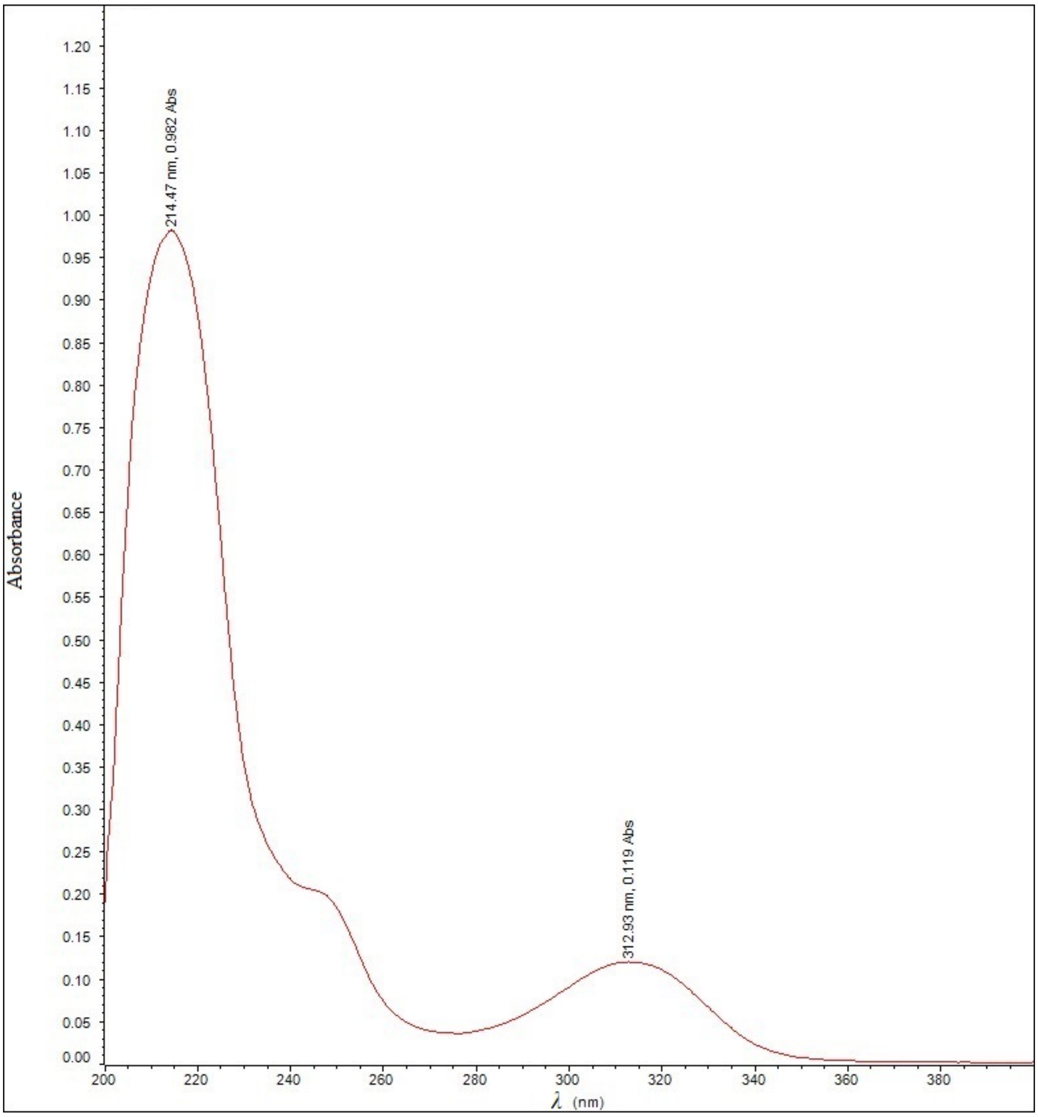


**Figure S39.** UV Spectrum of Aspergimarin D (**4**) in MeOH.

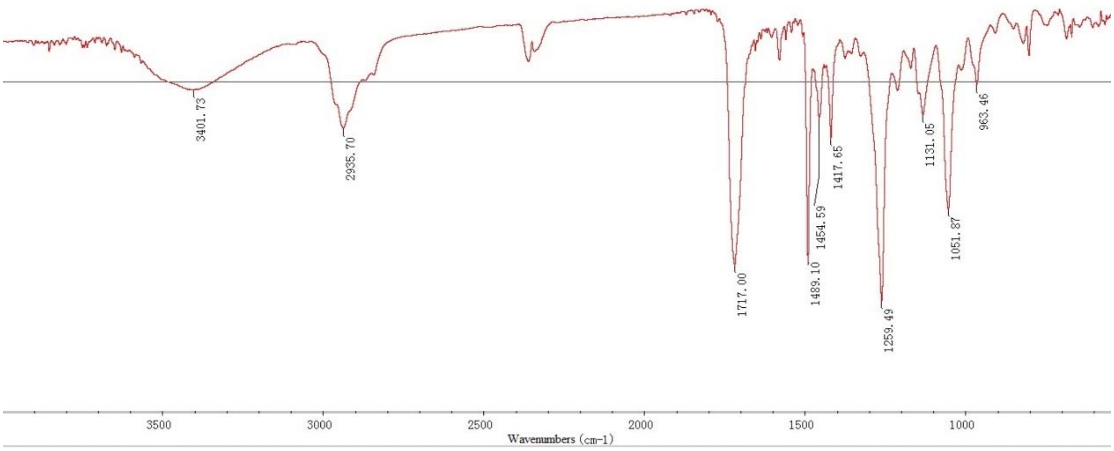


**Figure S40.** IR Spectrum of Aspergimarin D (**4**).

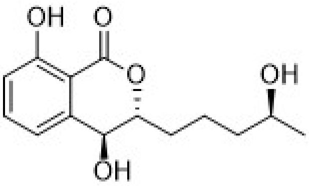

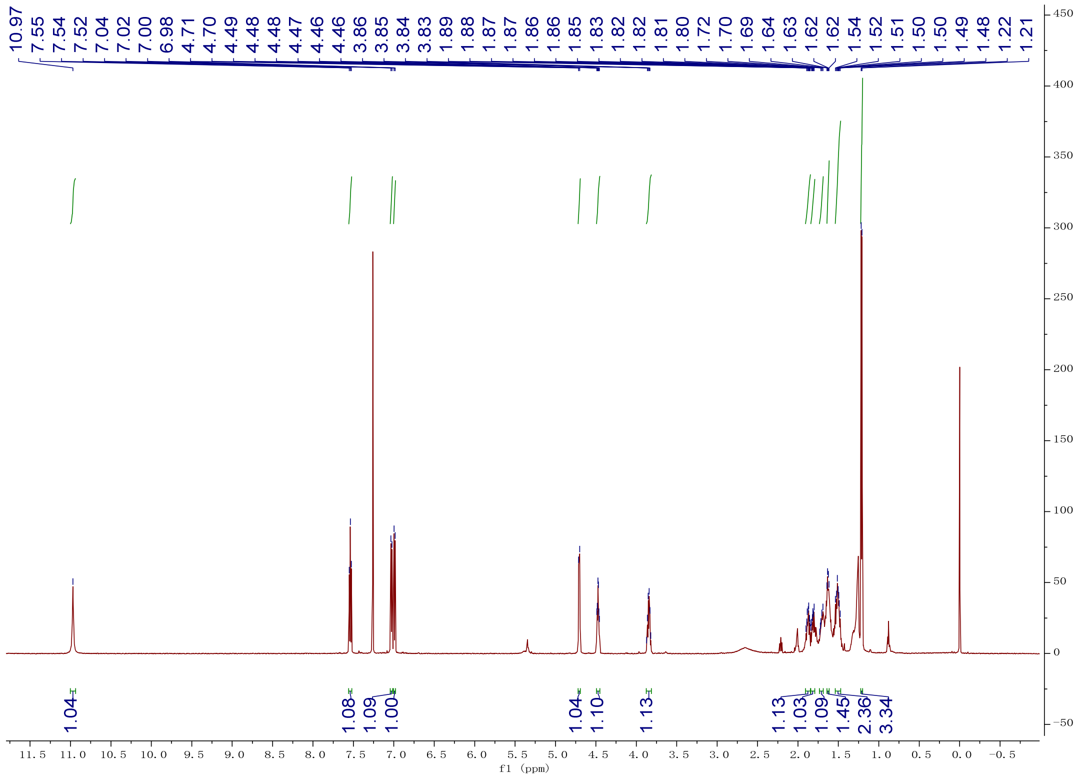

**Figure S41.** $^{1}$H NMR Spectrum of Aspergimarin E (**5**) in $CDCl_3$.

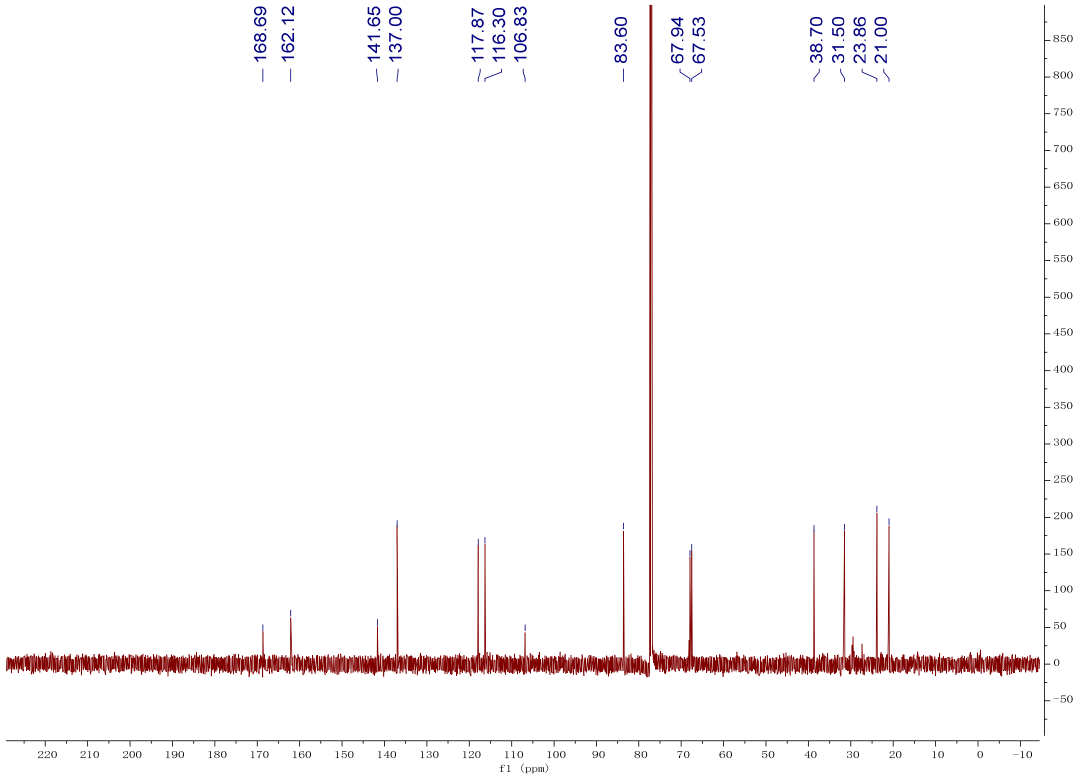

**Figure S42.** $^{13}$C NMR Spectrum of Aspergimarin E (**5**) in $CDCl_3$.

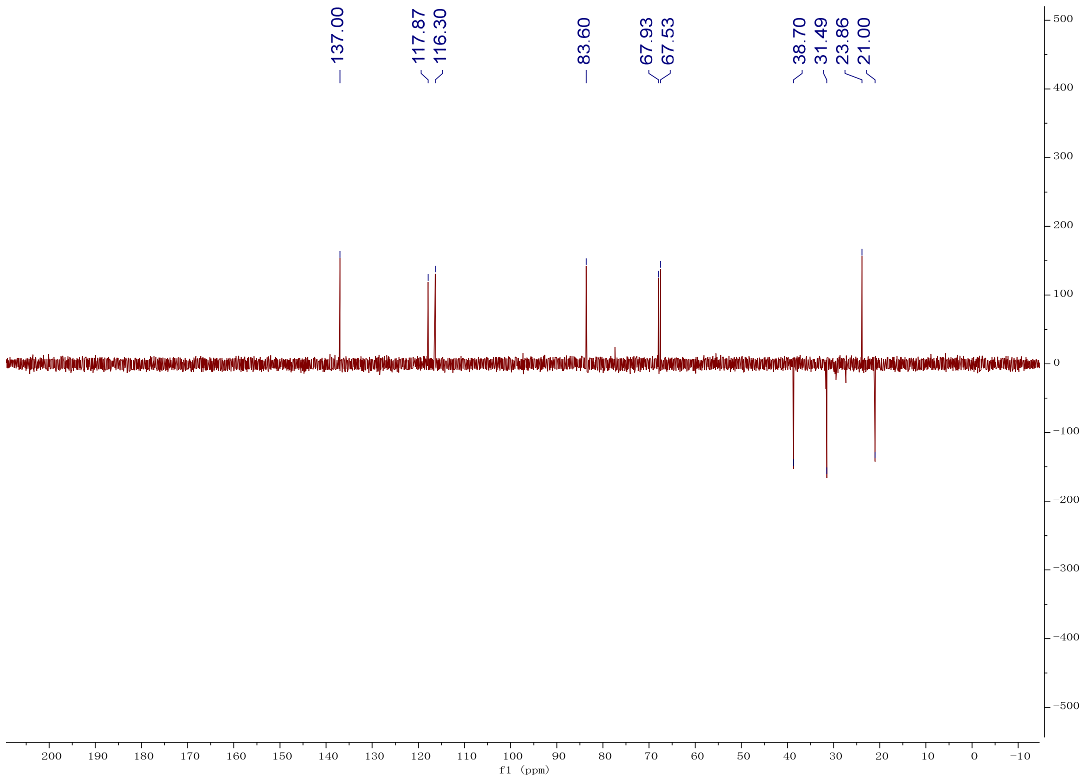


**Figure S43.** DEPT135 Spectrum of Aspergimarin E (**5**) in $CDCl_3$.

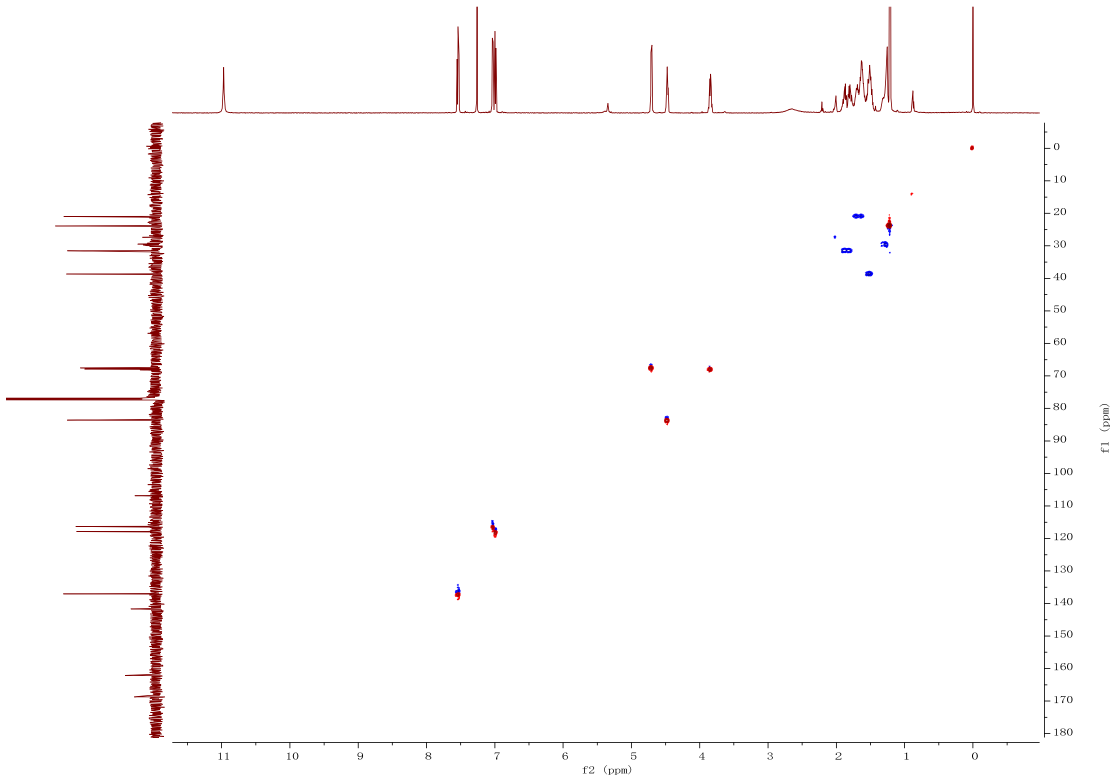


**Figure S44.** HSQC Spectrum of Aspergimarin E (**5**) in $CDCl_3$.

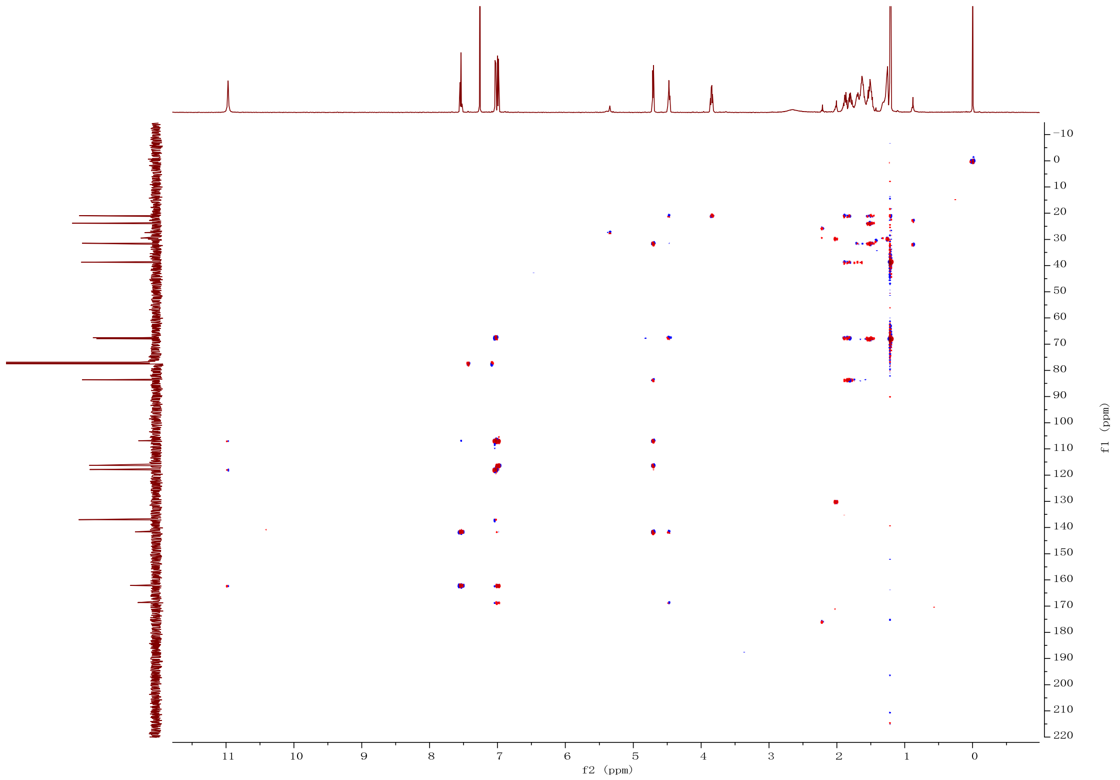


**Figure S45.** HMBC Spectrum of Aspergimarin E (**5**) in $CDCl_3$.

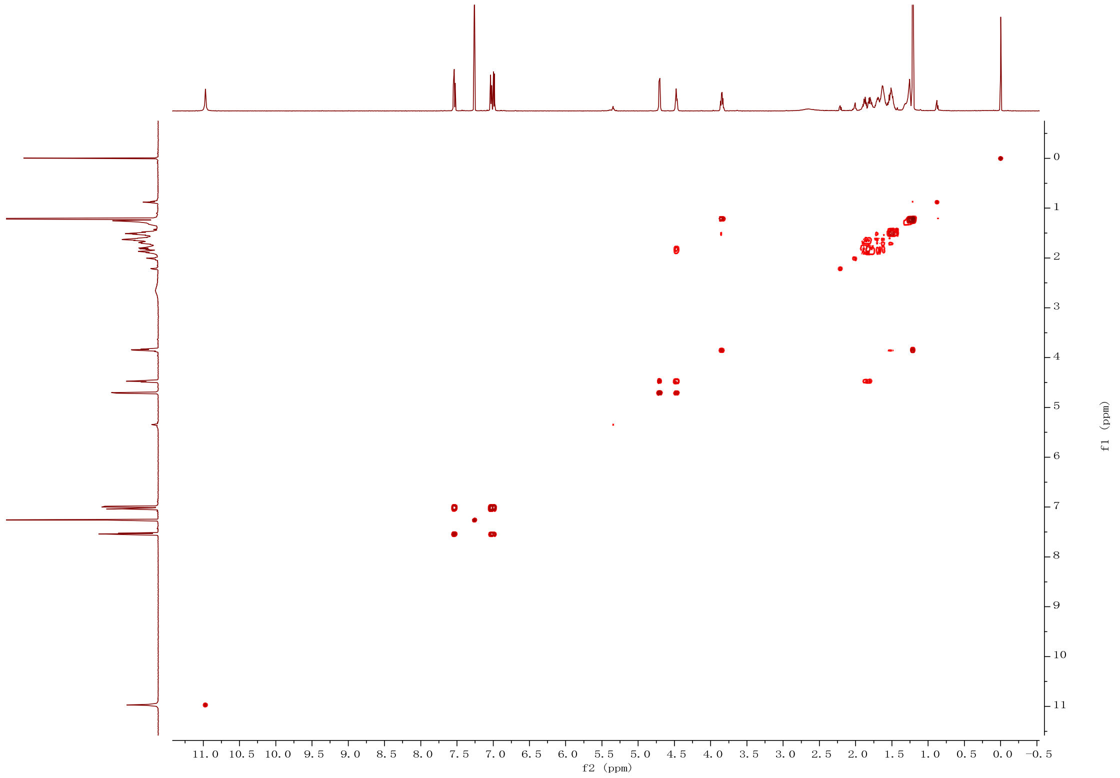


**Figure S46.** $^1H$-$^1H$ COSY Spectrum of Aspergimarin E (**5**) in $CDCl_3$.

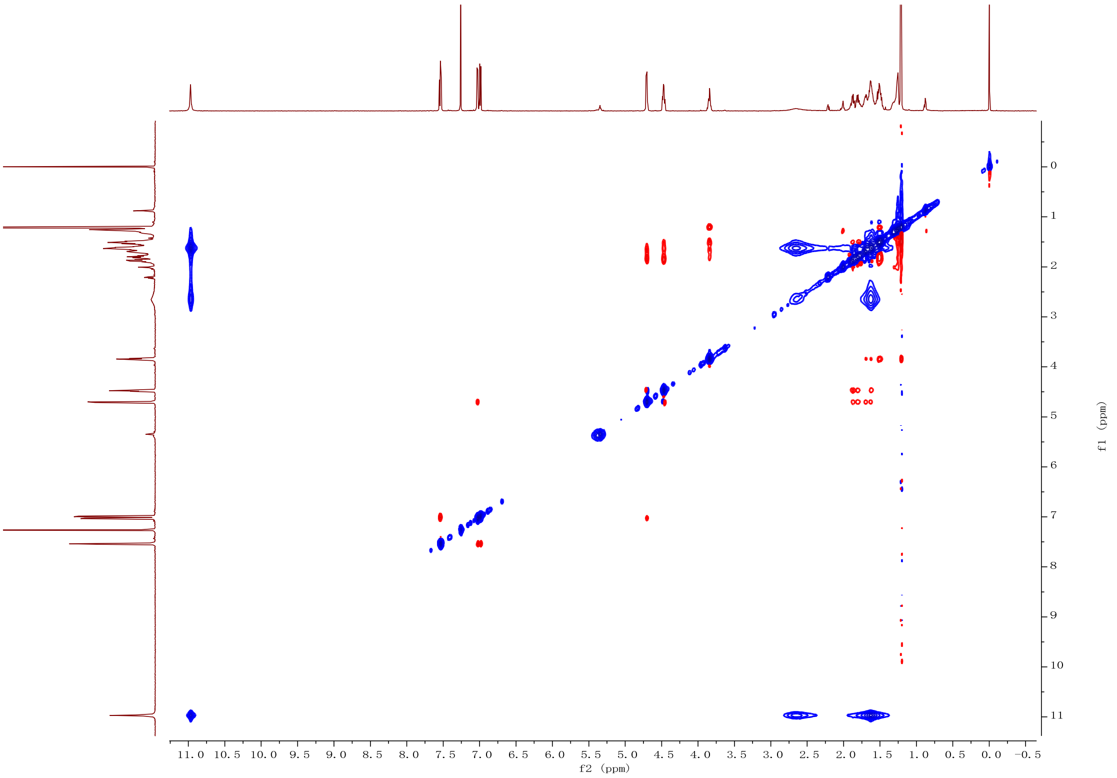


**Figure S47.** NOESY Spectrum of Aspergimarin E (**5**) in $CDCl_3$.

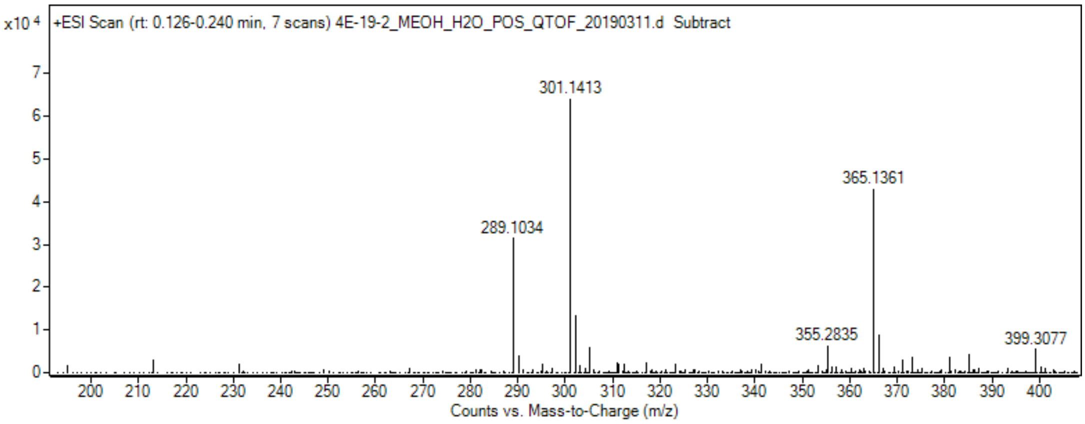


| Formula (M) | Species | m/z | Calc m/z | Diff (ppm) | Score (MFG) |
|---|---|---|---|---|---|
| C14 H18 O5 | (M+Na)+ | 289.1034 | 289.1046 | 4.56 | 93.13 |

**Figure S48.** HRESIMS of Aspergimarin E (**5**).

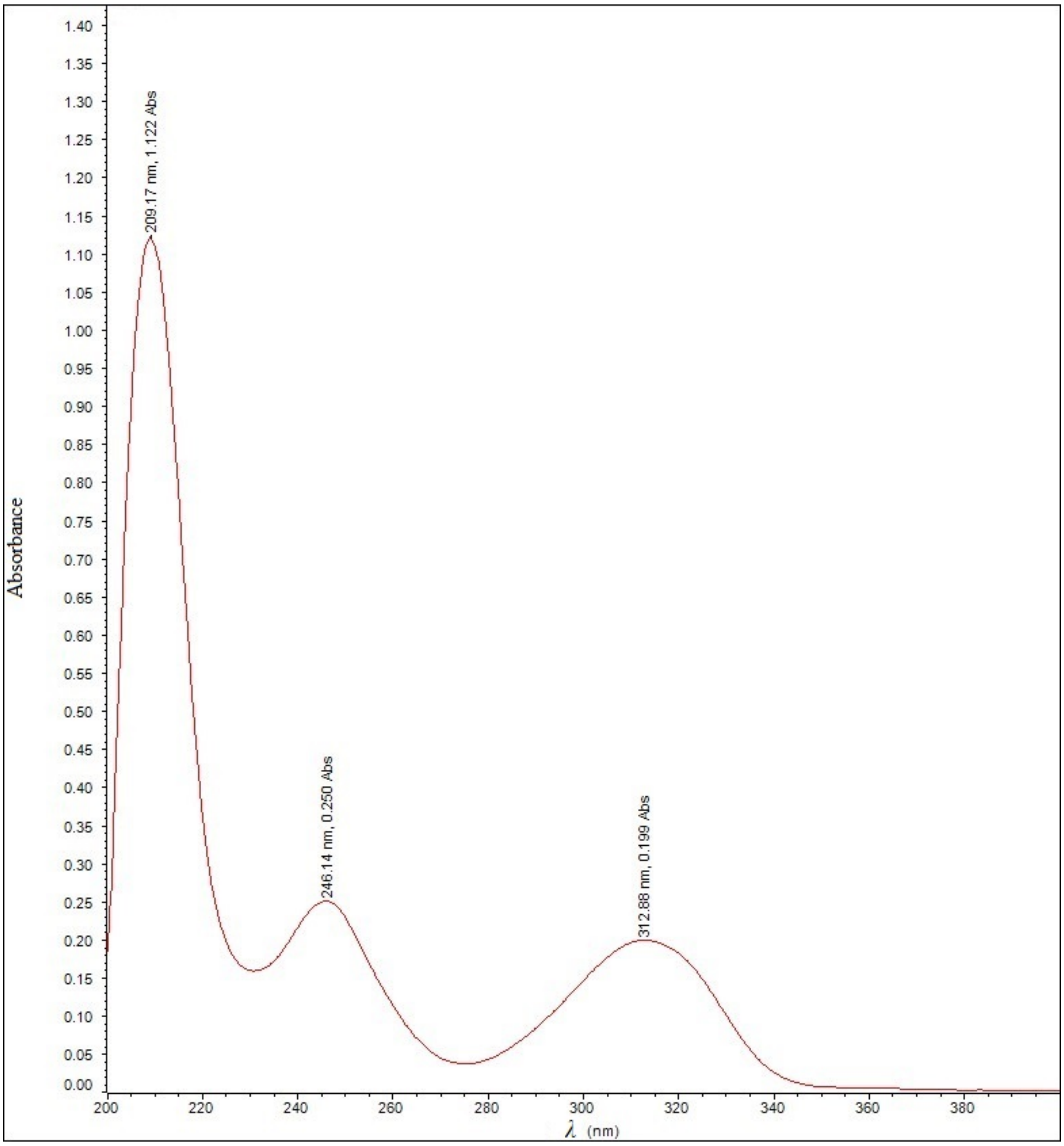


**Figure S49.** UV Spectrum of Aspergimarin E (**5**) in MeOH.

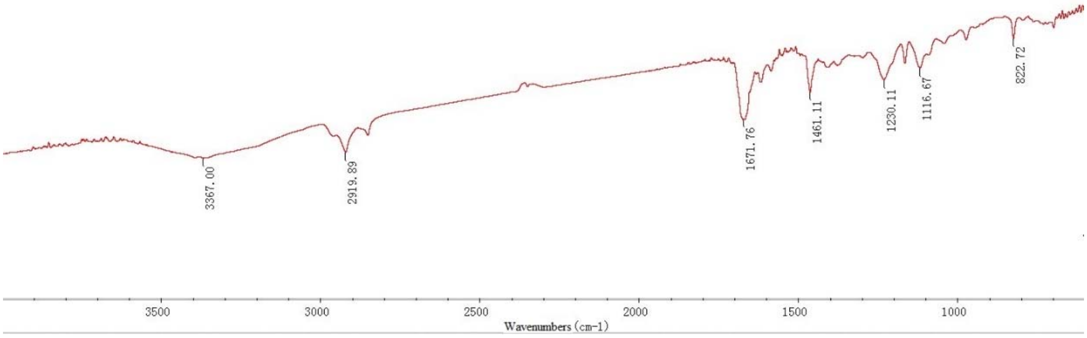


**Figure S50.** IR Spectrum of Aspergimarin E (**5**).

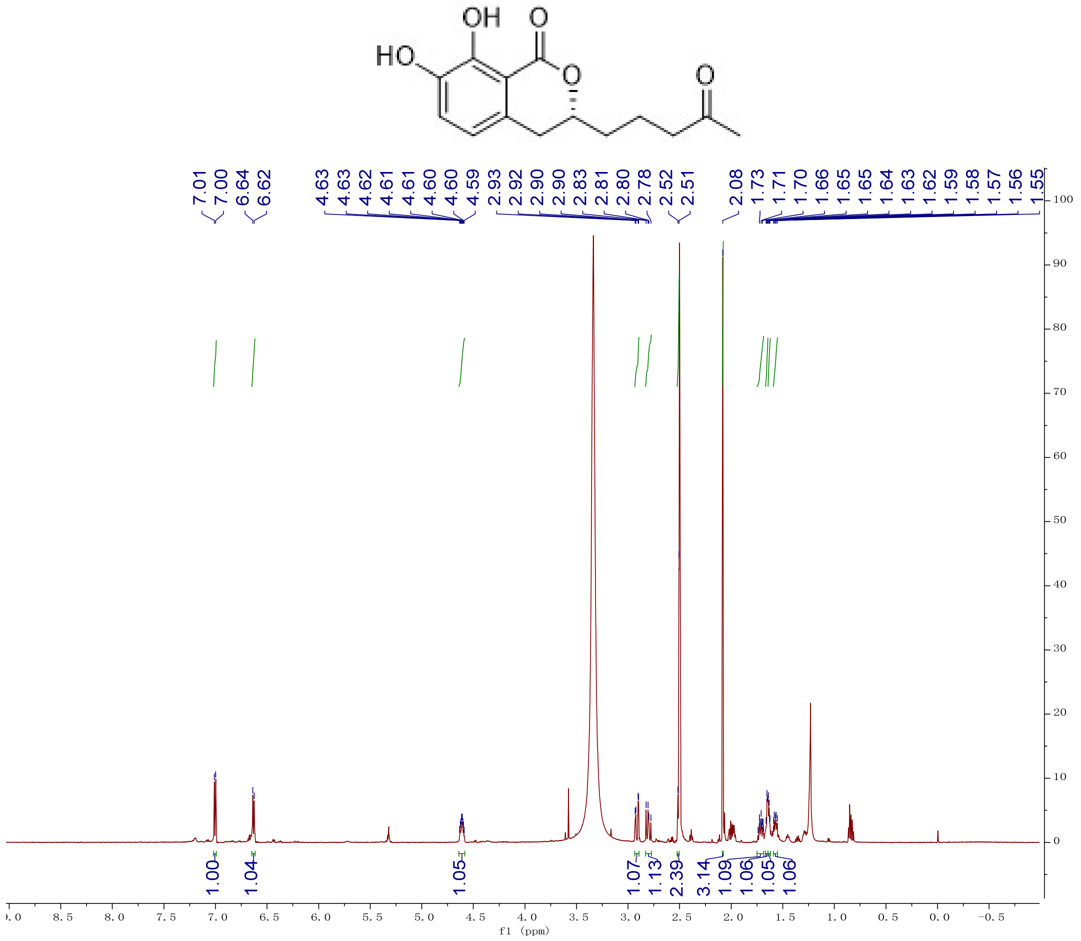


**Figure S51.** $^{1}$H NMR Spectrum of Aspergimarin F (**6**) in DMSO-$d_6$.

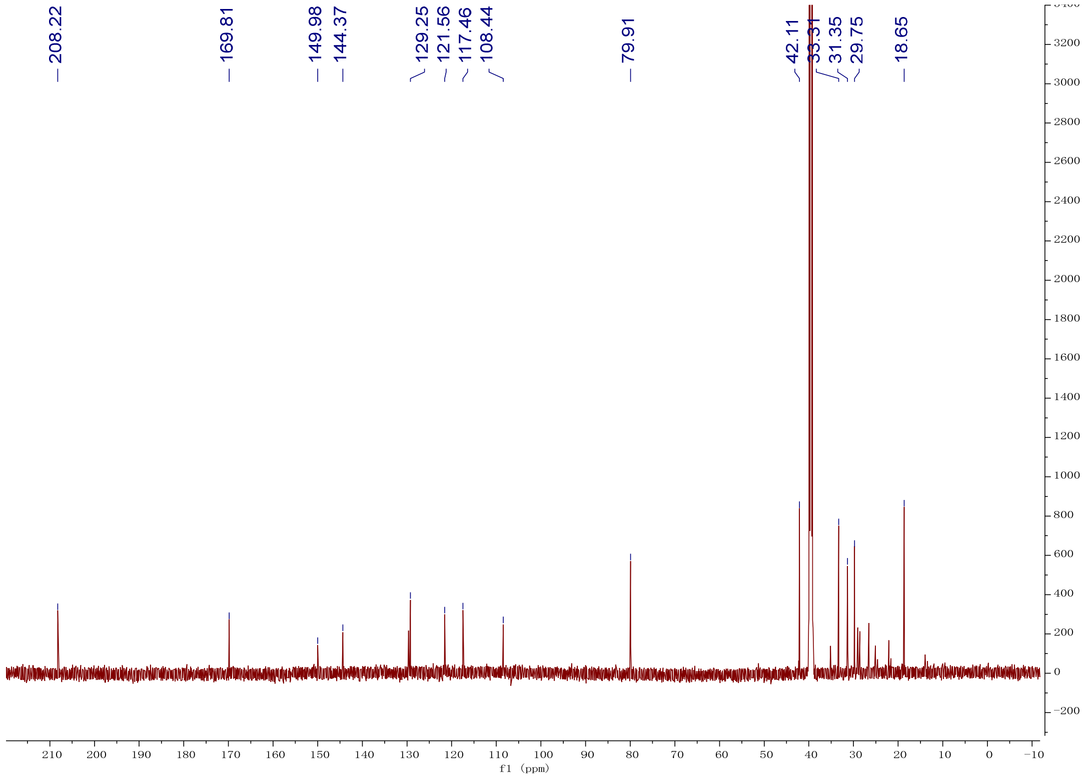


**Figure S52.** $^{13}$C NMR Spectrum of Aspergimarin F (**6**) in DMSO-$d_6$.

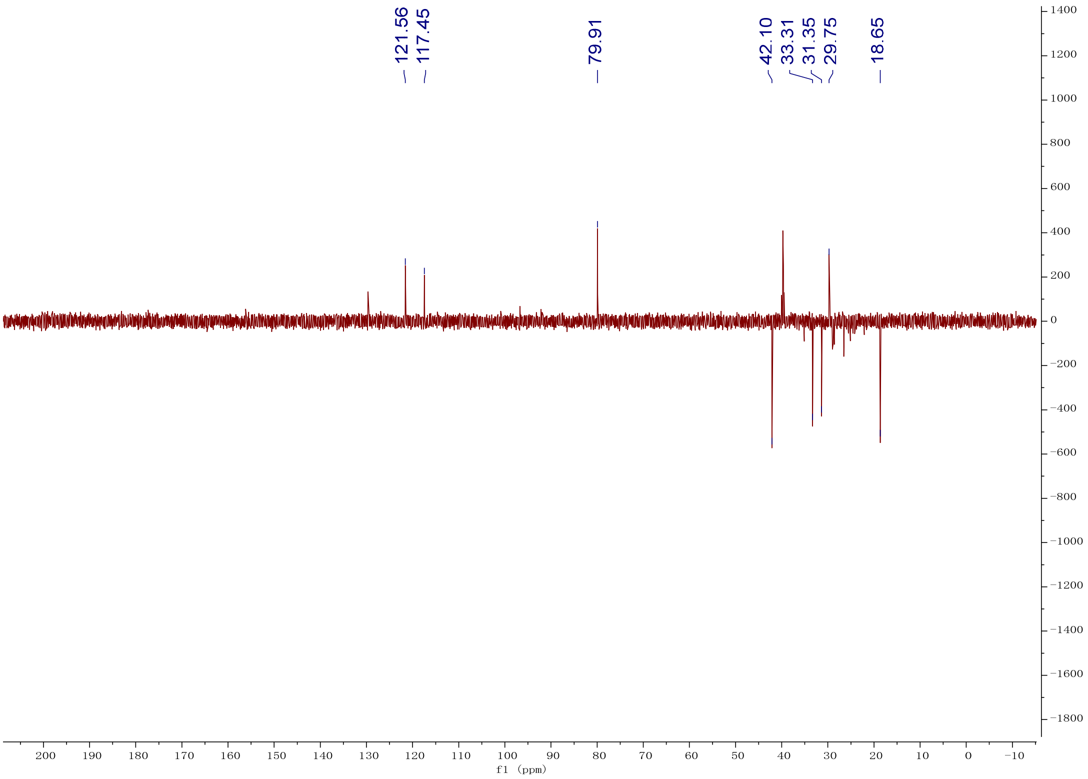


**Figure S53.** DEPT135 Spectrum of Aspergimarin F (**6**) in DMSO-$d_6$.

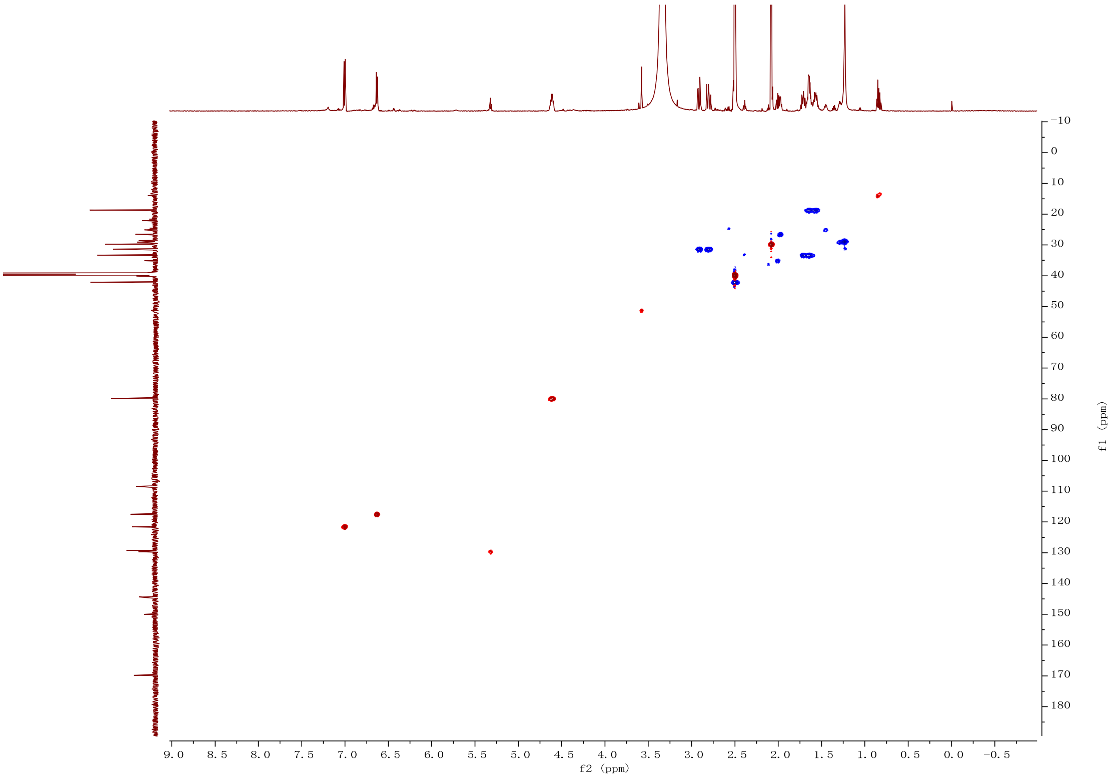


**Figure S54.** HSQC Spectrum of Aspergimarin F (**6**) in DMSO-$d_6$.

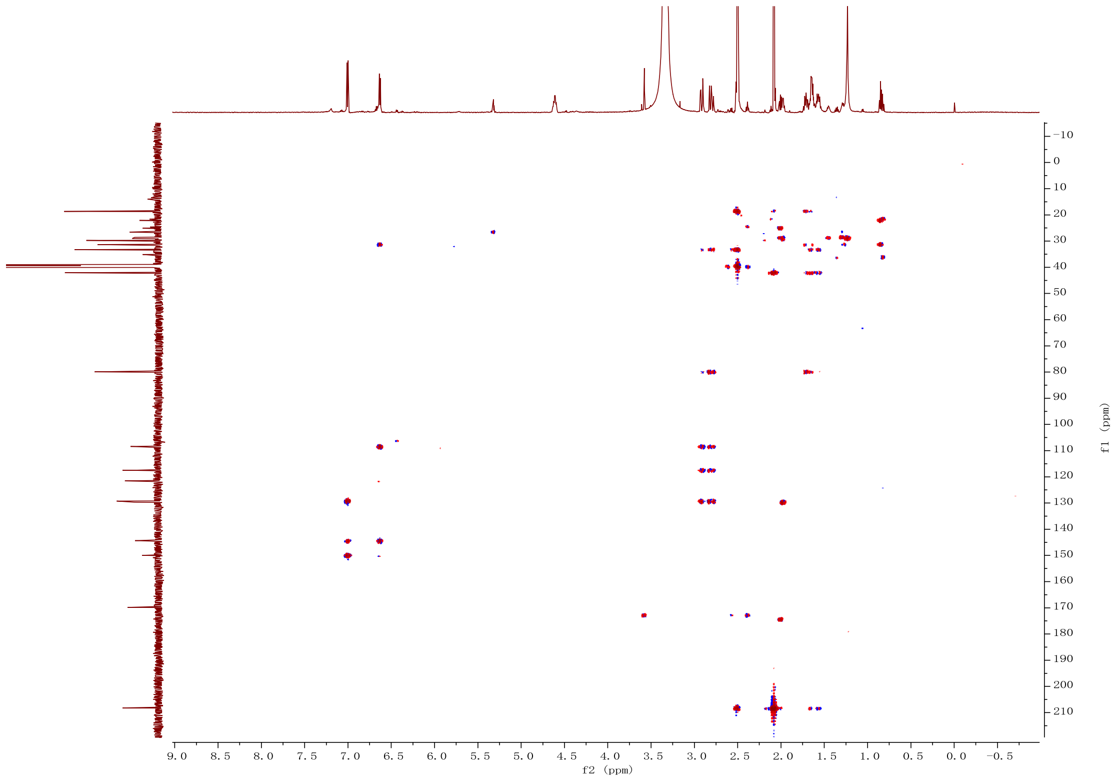

**Figure S55.** HMBC Spectrum of Aspergimarin F (**6**) in DMSO-$d_6$.

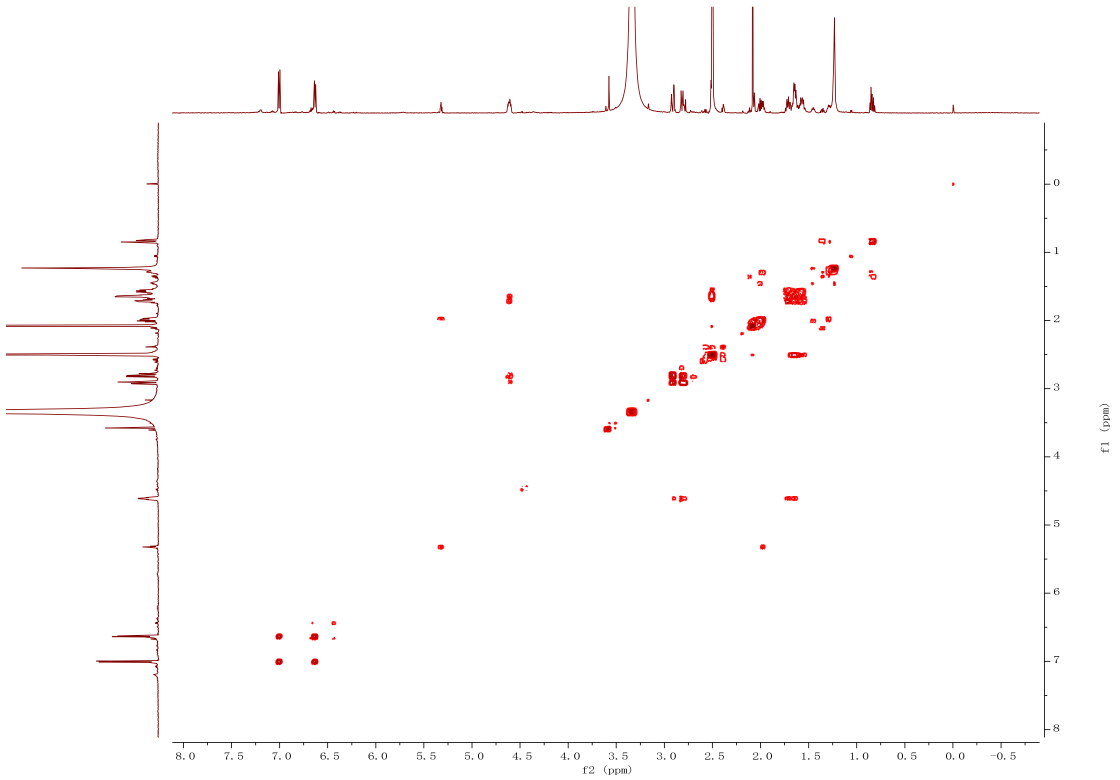

**Figure S56.** $^1$H-$^1$H COSY Spectrum of Aspergimarin F (**6**) in DMSO-$d_6$.

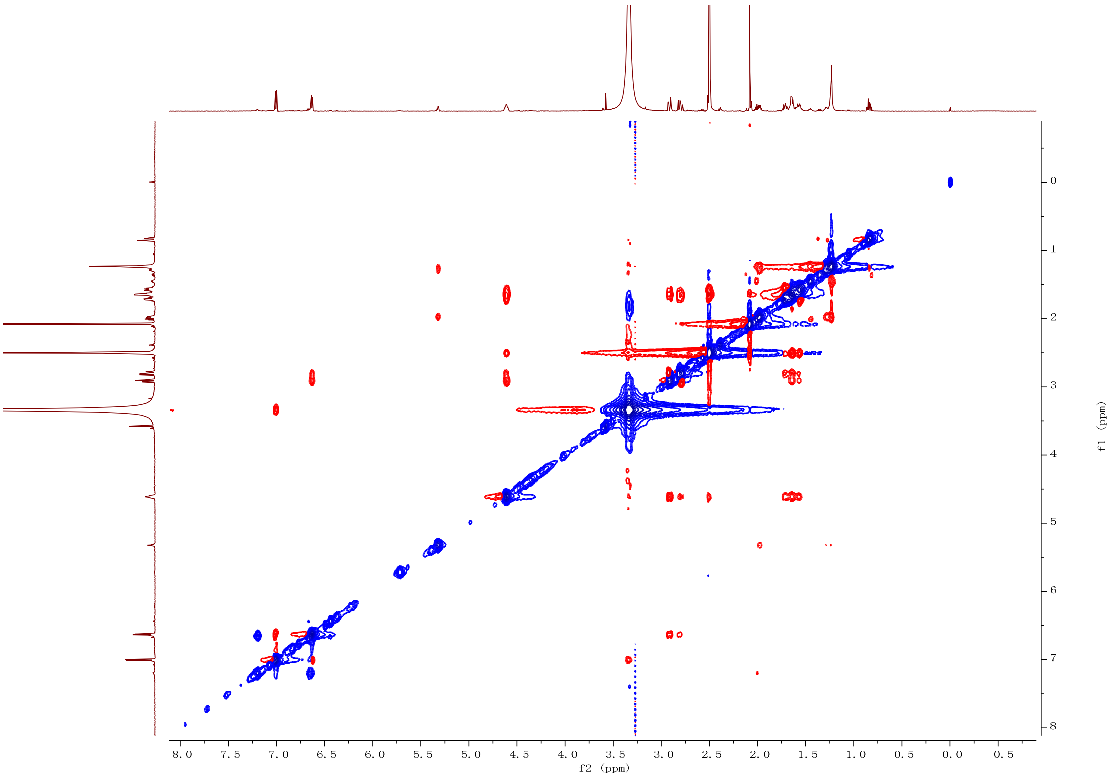


**Figure S57.** NOESY Spectrum of Aspergimarin F (**6**) in DMSO-$d_6$.

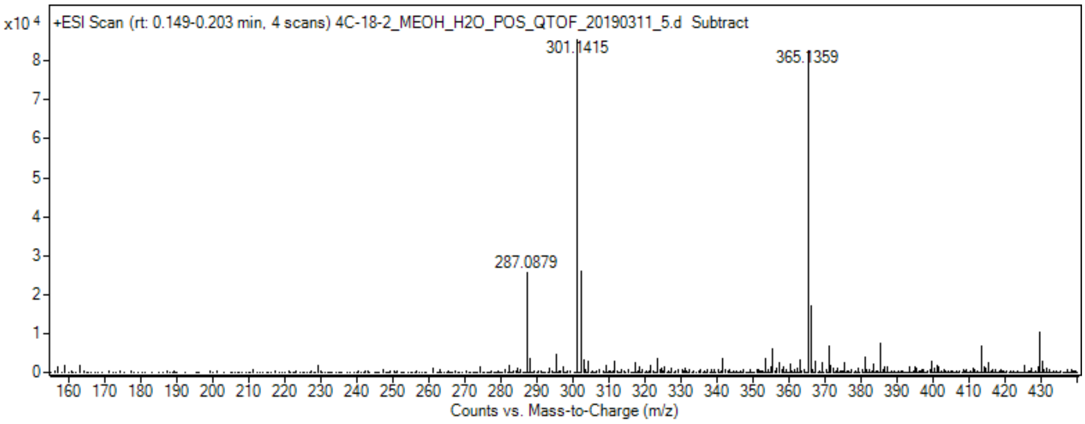


| Formula (M) | Species | m/z | Calc m/z | Diff (ppm) | Score (MFG) |
|---|---|---|---|---|---|
| C14 H16 O5 | (M+Na)+ | 287.0879 | 287.089 | 3.75 | 96.29 |

**Figure S58.** HRESIMS of Aspergimarin F (**6**).

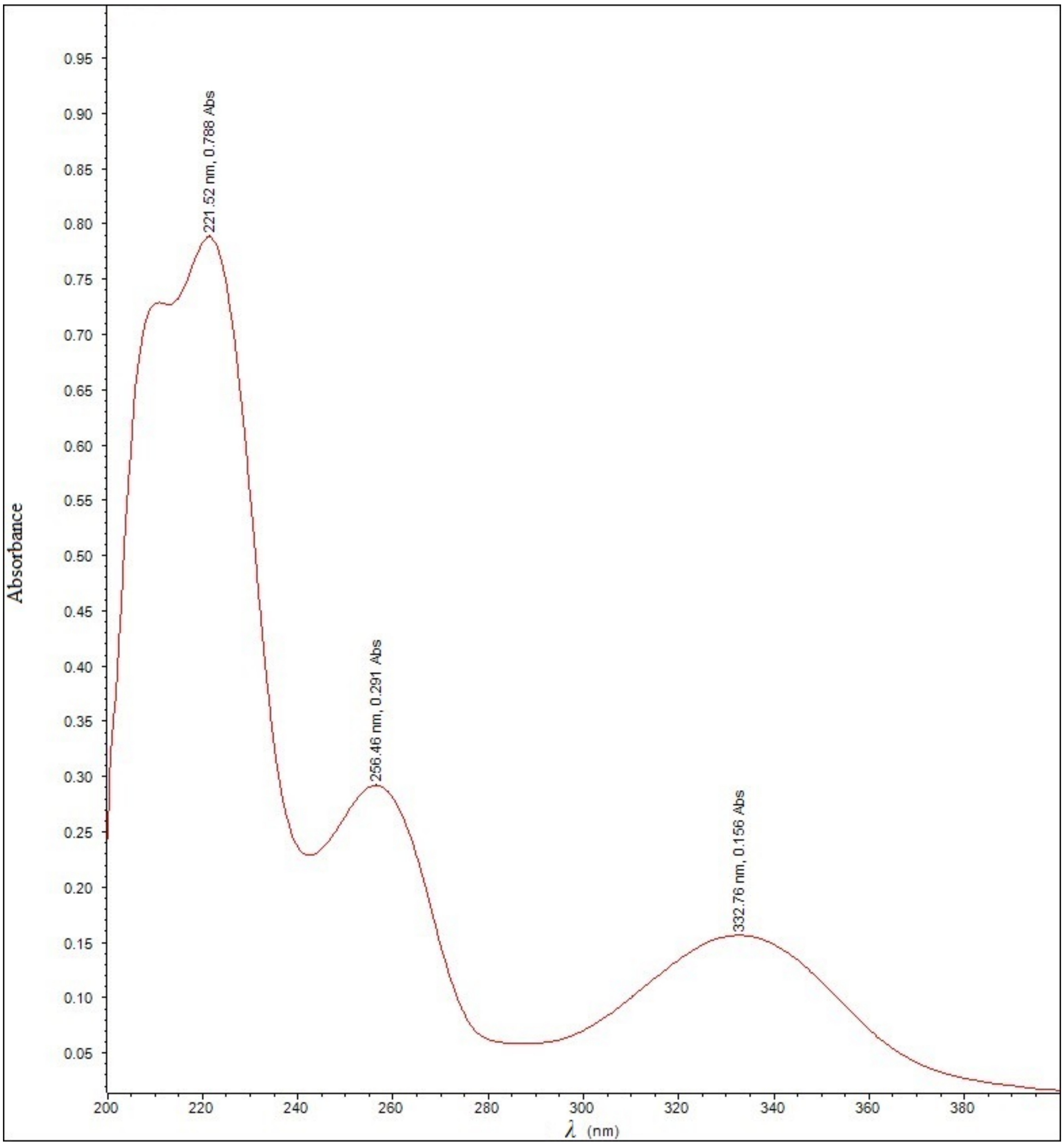


**Figure S59.** UV Spectrum of Aspergimarin F (**6**) in MeOH.

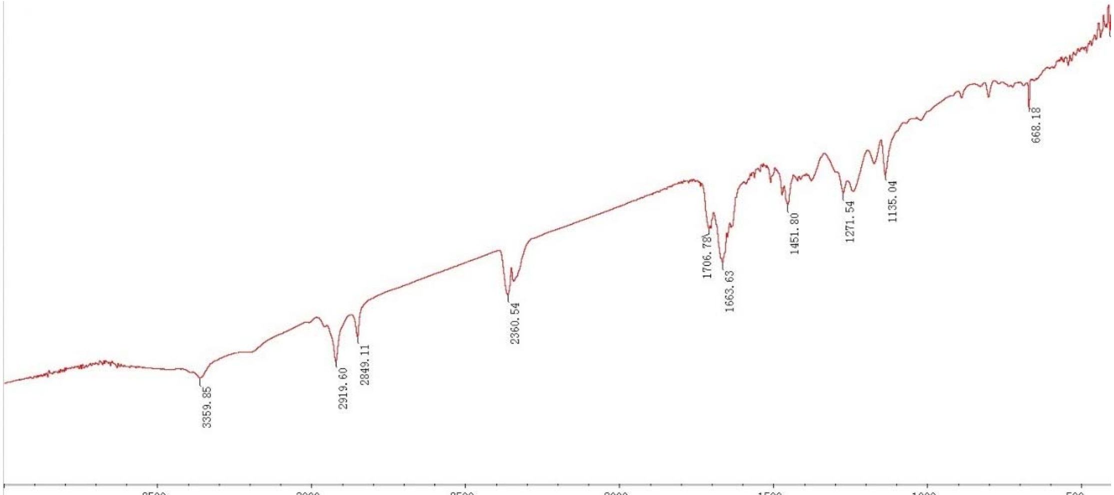


**Figure S60.** IR Spectrum of Aspergimarin F (**6**).

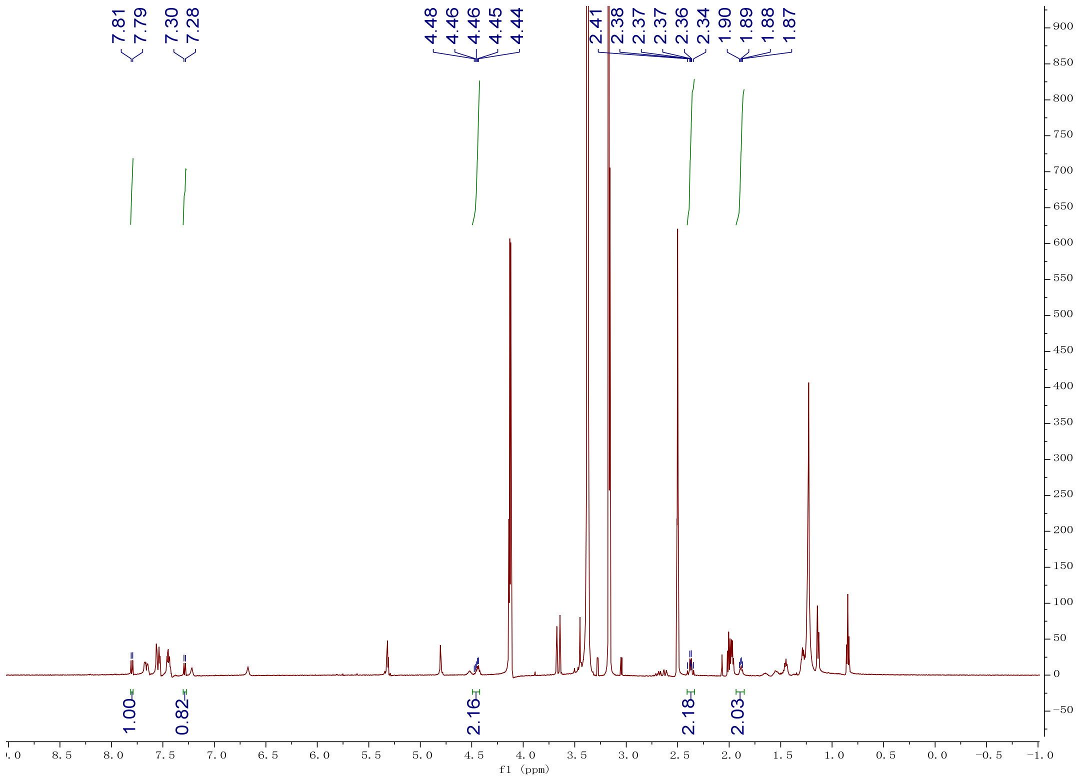


**Figure S61.** $^{1}$H NMR Spectrum of **3a** in DMSO-$d_6$ (600 MHz).

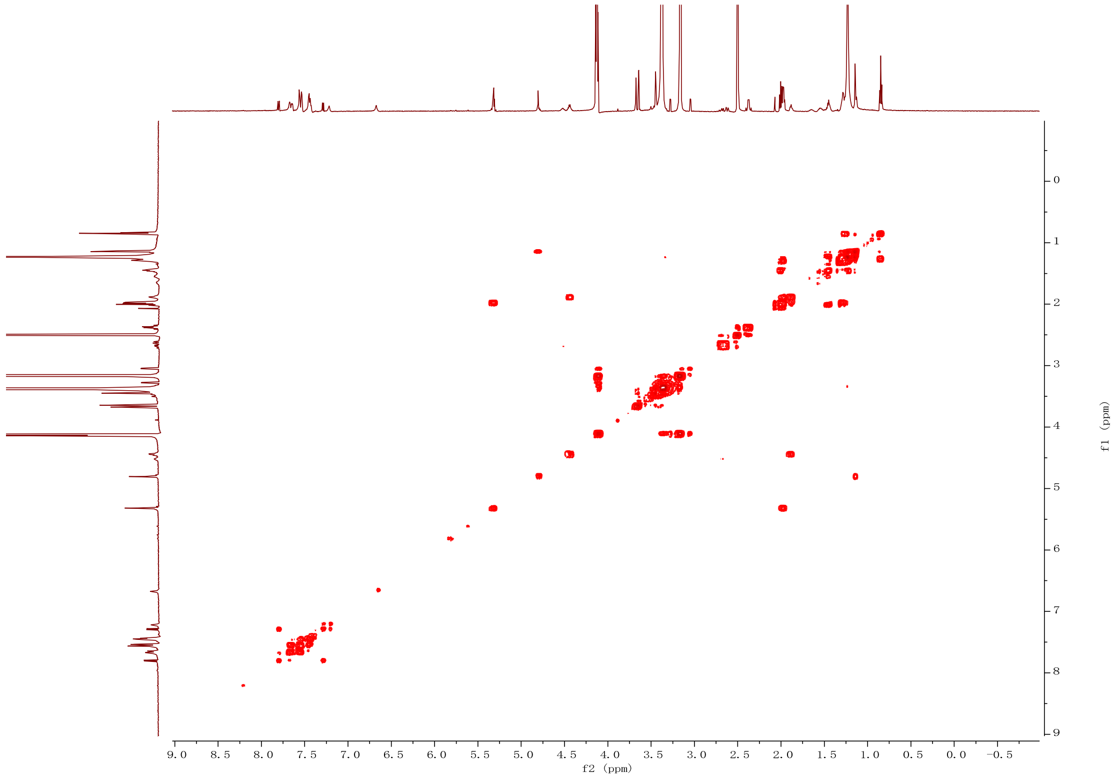


**Figure S62.** $^{1}$H-$^{1}$H COSY Spectrum of **3a** in DMSO-$d_6$ (600 MHz).

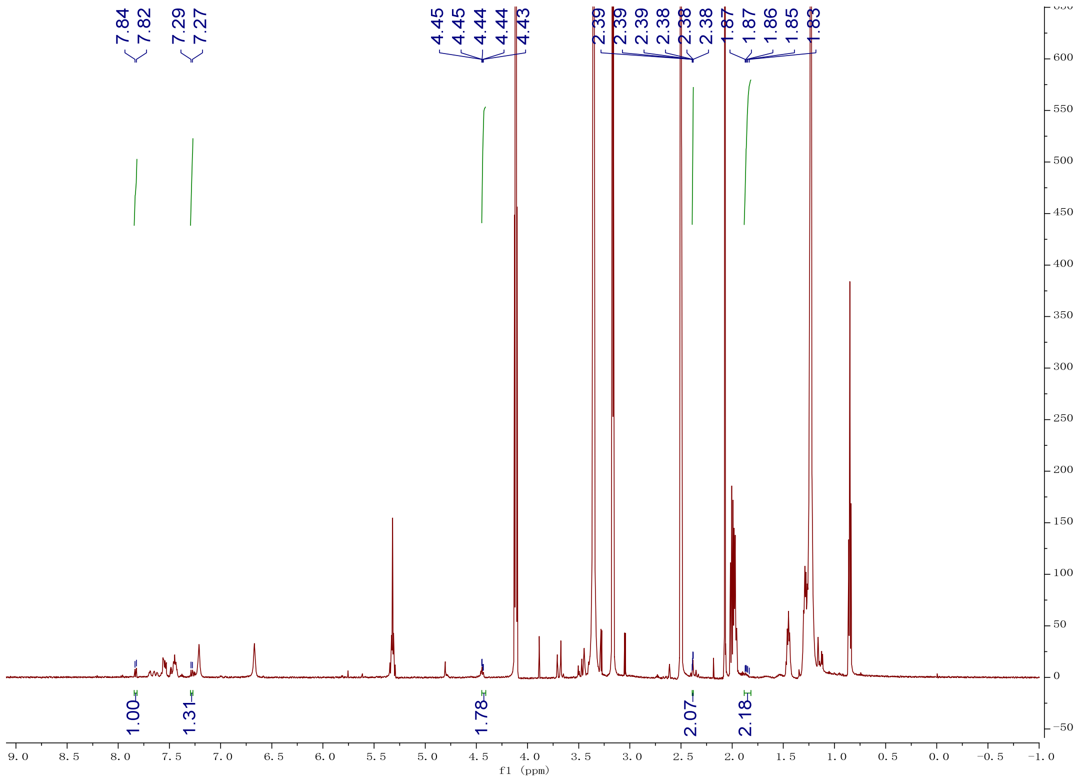


**Figure S63.** $^{1}H$ NMR Spectrum of **3b** in DMSO-$d_6$ (600 MHz).

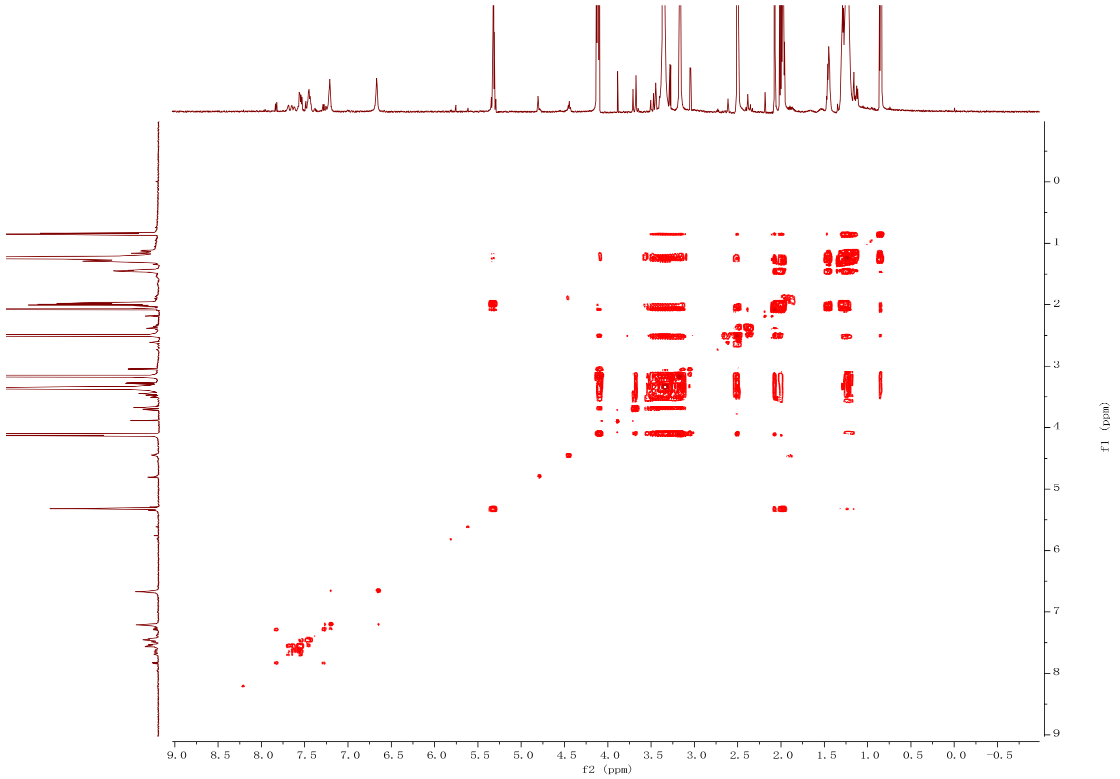


**Figure S64.** $^{1}H$-$^{1}H$ COSY Spectrum of **3b** in DMSO-$d_6$ (600 MHz).

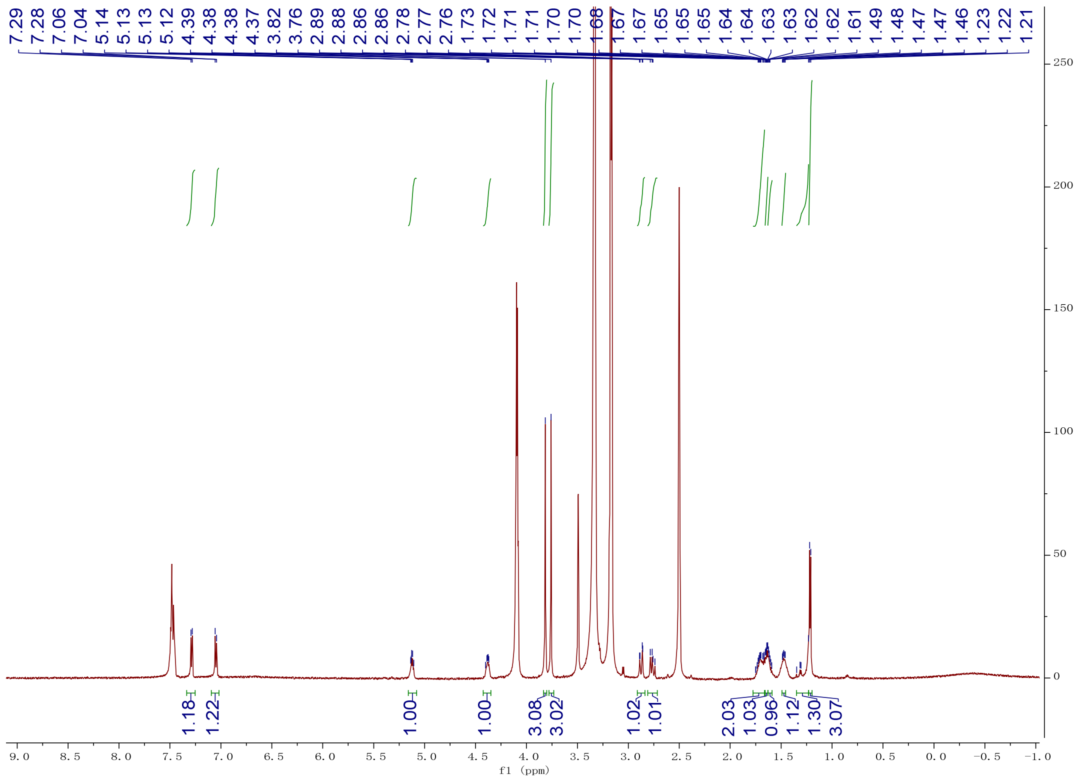

**Figure S65.** $^1$H NMR Spectrum of **4a** in DMSO-$d_6$ (600 MHz).

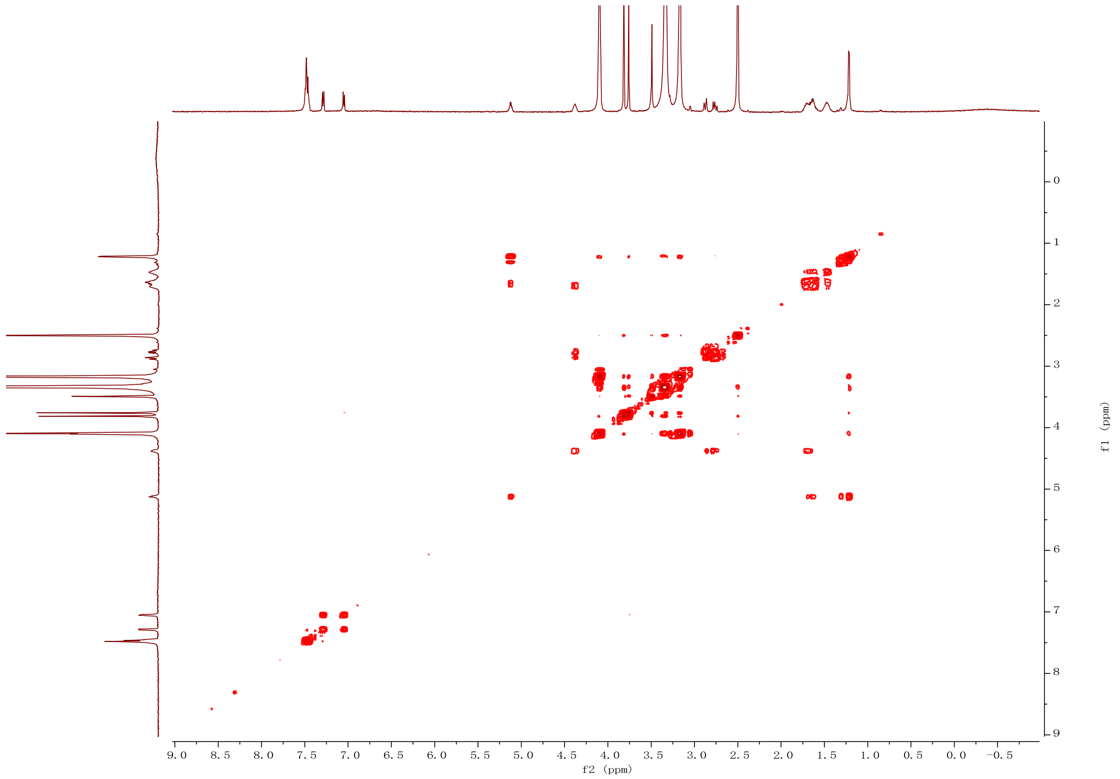

**Figure S66.** $^1$H-$^1$H COSY Spectrum of **4a** in DMSO-$d_6$ (600 MHz).

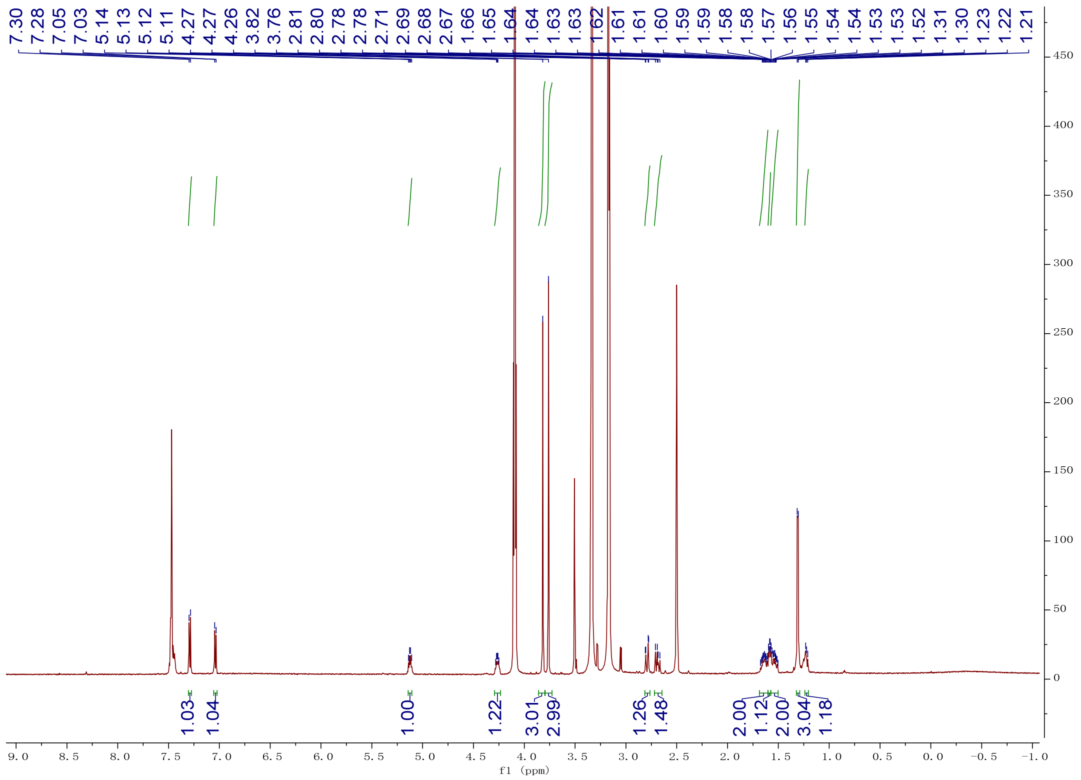

**Figure S67.** $^{1}$H NMR Spectrum of **4b** in DMSO-$d_6$ (600 MHz).

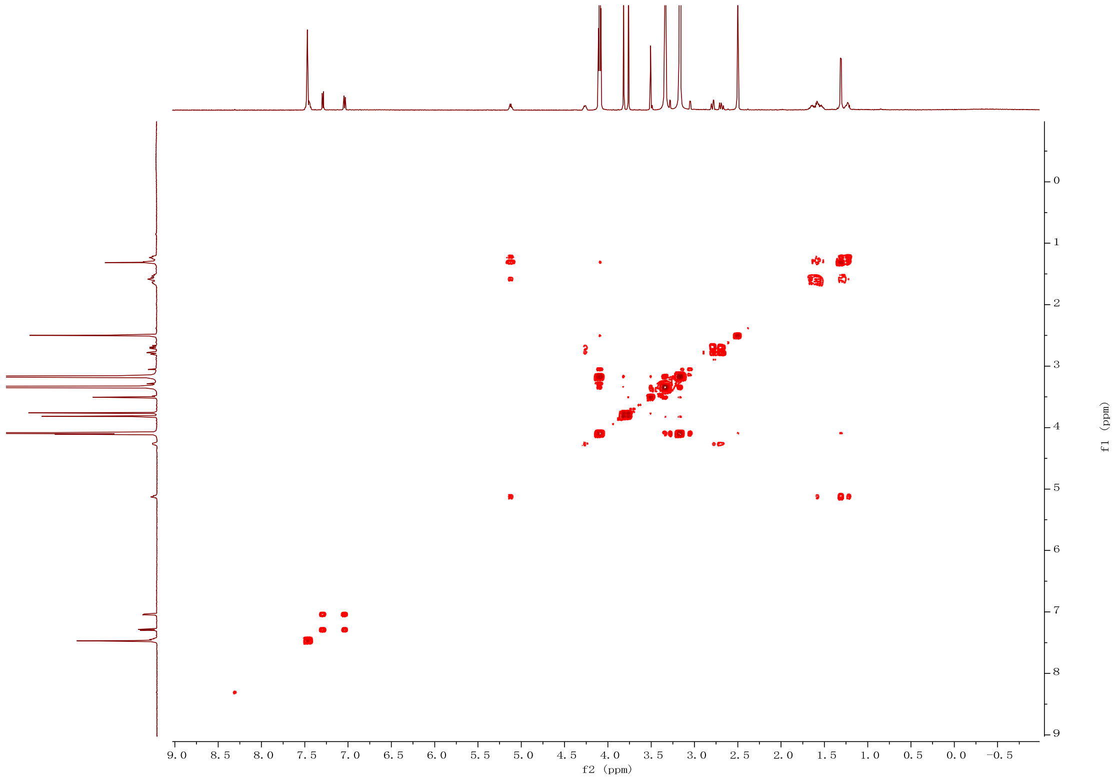

**Figure S68.** $^{1}$H-$^{1}$H COSY Spectrum of **4b** in DMSO-$d_6$ (600 MHz).

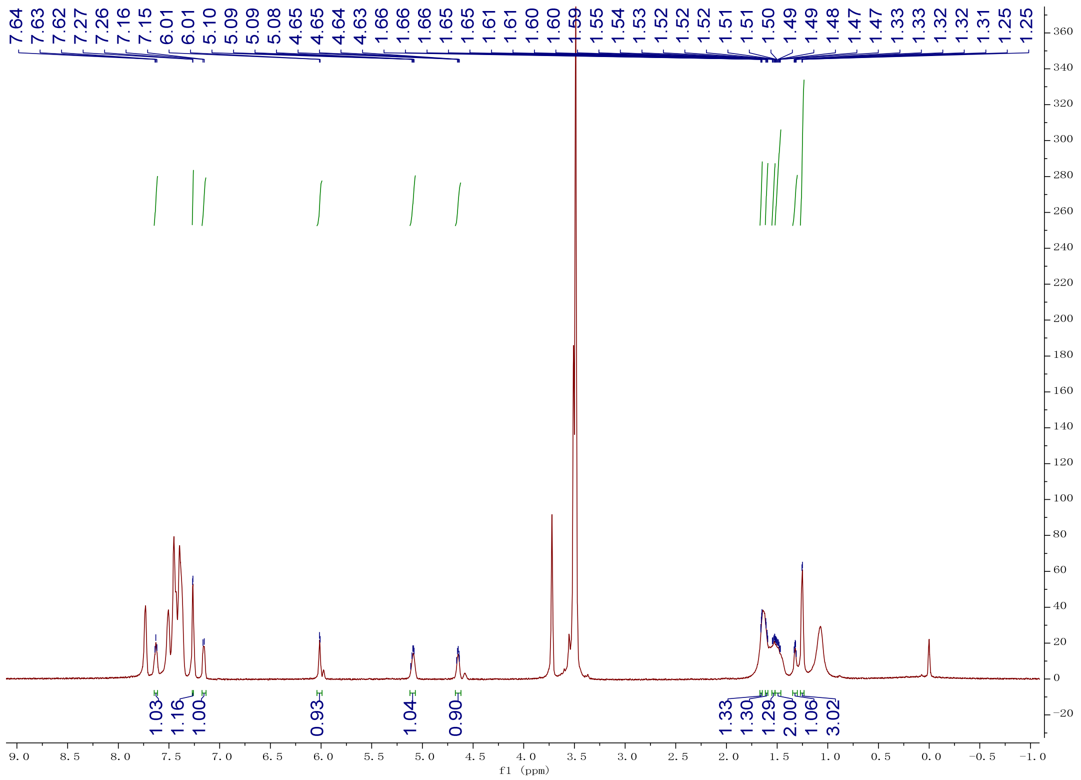

**Figure S69.** $^{1}$H NMR Spectrum of **5a** in $CDCl_3$ (600 MHz).

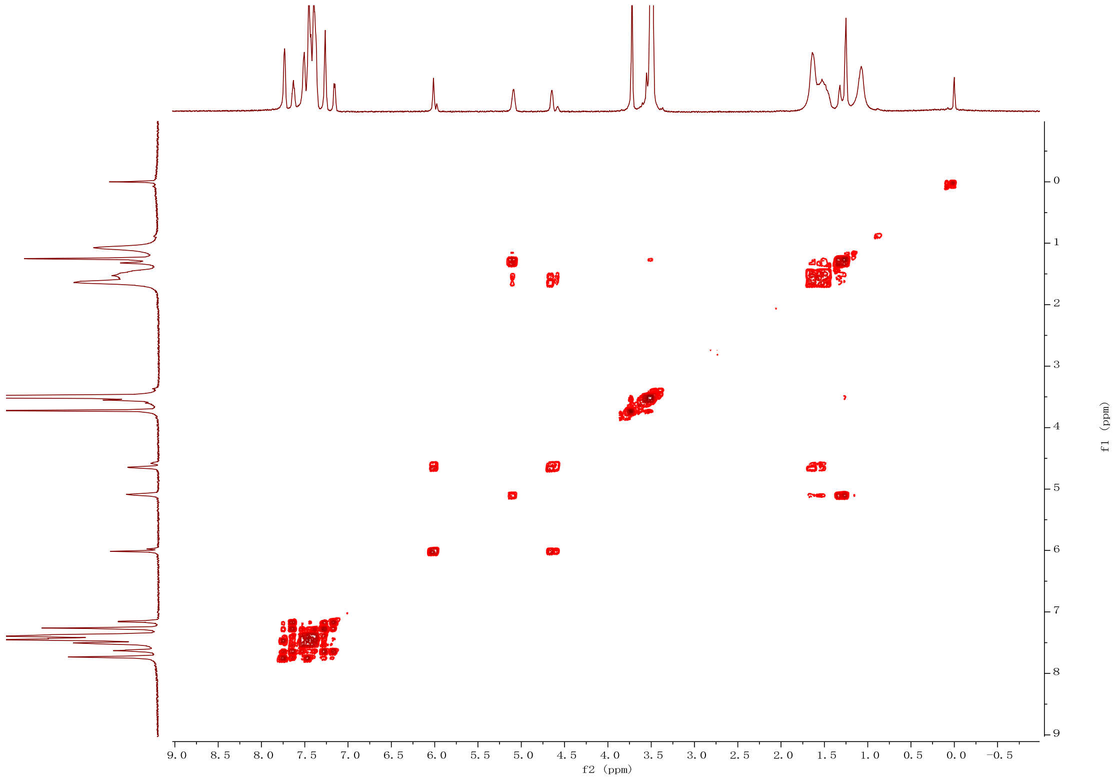

**Figure S70.** $^{1}$H-$^{1}$H COSY Spectrum of **5a** in $CDCl_3$ (600 MHz).

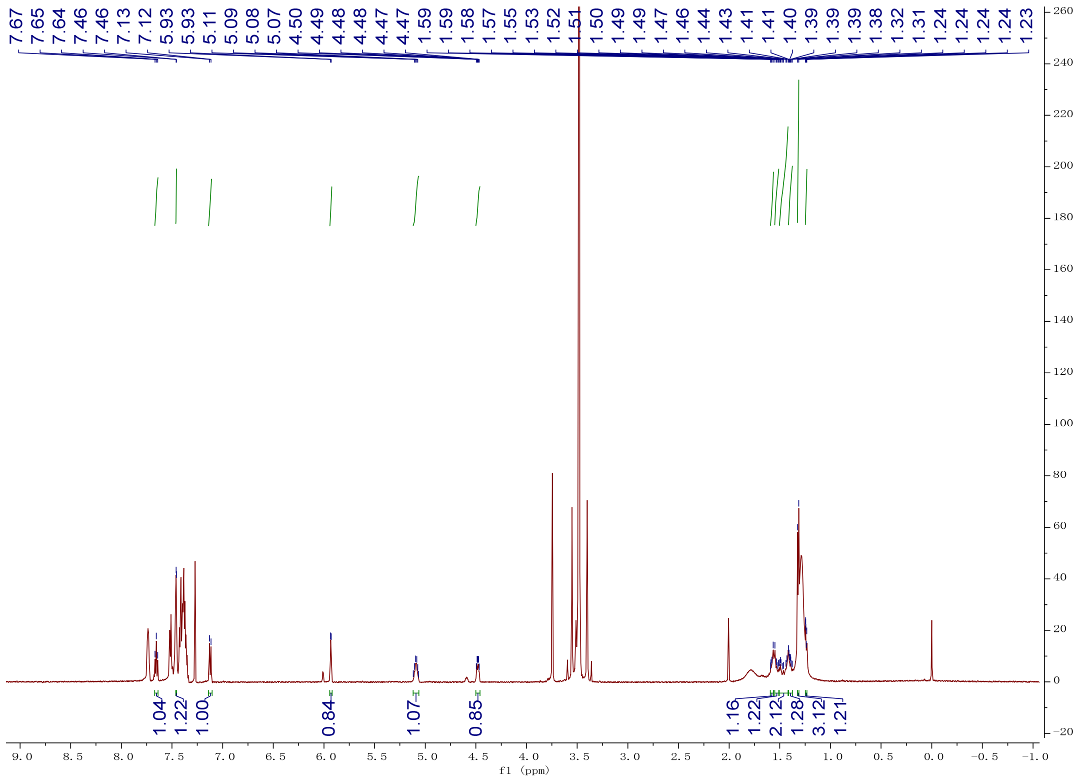

**Figure S71.** $^{1}$H NMR Spectrum of **5b** in $CDCl_3$ (600 MHz).

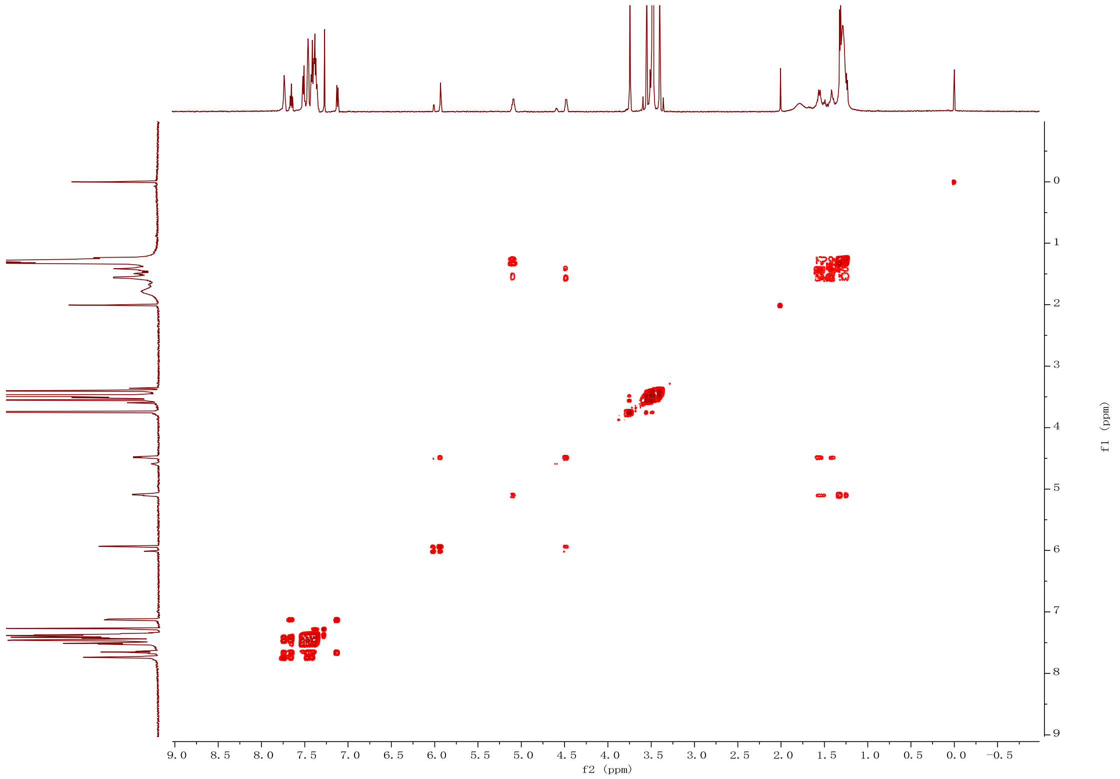

**Figure S72.** $^{1}$H-$^{1}$H COSY Spectrum of **5b** in $CDCl_3$ (600 MHz).

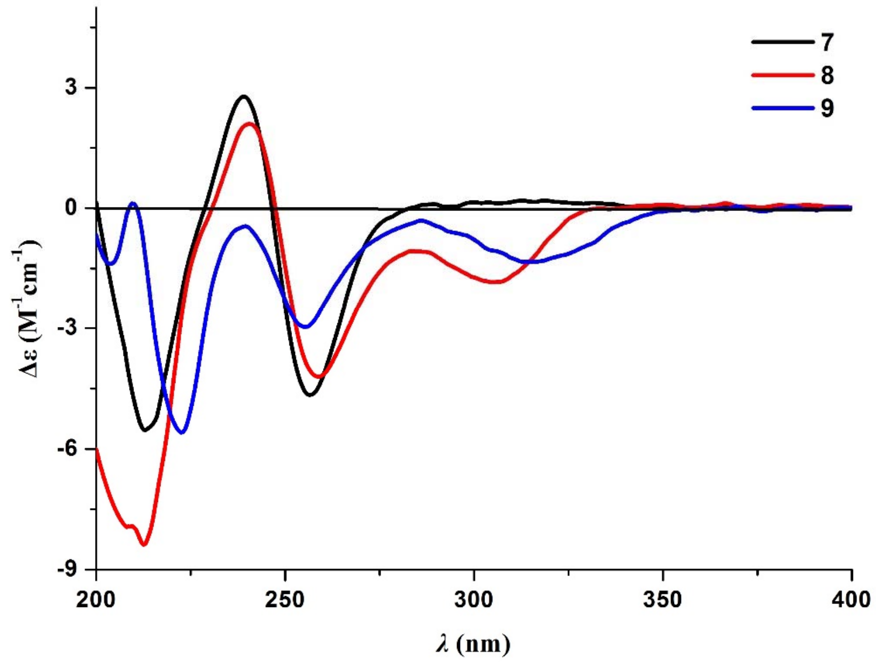


**Figure S73.** Experimental ECD spectra of **7−9**, collected in MeOH.
