# Supplementary material for: New Dihydroisocoumarin Root Growth Inhibitors From the Sponge-Derived Fungus Aspergillus sp. NBUF87
Source: Front Microbiol. 2019 Dec 10;10:2846. doi: 10.3389/fmicb.2019.02846 (PMC6914834; doi:10.3389/fmicb.2019.02846)
Supplement: Supplementary file 1 [file Data_Sheet_1.ZIP › Supplementary material/X-ray crystallography data of compound 1/checkCIF-PLATON page 2.htm]

checkCIF/PLATON page 2


# checkCIF (basic structural check) running

---

  
*Checking for embedded fcf data in CIF ...*
  
*Found embedded fcf data in CIF. Extracting fcf data from uploaded CIF, please wait*
**.**
**.**

# checkCIF/PLATON (basic structural check)

---

Structure factors have been supplied for datablock(s) cu\_dd18259\_0m

THIS REPORT IS FOR GUIDANCE ONLY. IF USED AS PART OF A REVIEW PROCEDURE FOR PUBLICATION, IT SHOULD NOT REPLACE THE EXPERTISE OF AN EXPERIENCED CRYSTALLOGRAPHIC REFEREE.

```
No syntax errors found.                               CIF dictionary  
Please wait while processing ....                     Interpreting this report
```

Structure factor report  
