# Supplementary material for: New Dihydroisocoumarin Root Growth Inhibitors From the Sponge-Derived Fungus Aspergillus sp. NBUF87
Source: Front Microbiol. 2019 Dec 10;10:2846. doi: 10.3389/fmicb.2019.02846 (PMC6914834; doi:10.3389/fmicb.2019.02846)
Supplement: Supplementary file 1 [file Data_Sheet_1.ZIP › Supplementary material/X-ray crystallography data of compound 1/cu_dd18259_0m.rtf]

  Table 1.  Crystal data and structure refinement for cu_dd18259_0m.
Identification code 	cu_dd18259_0m
Empirical formula 	C14 H18 O5
Formula weight 	266.28
Temperature 	293(2) K
Wavelength 	1.54178 Å
Crystal system 	Monoclinic
Space group 	P 21
Unit cell dimensions	a = 4.9195(3) Å	a= 90°.
	b = 24.7434(17) Å	b= 101.812(2)°.
	c = 5.6478(4) Å	g = 90°.
Volume	672.92(8) Å3
Z	2
Density (calculated)	1.314 Mg/m3
Absorption coefficient	0.829 mm-1
F(000)	284
Crystal size	0.200 x 0.170 x 0.130 mm3
Theta range for data collection	10.927 to 67.470°.
Index ranges	-5<=h<=5, -29<=k<=29, -6<=l<=6
Reflections collected	8611
Independent reflections	2294 [R(int) = 0.0377]
Completeness to theta = 67.679°	95.4 % 
Absorption correction	Semi-empirical from equivalents
Max. and min. transmission	0.7533 and 0.5150
Refinement method	Full-matrix least-squares on F2
Data / restraints / parameters	2294 / 67 / 205
Goodness-of-fit on F2	1.109
Final R indices [I>2sigma(I)]	R1 = 0.0717, wR2 = 0.2076
R indices (all data)	R1 = 0.0719, wR2 = 0.2079
Absolute structure parameter	0.02(5)
Extinction coefficient	n/a
Largest diff. peak and hole	1.035 and -0.219 e.Å-3

 Table 2.  Atomic coordinates  ( x 104) and equivalent  isotropic displacement parameters (Å2x 103)
for cu_dd18259_0m.  U(eq) is defined as one third of  the trace of the orthogonalized Uij tensor.
________________________________________________________________________________ 
	x	y	z	U(eq)
________________________________________________________________________________  
O(1)	4519(9)	3258(2)	7227(8)	57(1)
O(2)	8558(8)	4627(2)	1067(6)	47(1)
O(3)	1931(7)	4102(1)	8455(6)	45(1)
O(4)	2611(7)	4911(1)	7179(6)	45(1)
C(1)	5525(10)	3603(2)	5769(8)	41(1)
C(2)	7208(12)	3399(2)	4270(10)	47(1)
C(3)	8239(11)	3743(2)	2747(9)	45(1)
C(4)	7630(9)	4285(2)	2615(8)	38(1)
C(5)	5964(9)	4504(2)	4080(8)	36(1)
C(6)	4902(10)	4166(2)	5689(8)	37(1)
C(7)	3110(9)	4384(2)	7190(8)	38(1)
C(8)	4521(10)	5282(2)	6323(9)	42(1)
C(9)	5185(10)	5089(2)	3961(8)	41(1)
C(10)	3155(14)	5835(2)	6079(11)	55(1)
C(11)	2440(20)	6058(3)	8405(16)	83(2)
C(12)	1230(20)	6629(3)	8023(18)	84(2)
O(5)	370(30)	6771(5)	11620(20)	91(3)
C(13)	-890(30)	6846(6)	9230(30)	75(3)
C(14)	-1490(50)	7439(6)	8690(40)	85(3)
O(5')	3580(30)	6966(5)	12370(20)	101(3)
C(13')	1190(40)	6893(6)	10520(30)	77(3)
C(14')	-350(40)	7420(6)	10270(40)	78(3)
________________________________________________________________________________ 
 Table 3.   Bond lengths [Å] and angles [°] for  cu_dd18259_0m.
_____________________________________________________ 
O(1)-C(1) 	1.348(6)
O(1)-H(1) 	0.8200
O(2)-C(4) 	1.361(6)
O(2)-H(2) 	0.8200
O(3)-C(7) 	1.226(6)
O(4)-C(7) 	1.328(6)
O(4)-C(8) 	1.464(6)
C(1)-C(2) 	1.393(7)
C(1)-C(6) 	1.425(7)
C(2)-C(3) 	1.378(8)
C(2)-H(2A) 	0.9300
C(3)-C(4) 	1.374(7)
C(3)-H(3) 	0.9300
C(4)-C(5) 	1.388(6)
C(5)-C(6) 	1.412(6)
C(5)-C(9) 	1.497(7)
C(6)-C(7) 	1.447(6)
C(8)-C(9) 	1.514(6)
C(8)-C(10) 	1.518(7)
C(8)-H(8) 	0.9800
C(9)-H(9A) 	0.9700
C(9)-H(9B) 	0.9700
C(10)-C(11) 	1.531(9)
C(10)-H(10A) 	0.9700
C(10)-H(10B) 	0.9700
C(11)-C(12) 	1.531(10)
C(11)-H(11A) 	0.9700
C(11)-H(11B) 	0.9700
C(12)-C(13) 	1.458(14)
C(12)-C(13') 	1.558(16)
C(12)-H(12A) 	0.9604
C(12)-H(12B) 	0.9590
C(12)-H(12C) 	0.9598
C(12)-H(12D) 	0.9600
O(5)-C(13) 	1.381(17)
O(5)-H(5) 	0.8258
C(13)-C(14) 	1.515(17)
C(13)-H(13) 	0.9800
C(14)-H(14A) 	0.9600
C(14)-H(14B) 	0.9600
C(14)-H(14C) 	0.9600
O(5')-C(13') 	1.418(19)
O(5')-H(5') 	0.8201
C(13')-C(14') 	1.498(17)
C(13')-H(13') 	0.9800
C(14')-H(14D) 	0.9600
C(14')-H(14E) 	0.9600
C(14')-H(14F) 	0.9600

C(1)-O(1)-H(1)	109.5
C(4)-O(2)-H(2)	109.5
C(7)-O(4)-C(8)	119.1(4)
O(1)-C(1)-C(2)	118.8(4)
O(1)-C(1)-C(6)	122.3(4)
C(2)-C(1)-C(6)	119.0(4)
C(3)-C(2)-C(1)	119.8(4)
C(3)-C(2)-H(2A)	120.1
C(1)-C(2)-H(2A)	120.1
C(4)-C(3)-C(2)	122.2(4)
C(4)-C(3)-H(3)	118.9
C(2)-C(3)-H(3)	118.9
O(2)-C(4)-C(3)	123.0(4)
O(2)-C(4)-C(5)	117.2(4)
C(3)-C(4)-C(5)	119.8(4)
C(4)-C(5)-C(6)	119.6(4)
C(4)-C(5)-C(9)	121.8(4)
C(6)-C(5)-C(9)	118.6(4)
C(5)-C(6)-C(1)	119.6(4)
C(5)-C(6)-C(7)	120.4(4)
C(1)-C(6)-C(7)	120.0(4)
O(3)-C(7)-O(4)	116.9(4)
O(3)-C(7)-C(6)	123.1(4)
O(4)-C(7)-C(6)	119.9(4)
O(4)-C(8)-C(9)	111.3(4)
O(4)-C(8)-C(10)	107.0(4)
C(9)-C(8)-C(10)	112.0(4)
O(4)-C(8)-H(8)	108.8
C(9)-C(8)-H(8)	108.8
C(10)-C(8)-H(8)	108.8
C(5)-C(9)-C(8)	111.3(4)
C(5)-C(9)-H(9A)	109.4
C(8)-C(9)-H(9A)	109.4
C(5)-C(9)-H(9B)	109.4
C(8)-C(9)-H(9B)	109.4
H(9A)-C(9)-H(9B)	108.0
C(8)-C(10)-C(11)	115.0(5)
C(8)-C(10)-H(10A)	108.5
C(11)-C(10)-H(10A)	108.5
C(8)-C(10)-H(10B)	108.5
C(11)-C(10)-H(10B)	108.5
H(10A)-C(10)-H(10B)	107.5
C(12)-C(11)-C(10)	111.3(7)
C(12)-C(11)-H(11A)	109.4
C(10)-C(11)-H(11A)	109.4
C(12)-C(11)-H(11B)	109.4
C(10)-C(11)-H(11B)	109.3
H(11A)-C(11)-H(11B)	108.0
C(13)-C(12)-C(11)	124.6(8)
C(11)-C(12)-C(13')	109.6(9)
C(13)-C(12)-H(12A)	106.0
C(11)-C(12)-H(12A)	105.5
C(13)-C(12)-H(12B)	106.4
C(11)-C(12)-H(12B)	106.7
H(12A)-C(12)-H(12B)	106.5
C(11)-C(12)-H(12C)	109.4
C(13')-C(12)-H(12C)	109.4
C(11)-C(12)-H(12D)	109.0
C(13')-C(12)-H(12D)	109.8
H(12C)-C(12)-H(12D)	109.7
C(13)-O(5)-H(5)	110.1
O(5)-C(13)-C(12)	100.8(12)
O(5)-C(13)-C(14)	110.8(15)
C(12)-C(13)-C(14)	113.1(13)
O(5)-C(13)-H(13)	110.7
C(12)-C(13)-H(13)	110.6
C(14)-C(13)-H(13)	110.6
C(13)-C(14)-H(14A)	109.5
C(13)-C(14)-H(14B)	109.4
H(14A)-C(14)-H(14B)	109.5
C(13)-C(14)-H(14C)	109.5
H(14A)-C(14)-H(14C)	109.5
H(14B)-C(14)-H(14C)	109.5
C(13')-O(5')-H(5')	109.5
O(5')-C(13')-C(14')	106.6(14)
O(5')-C(13')-C(12)	124.0(12)
C(14')-C(13')-C(12)	112.2(13)
O(5')-C(13')-H(13')	104.0
C(14')-C(13')-H(13')	104.0
C(12)-C(13')-H(13')	103.9
C(13')-C(14')-H(14D)	109.5
C(13')-C(14')-H(14E)	109.4
H(14D)-C(14')-H(14E)	109.5
C(13')-C(14')-H(14F)	109.5
H(14D)-C(14')-H(14F)	109.5
H(14E)-C(14')-H(14F)	109.5
_____________________________________________________________ 
Symmetry transformations used to generate equivalent atoms: 
 

 Table 4.   Anisotropic displacement parameters  (Å2x 103) for cu_dd18259_0m.  The anisotropic
displacement factor exponent takes the form:  -2p2[ h2 a*2U11 + ...  + 2 h k a* b* U12 ]
______________________________________________________________________________ 
	U11	U22 	U33	U23	U13	U12
______________________________________________________________________________ 
O(1)	74(2) 	43(2)	65(2) 	5(2)	40(2) 	3(2)
O(2)	53(2) 	51(2)	47(2) 	3(2)	32(1) 	4(2)
O(3)	49(2) 	49(2)	45(2) 	2(1)	27(1) 	1(2)
O(4)	48(2) 	46(2)	49(2) 	0(1)	26(2) 	5(1)
C(1)	45(2) 	40(2)	41(2) 	-4(2)	19(2) 	-1(2)
C(2)	59(3) 	38(2)	51(3) 	-6(2)	25(2) 	5(2)
C(3)	46(2) 	52(3)	43(2) 	-6(2)	23(2) 	3(2)
C(4)	35(2) 	47(2)	36(2) 	-1(2)	13(2) 	0(2)
C(5)	34(2) 	44(2)	32(2) 	-2(2)	15(2) 	-1(2)
C(6)	40(2) 	42(2)	31(2) 	0(2)	11(2) 	-4(2)
C(7)	35(2) 	46(3)	36(2) 	1(2)	12(2) 	-1(2)
C(8)	50(3) 	43(2)	37(2) 	-1(2)	18(2) 	3(2)
C(9)	47(2) 	44(2)	39(2) 	2(2)	21(2) 	4(2)
C(10)	72(4) 	46(3)	56(3) 	7(2)	33(3) 	15(2)
C(11)	136(7) 	49(3)	86(5) 	-1(3)	69(5) 	22(4)
C(12)	108(6) 	49(3)	115(6) 	11(3)	68(5) 	12(4)
O(5)	109(6) 	77(5)	91(6) 	-7(5)	27(5) 	25(5)
C(13)	97(6) 	56(4)	84(6) 	4(4)	43(5) 	18(5)
C(14)	102(8) 	66(6)	96(8) 	-2(6)	46(7) 	20(6)
O(5')	119(7) 	84(6)	106(7) 	-5(5)	36(6) 	15(5)
C(13')	98(6) 	59(5)	84(6) 	4(5)	44(5) 	13(5)
C(14')	101(7) 	58(5)	86(6) 	-7(5)	47(5) 	20(5)
______________________________________________________________________________ 
 Table 5.   Hydrogen coordinates ( x 104) and isotropic  displacement parameters (Å2x 10 3)
for cu_dd18259_0m.
________________________________________________________________________________ 
	x 	y 	z 	U(eq)
________________________________________________________________________________ 
 
H(1)	3520	3423	7975	85
H(2)	9670	4468	414	71
H(2A)	7635	3033	4299	57
H(3)	9385	3603	1776	54
H(8)	6249	5302	7543	50
H(9A)	3581	5145	2665	50
H(9B)	6708	5301	3598	50
H(10A)	1462	5814	4850	66
H(10B)	4387	6088	5514	66
H(11A)	1103	5821	8926	100
H(11B)	4101	6066	9670	100
H(12A)	475	6657	6319	101
H(12B)	2766	6874	8368	101
H(12C)	2352	6843	7182	101
H(12D)	-631	6607	7082	101
H(5)	-680	6606	12318	137
H(13)	-2602	6632	8817	91
H(14A)	-2917	7558	9507	127
H(14B)	-2107	7488	6980	127
H(14C)	165	7646	9249	127
H(5')	4783	6750	12187	151
H(13')	10	6651	11247	92
H(14D)	-23	7601	11802	117
H(14E)	-2298	7353	9733	117
H(14F)	301	7642	9104	117
________________________________________________________________________________ 
 Table 6.  Torsion angles [°] for cu_dd18259_0m.
________________________________________________________________ 
O(1)-C(1)-C(2)-C(3)	179.3(5)
C(6)-C(1)-C(2)-C(3)	0.1(8)
C(1)-C(2)-C(3)-C(4)	-1.0(8)
C(2)-C(3)-C(4)-O(2)	-177.9(4)
C(2)-C(3)-C(4)-C(5)	1.1(7)
O(2)-C(4)-C(5)-C(6)	178.9(4)
C(3)-C(4)-C(5)-C(6)	-0.2(6)
O(2)-C(4)-C(5)-C(9)	0.6(6)
C(3)-C(4)-C(5)-C(9)	-178.5(4)
C(4)-C(5)-C(6)-C(1)	-0.7(6)
C(9)-C(5)-C(6)-C(1)	177.6(4)
C(4)-C(5)-C(6)-C(7)	-178.6(4)
C(9)-C(5)-C(6)-C(7)	-0.3(6)
O(1)-C(1)-C(6)-C(5)	-178.4(4)
C(2)-C(1)-C(6)-C(5)	0.8(7)
O(1)-C(1)-C(6)-C(7)	-0.6(7)
C(2)-C(1)-C(6)-C(7)	178.7(4)
C(8)-O(4)-C(7)-O(3)	162.0(4)
C(8)-O(4)-C(7)-C(6)	-20.0(6)
C(5)-C(6)-C(7)-O(3)	173.3(4)
C(1)-C(6)-C(7)-O(3)	-4.6(7)
C(5)-C(6)-C(7)-O(4)	-4.6(6)
C(1)-C(6)-C(7)-O(4)	177.6(4)
C(7)-O(4)-C(8)-C(9)	47.0(6)
C(7)-O(4)-C(8)-C(10)	169.7(4)
C(4)-C(5)-C(9)-C(8)	-154.8(4)
C(6)-C(5)-C(9)-C(8)	26.9(5)
O(4)-C(8)-C(9)-C(5)	-48.3(5)
C(10)-C(8)-C(9)-C(5)	-168.0(5)
O(4)-C(8)-C(10)-C(11)	58.2(8)
C(9)-C(8)-C(10)-C(11)	-179.6(6)
C(8)-C(10)-C(11)-C(12)	176.7(7)
C(10)-C(11)-C(12)-C(13)	146.4(12)
C(10)-C(11)-C(12)-C(13')	-166.6(10)
C(11)-C(12)-C(13)-O(5)	56.1(17)
C(13')-C(12)-C(13)-O(5)	-26.6(11)
C(11)-C(12)-C(13)-C(14)	174.3(15)
C(13')-C(12)-C(13)-C(14)	91.6(19)
C(13)-C(12)-C(13')-O(5')	178(2)
C(11)-C(12)-C(13')-O(5')	57.9(17)
C(13)-C(12)-C(13')-C(14')	-51.8(14)
C(11)-C(12)-C(13')-C(14')	-171.8(12)
________________________________________________________________ 
Symmetry transformations used to generate equivalent atoms: 
 

 Table 7.  Hydrogen bonds for cu_dd18259_0m  [Å and °].
____________________________________________________________________________ 
D-H...A	d(D-H)	d(H...A)	d(D...A)	<(DHA)
____________________________________________________________________________ 
 
 
